# Travel-related respiratory symptoms and infections in travellers (2000–22): a systematic review and meta-analysis

**DOI:** 10.1093/jtm/taad081

**Published:** 2023-06-13

**Authors:** Thibault Lovey, Robin Hasler, Philippe Gautret, Patricia Schlagenhauf

**Affiliations:** Epidemiology, Biostatistics and Prevention Institute, University of Zürich, Hirschengraben 84, 8001 Zürich Switzerland; HFR Fribourg – Cantonal Hospital, 1708 Fribourg, Switzerland; IHU-Méditerranée Infection, 13005 Marseille, France; Epidemiology, Biostatistics and Prevention Institute, University of Zürich, Hirschengraben 84, 8001 Zürich Switzerland; Department of Global and Public Health, MilMedBiol Competence Centre, Epidemiology Biostatistics and Prevention Institute, WHO Collaborating Centre for Travellers’ Health, Hirschengraben 84, 8001 Zürich, Switzerland

**Keywords:** Communicable diseases, respiratory tract infections, prevalence, travel, risk factors

## Abstract

**Background:**

Respiratory tract infections (RTIs) are common in travellers due to the year-round or seasonal presence of respiratory pathogen and exposure to crowded environments during the itinerary. No study has systematically examined the burden of RTI infections among travellers. The aim of this systematic review and meta-analysis is to evaluate the prevalence of RTIs and symptoms suggestive of RTIs among travellers according to risk groups and/or geographic region, and to describe the spectrum of RTIs.

**Methods:**

The systematic review and meta-analysis was registered in PROSPERO (CRD42022311261). We searched Medline, Embase, Scopus, Cochrane Central, Web of Science, Science Direct and preprint servers MedRxiv, BioRxiv, SSRN and IEEE Xplore on 1 February 2022. Studies reporting RTIs or symptoms suggestive of RTIs in international travellers after 1 January 2000 were eligible. Data appraisal and extraction were performed by two authors, and proportional meta-analyses were used to obtain estimates of the prevalence of respiratory symptoms and RTIs in travellers and predefined risk groups.

**Findings:**

A total of 429 articles on travellers’ illness were included. Included studies reported 86 841 symptoms suggestive of RTIs and 807 632 confirmed RTIs. Seventy-eight percent of reported respiratory symptoms and 60% of RTIs with available location data were acquired at mass gatherings events. Cough was the most common symptom suggestive of respiratory infections, and the upper respiratory tract was the most common site for RTIs in travellers. The prevalence of RTIs and respiratory symptoms suggestive of RTIs were 10% [8%; 14%] and 37% [27%; 48%], respectively, among travellers. Reporting of RTIs in travellers denoted by publication output was found to correlate with global waves of new respiratory infections.

**Interpretation:**

This study demonstrates a high burden of RTIs among travellers and indicates that travellers’ RTIs reflect respiratory infection outbreaks. These findings have important implications for understanding and managing RTIs among travellers.

## Introduction

After a 70% drop in traveller arrivals in 2019 due to the COVID-19 pandemic, international travel appears to be rapidly recovering. Most countries have relaxed their entry requirements and reopened their borders. The UN World Tourism Organization reports that arrivals in the first 7 months of 2022 have reached 57% of pre-pandemic levels, due in part to increased vaccination rates and a manageable number of COVID cases.^1^

Although the COVID-19 pandemic has turned into an endemic, non-severe infection in most areas, the risk of respiratory infectious disease persists. Exposure to crowded environments, such as encountered during transportation, sightseeing, mass gatherings, and the year-round or seasonal presence of respiratory pathogens in frequently visited areas make travellers particularly vulnerable to respiratory infections. The full spectrum of travel-related respiratory illness is rarely described and studies are dated.^2^^,^^3^ The estimated prevalence of respiratory symptoms among travellers varies widely. For example, it ranges from less than 1% among travellers arriving at US airports to more than 39% among French medical students travelling abroad and up to 93% of Hajj pilgrims.^4–6^ The prevalence is equally high in confirmed respiratory tract infections (whether diagnosed medically or by molecular methods).

RTIs in travellers often resemble those in the local population, and tropical respiratory illnesses remain rare in travellers.^7^^,^^8^ Thus, as in the general population, upper respiratory tract infections (URTIs) are more common than lower respiratory tract infections (LRTIs).^9^ Pathogens circulating in the local population, such as respiratory viruses, are frequently detected in travellers.^8^ Influenza is the most common virus, with an estimated prevalence of 2.8% and up to 15% among travellers with fever.^10–13^ Travellers are particularly affected by the H3N2 variant and to a lesser extent by the H1N1 or B group variants.^10^ Other viruses commonly found in travellers include rhinoviruses, adenoviruses and respiratory syncytial virus type A.^14^ Typical or atypical bacteria are also common and fungi are sometimes detected, notably histoplasmosis.^15^

Certain groups of travellers or specific locations have an increased risk of respiratory infections. For example, tourists have a higher risk of respiratory infections than immigrants, visitors of friends and relatives (VFRs), and expatriates (*P* < 0.0001).^16^ Outbreaks of respiratory infections are commonly reported in relation to air travel or on ships with their confined spaces.^17–21^ Respiratory infections also occur at mass gatherings, especially religious and sporting events with large crowds, and account for up to 40% of all illnesses reported during such events.^22^ However, some respiratory infections are limited to certain travel groups.

To date, there are no systematic reviews or global studies that provide information on the complete range of respiratory illness in travellers by specific groups of travellers. We therefore aimed to conduct a systematic review and meta-analysis of studies published between 2000 and 2022, taking into account different regions and risk groups to obtain reliable prevalences. The main objective of this study was to estimate the prevalence of respiratory symptoms and RTIs among all travellers stratified by specific regions and/or risk groups. As a secondary objective, we examined the types of symptoms indicative of RTIs and confirmed RTIs that occurred in travellers and assessed the difference in risk for the two important factors of age and sex.

## Methods

This systematic review follows the PRISMA 2020 guidelines and has been registered in the PROSPERO database (CRD42022311261).

### Eligibility criteria

Studies that reported at least one case of respiratory infection or respiratory symptoms in travellers were included.

Respiratory infections or respiratory tract infection (RTIs) were broadly defined as infections affecting at least one segment of the respiratory tract (either as the site of infection or the route of transmission). Only confirmed respiratory infections were considered, i.e. cases diagnosed either by clinical examination (including history, clinical examination and complementary tests) or by molecular methods (such as PCR or serology). Respiratory infections caused by viruses, bacteria including mycobacteriaceae, and fungi were evaluated.

In addition, respiratory symptoms were defined as a group of symptoms suggestive of the presence of RTIs. Cough, dyspnoea, expectoration, loss of sense of smell or taste, rhinitis/runny nose/congestion, sinus pain, sore throat, voice failure/hoarseness and wheezing were considered symptoms of RTIs in our article.

Only adult travellers who had crossed at least one international border were studied. Cases of respiratory infections or symptoms that occurred during specific occasions such as mass gatherings, air travel, cruises or on commercial vessels were considered and stratified by specific groups. Considering the limited number of reported respiratory infections among participants in various mass events outside the Hajj (Saudi Arabia), including events such as AsiaWorld-Expo (Hong Kong SAR), Bābā Farīd (Pakistan), Easter Festival (Austria), Eco-Challenge (multiple countries), EXIT Festival (Serbia), Grand Magal of Touba (Senegal), Rock Werchter (Belgium), Sojourn (Germany), Sziget Festival (Hungary), Tablighi Jamaat (India), Umrah (Saudi Arabia), Universiade (Serbia), Winter Olympics and Paralympics (2002, USA) and World Youth Day (2008, Australia), we grouped them into a single risk category in our study and referred to them as *Mass gathering events*. For air travel and cruise ships, no distinction was made between crew members and other passengers. Special categories were also defined for refugees and asylum seekers. Settled immigrants, already established in the country of reporting, were excluded. Studies that used genome sequencing or mathematical models to estimate the country in which respiratory infection was acquired or to predict new cases were excluded.

Studies in English, French, Spanish and German that reported cases between 1 January 2000 and 31 January 2022 were reviewed, including those whose recruitment period began before 2000 but included cases from the period of interest. Preprints, conference abstracts and peer-reviewed studies such as randomized controlled trials (RCTs), cohort studies, case–control studies, cross-sectional studies, case series, case reports, prevalence studies and systematic reviews were considered for eligibility. Reviews, editorials, opinion pieces, essay summaries, books and articles for which full text was not available were excluded. Commentaries, short communications and letters to the editor that did not report original cases were also excluded.

### Information sources

A systematic search was conducted on 1 February 2022 in the bibliographic databases Medline, Embase, Scopus, Cochrane Central Register of Controlled Trials (CENTRAL), Web of Science, Science Direct and the preprint servers MedRxiv, BioRxiv, SSRN and IEEE Xplore (TL). All strings were recorded and can be found in the Appendix (section 1).

### Selection process

The results of the various strings were imported into Endnote20 Reference Manager (Clavirate, Boston, MA 02210) to achieve deduplication. The references were then transferred to the knowledge synthesis software Rayyan QCRI. All studies were checked for eligibility first by title and abstract and then by full text. All references of included studies and excluded reviews were also reviewed. The screening processes were performed simultaneously by two different blinded authors (T.L., R.H.). Pooling was performed at the end of each round, and a third author (P.S.) was involved in the decision in case of disagreement. Reasons for exclusion were recorded for each excluded study in Rayyan QRCI and reported in the PRISMA flowchart.

### Data collection

For each article in which at least one respiratory symptom and/or confirmed respiratory infection was reported, the total number, the sex stratification, the age group, the size of the study population, and the type of respiratory symptom or respiratory infection and possible accompanying symptoms were recorded. The country where the respiratory symptom or infectious respiratory disease was detected and the location where it was acquired were also noted. Age groups were defined in four categories, Child/Youth (0–19 years) to accommodate included papers where young adults were included in ‘child’ categories, Adult (20–39 years), Middle-aged Adult (40–59 years) and Senior Adult (60+ years). When only the percentage or prevalence per 1000 persons was reported, the data were converted to absolute numbers. If this was not possible, the study was excluded. The extraction process was performed manually twice per study in two different Google sheets before they were merged (T.L.).

### Bias assessment

We used the JBI Critical Appraisal Tools^23^ to evaluate our studies. These tools were developed by an independent, non-profit research organization at the Faculty of Health and Medical Sciences, University of Adelaide, Australia, and include a specific checklist for each study design. Studies were scored simultaneously by two blinded authors (T.L., R.H.), and a mean score was calculated. Scores are expressed as a percentage of the maximum possible score and can therefore be easily compared between different types of studies. Interpretations in the literature differ, but it is reasonable to assume that a score above 70% indicates a low risk of bias, a score between 50% and 70% indicates a medium risk of bias, and a score below 50% indicates a high risk of bias.

### Data synthesis

All studies that met the inclusion criteria were included in the descriptive analysis. The absolute number of cases of respiratory infections, respiratory symptoms, and the number of cases by UN region or specific category and the distribution by sex and age were calculated. Maps were generated for respiratory infections, respiratory symptoms and major respiratory infection outbreaks in the past 20 years (H1N1, MERS-CoV, SARS-CoV-1, SARS-CoV-2), with absolute numbers of cases calculated for the 17 subregions of the UN geoscheme. Studies that reported only positive cases, had no reference population or whose reference population was not exclusively travellers were excluded from the meta-analysis. No studies were excluded due to a high risk of bias—only those identified by three unsupervised learning algorithms (k-means, DBSCAN and Gaussian mixture models) in the graphic display of study heterogeneity (GOSH) graphs. The meta-analysis of proportions was calculated with a logistic regression model using the maximum likelihood estimator for tau^2^, the Hartung–Knapp adjustment for the random effects model and a logit transformation. A significance level of 0.05 was used for all statistical tests. All analyses were performed with the statistical program RStudio (version 2022.07.1).^24^

### Role of the funding source

There was no funding source for this study.

## Results

**Figure 1:**
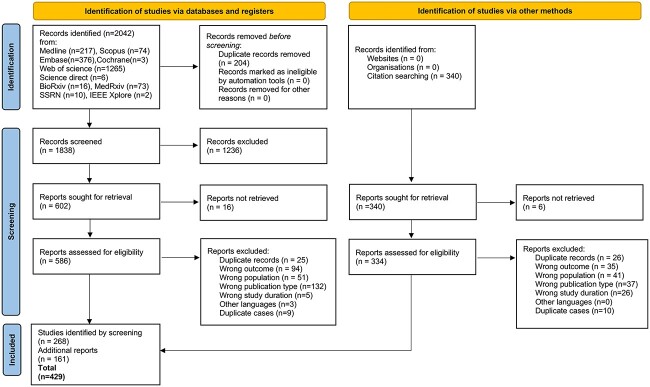
PRISMA flow diagram 2020

Database searches yielded 2042 articles, of which 204 were identified as duplicates and 1234 were excluded after review of titles and abstracts. Of the remaining 602 articles, 19 full texts could not be found and 583 were screened for eligibility. After the second screening, 268 articles were included. Based on their references and the references of the excluded journals, 340 new studies were identified. Of these, an additional 161 studies were included, bringing the total number of included studies to 429. An overview of the screening process and reasons for exclusion during full-text screening is provided in Figure 1 and the full list of included studies can be found in the Appendix (section 2).^25^

**Figure 2:**
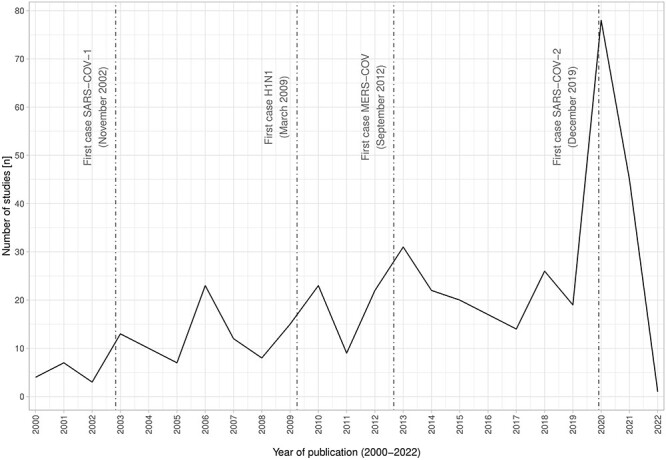
Time-series plot of the absolute annual frequency of included studies with the date of the first observed case of the four epidemics/pandemics of respiratory infections of the twenty-first century

The year of publication of included studies ranged from 2000 to January 2022, with most studies (*n* = 78) coming from 2020 and fewest from 2002 containing the fewest (*n* = 3). The year 2022 cannot be compared because it included only the month of January. The largest difference between years was 2019 (*n* = 19) and 2020 (*n* = 78) with four times more studies than the previous year due to a large number of 2020 COVID-19-related publications. Figure 2 shows the number of included studies per year compared with the date of the first observed case of the four epidemics/pandemics of respiratory infections of the twenty-first century. Reporting of RTIs in travellers denoted by publication output *s* was found to correlate closely with global waves of new respiratory infections.

Of the 429 included studies, more than 66% (284/429) were cross-sectional studies and the remainder were RCTs (*n* = 2), cohort studies (*n* = 17), case–control studies (*n* = 2), case reports (*n* = 57), case series (*n* = 55) and prevalence studies (*n* = 12). The overall mean for the JBI critical score was 77% (SD 15), with a minimum score of 25% and maximum score of 100%. Randomized control trials have the lowest mean for the JBI critical score with 63% (SD 3) and case–control studies the highest one with 92% (SD 11). The study with the minimal JBI score overall 25% was a case report (Table 1).

**Table 1:** Table showing the total number of studies by study design JBI score, number of respiratory cases and symptoms, and distribution by region, specific group, sex and age category

Characteristic	Overall		RCTs		Cohort studies		Cross-sectional studies		Case–control studies		Case reports		Case series		Prevalence studies	
Number of studies (*n*)	429		2		17		284		2		57		55		12	
JBI critical score (%)																
Mean (SD)	77 (15)		63 (3)		72 (9)		78 (14)		92 (11)		77 (19)		73 (15)		78 (16)	
Range	25, 100		62, 65		59, 91		44, 100		85, 100		25, 100		45, 100		39, 100	
	Symptoms	Cases	Symptoms	Cases	Symptoms	Cases	Symptoms	Cases	Symptoms	Cases	Symptoms	Cases	Symptoms	Cases	Symptoms	Cases
Total (*n*)	86 841	807 632	676	324	499	2773	82 791	798 451	1310	32	61	90	290	2863	1214	3099
UN Region																
Africa	250 (2%)^b^	4233 (3%)^b^	N/A^b^	N/A^b^	N/A^b^	284 (24%)^b^	219 (2%)^b^	3872 (3%)^b^	N/A^b^	N/A^b^	7 (12%)^b^	13 (14%)^b^	24 (10%)^b^	64 (3%)^b^	N/A^b^	N/A^b^
Americas	1166 (8%)^b^	5263 (4%)^b^	N/A^b^	N/A^b^	N/A^b^	59 (5%)^b^	524 (5%)^b^	4955 (4%)^b^	N/A^b^	N/A^b^	12 (20%)^b^	20 (21%)^b^	13 (5%)^b^	201 (9%)^b^	617 (51%)^b^	28 (3%)^b^
Asia	687 (5%)^b^	19 812 (14%)^b^	N/A^b^	N/A^b^	N/A^b^	195 (16%)^b^	632 (6%)^b^	18 242 (14%)^b^	N/A^b^	N/A^b^	32 (54%)^b^	34 (36%)^b^	23 (10%)^b^	1340 (57%)^b^	N/A^b^	1 (0%)^b^
Europe	59 (0%)^b^	11 167 (8%)^b^	N/A^b^	N/A^b^	N/A^b^	93 (8%)^b^	14 (0%)^b^	10 485 (8%)^b^	N/A^b^	N/A^b^	2 (3%)^b^	3 (3%)^b^	43 (18%)^b^	586 (25%)^b^	N/A^b^	N/A^b^
Oceania	1 (0%)^b^	788 (1%)^b^	N/A^b^	N/A^b^	N/A^b^	N/A^b^	N/A^b^	787 (1%)^b^	N/A^b^	N/A^b^	1 (2%)^b^	1 (1%)^b^	N/A^b^	N/A^b^	N/A^b^	N/A^b^
Specific cases																
Airplane	93^b^	451^b^	N/A^b^	N/A^b^	N/A^b^	9^b^	47^b^	375^b^	N/A^b^	N/A^b^	N/A^b^	1^b^	17^b^	37^b^	29^b^	29^b^
Cruise or Merchant Vessel	465 (3%)^b^	2478 (2%)^b^	N/A^b^	N/A^b^	N/A^b^	N/A^b^	351 (3%)^b^	1629 (1%)^b^	N/A^b^	N/A^b^	3 (5%)^b^	3 (3%)^b^	111 (47%)^b^	56 (2%)^b^	N/A^b^	790 (93%)^b^
Refugee and Asylum-Seeker	341 (2%)^b^	11 179 (8%)^b^	N/A^b^	N/A^b^	N/A^b^	N/A^b^	340 (3%)^b^	11 157 (8%)^b^	N/A^b^	N/A^b^	1 (2%)^b^	3 (3%)^b^	N/A^b^	19 (1%)^b^	N/A^b^	N/A^b^
Mass gatherings events^a^	11 033 (78%)^b^	81 862 (60%)^b^	676 (100%)^b^	337 (100%)^b^	248 (100%)^b^	542 (46%)^b^	8229 (79%)^b^	80 897 (61%)^b^	1310 (100%)^b^	32 (100%)^b^	1 (2%)^b^	16 (17%)^b^	7 (3%)^b^	38 (2%)^b^	562 (47%)^b^	N/A^b^
Age distribution																
Child/Young	50 (3%)^b^	8359 (34%)^b^	N/A^b^	N/A^b^	N/A^b^	N/A^b^	48 (4%)^b^	8337 (34%)^b^	N/A^b^	N/A^b^	1 (2%)^b^	1 (2%)^b^	1 (1%)^b^	21 (10%)^b^	N/A^b^	N/A^b^
Adult	202 (13%)^b^	7928 (32%)^b^	N/A^b^	N/A^b^	12 (57%)^b^	NA (NA%)^b^	135 (10%)^b^	7816 (32%)^b^	N/A^b^	N/A^b^	15 (29%)^b^	18 (31%)^b^	40 (49%)^b^	94 (47%)^b^	N/A^b^	N/A^b^
Middle-Aged Adult	108 (7%)^b^	5318 (22%)^b^	N/A^b^	N/A^b^	N/A^b^	N/A^b^	65 (5%)^b^	5250 (22%)^b^	N/A^b^	N/A^b^	19 (37%)^b^	20 (34%)^b^	24 (30%)^b^	48 (24%)^b^	N/A^b^	N/A^b^
Senior Adult	1161 (76%)^b^	2844 (12%)^b^	N/A^b^	N/A^b^	9 (43%)^b^	N/A^b^	1119 (82%)^b^	2769 (11%)^b^	N/A^b^	18 (100%)^b^	17 (33%)^b^	20 (34%)^b^	16 (20%)^b^	37 (18%)^b^	N/A^b^	N/A^b^
Unknown	85322^b^	783183^b^	676^b^	324^b^	478^b^	2773^b^	81424^b^	774279^b^	1310^b^	14^b^	11^b^	31^b^	209^b^	2663^b^	1214^b^	3099^b^
Gender distribution																
Female	858 (47%)^b^	18 822 (40%)^b^	N/A^b^	N/A^b^	N/A^b^	106 (26%)^b^	789 (47%)^b^	18 541 (40%)^b^	N/A^b^	12 (38%)^b^	17 (33%)^b^	20 (33%)^b^	52 (44%)^b^	143 (37%)^b^	N/A^b^	N/A^b^
Male	986 (53%)^b^	28 220 (60%)^b^	N/A^b^	N/A^b^	N/A^b^	303 (74%)^b^	886 (53%)^b^	27 609 (60%)^b^	N/A^b^	20 (62%)^b^	35 (67%)^b^	41 (67%)^b^	65 (56%)^b^	247 (63%)^b^	N/A^b^	N/A^b^
Unknown	85 025^b^	760 590^b^	676^b^	324^b^	499^b^	2364^b^	81142^b^	752 301^b^	1310^b^	N/A^b^	11^b^	29^b^	173^b^	2473^b^	1214^b^	3099^b^

^a^AsiaWorld-Expo, Bābā Farīd, Easter Festival (Carinthia), Eco-Challenge, EXIT Festival, Grand Magal of Touba, Hajj, Rock Werchter, Sojourn, Sziget Festival, Tablighi Jamaat, Umrah, Universiade, Winter Olympic & Paralympic Games, World Youth Day

^b^Relative frequency (%)

Included studies reported 86 841 symptoms suggestive of RTIs and 807 632 confirmed RTIs. Of the reported respiratory symptoms with available information on the area of acquisition, 78% (11 033/14 095) occurred at mass gathering events. The most represented UN region for respiratory symptom acquisition was the Americas with 8% (1166/14095), while the Oceania region reported only one respiratory symptom in a case report. Respiratory symptoms were linked to airplanes, cruise ships/commercial vessels and refugees/asylum seekers in 93, 465 and 341 travellers, respectively. Of the confirmed respiratory illness cases with known area of acquisition, 60% (81 862/137233) were from mass gatherings events and the most represented UN region of acquisition was Asia where 14% (19 812/137 233) of respiratory cases were acquired. Oceania was the least represented UN region with only 1% (788/137 233) of confirmed cases. There were 451, 2478 and 11 179 reported respiratory infection cases linked to airplanes, cruise ships/commercial vessels and refugees/asylum seekers, respectively. The sex ratio for respiratory symptoms was 1.15 male:1.00 female (men: 986/women: 858) and for respiratory infections this was 1.50 male and 1.00 female (men: 28 220; women: 18 822). Regarding age categories, 76% (1161/1521) of reported symptoms were in older adults (>60 years), whereas for respiratory infection cases the distribution was balanced among age categories. Missing data were common in the included studies particularly for sex and age. Place of acquisition was not reported in 84% (72 746/86841) of reported respiratory symptoms, as well as in 83% (670 399/807632) of reported respiratory tract infection cases. A high rate of missing data was found for both respiratory symptoms and confirmed RTI cases. Specifically, 98% (85 322/86843) of the data was missing for the age category and 98% (85 025/86869) for sex in respiratory symptoms. Similarly, 97% (783 183/807632) of the data was missing for the age category and 94% (760 590/807632) for sex in confirmed RTI cases (Table 1).

**Figure 3:**
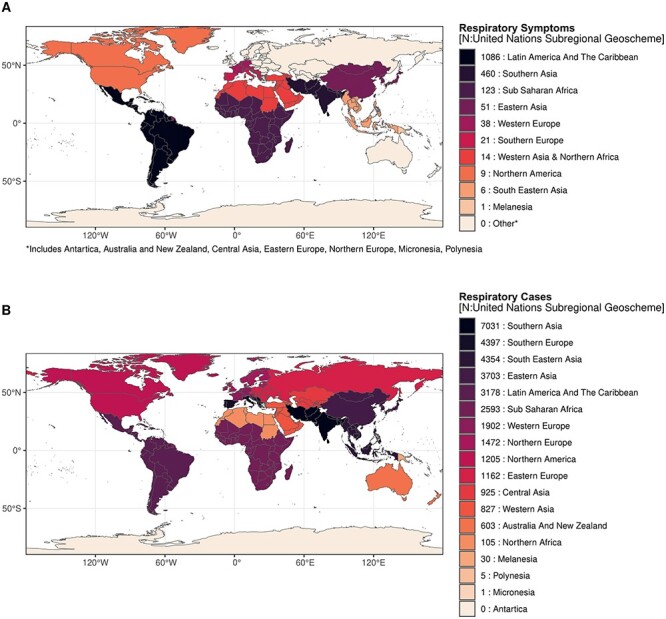
Cumulative cases from 2000 to 2022 for symptoms suggestive of respiratory illness (A) and respiratory infections (B) in the 17 United Nations subregional geoschemes. *Note*: Specific groups (Airplane, Cruise or Merchant Vessel, Refugee and Asylum-Seeker and Mass gatherings events) are not displayed

Of the 2163 respiratory symptoms for which UN regional location assignment was possible, 84% (1809/2163) could be assigned to the 17 UN subregional geoschemes. Latin America and the Caribbean was the subregion with the most travel-related respiratory symptoms, with 60% (1086/1809) of reported respiratory symptoms. No reports of travel-related respiratory symptoms were found for other subregions such as Australia and New Zealand, Central Asia, Eastern Europe, Northern Europe, Micronesia, Polynesia and Antarctica (Figure 3A). Looking at respiratory tract infections, of the 41 263 cases for which UN regional location was available, 81% (33 493/41 263) could be attributed to the 17 UN subregional geoschemes. For RTIs, South Asia was the subregion with the most reported cases of respiratory illness among travellers, and Micronesia and Antarctica were the least affected regions, with 1 and 0 cases, respectively (Figure 3B).

Cough was the most common respiratory symptom with 11 206 reported cases among travellers, 76% of whom (8556/11 206) contracted the symptoms at mass gatherings. Fever was commonly associated with respiratory symptoms (4309). Common cold-like syndrome (CCLS) and Influenza-like Illness (ILI) were reported in 108 841 and 50 317 travellers, respectively. See the Appendix (Section 3) for more details.

Medically diagnosed respiratory infections accounted for 126 710 of the reported cases. Forty-nine percent (61 553/ 126 710) of these were URTI, followed by 29% (37 014/126 710) LRTI and 21% (26 632/126 710) mixed RTIs. Pharyngitis accounted for 51% (31 396/61 553) of LRTIs. Among LRTIs, pneumonia and tuberculosis were the most common, accounting for 43% (16 063/37 104) and 41% (15 136/37 104), respectively. More than 70% (10 542/15 136) of the TB cases were observed in the specific group of refugees and asylum seekers (Appendix section 4).

Respiratory infections detected by molecular diagnosis were mainly viruses (94%, 61 688/65 580), followed by bacteria (6%, 3863/65 580) and fungi (<1%, 29/65 580). *Coronaviridae* accounted for 54% (35 117/65 580) of the viruses detected, with SARS-CoV-2 accounting for 95% (33 258/35 117) of those. *Orthomyxoviridae* were also frequent (35%, 22 683/65 580), with H1N1 (A/H1N1) accounting for 22% (5091/22 683) of the influenza viruses detected. Fifty-seven percent (2219/3863) of the bacteria were Gram-negative, with *Haemophilus influenzae* accounting for 49% (1081/2219) of the Gram-negative bacteria and 28% (1081/3863) of the total bacteria detected. For fungi, *Candida albicans* was the most frequent (Appendix section 5).

Global maps showing the absolute frequency of publications related to respiratory infections in travellers in the 17 United Nations subregional geoschemes for the four respiratory epidemics/pandemics of the twenty-first century are provided in the Appendix (section 6).

### Meta-analysis

**Figure 4:**
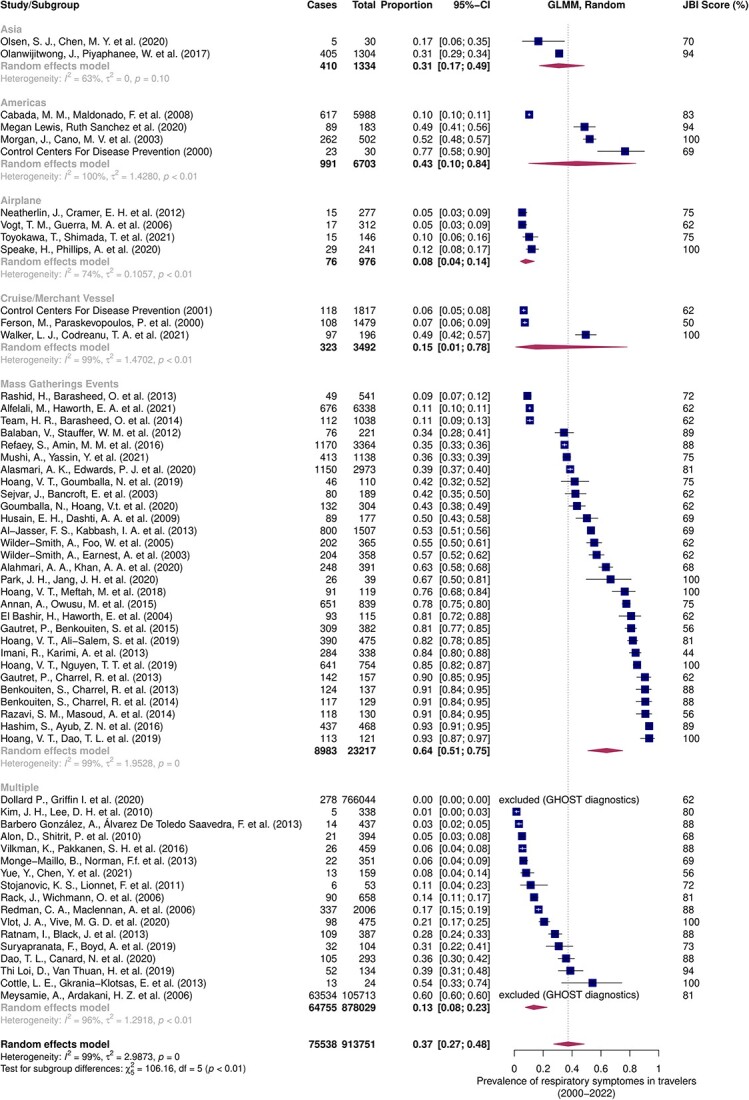
Meta-analysis estimating the prevalence of respiratory symptoms in travellers from 2000 to 2022 with subgroup analysis by UN region and specific groups

Fifty-nine studies were included in the meta-analysis for respiratory symptoms, of which two were eliminated by the graphic display of heterogeneity (GOSH) analysis.^4^^,^^6^^,^^15^^,^^26–81^ All included studies yielded a prevalence of respiratory symptoms in travellers of 37% [27%; 48%] for the years 2000 to 2022. Subgroup analysis shows that this estimate varies by exposure group, with the reported prevalence of respiratory symptoms in mass gatherings reaching 64% [51%; 75%], whereas after air travel only 8% [4%; 14%] of travellers had respiratory symptoms (Figure 4).

**Figure 5:**
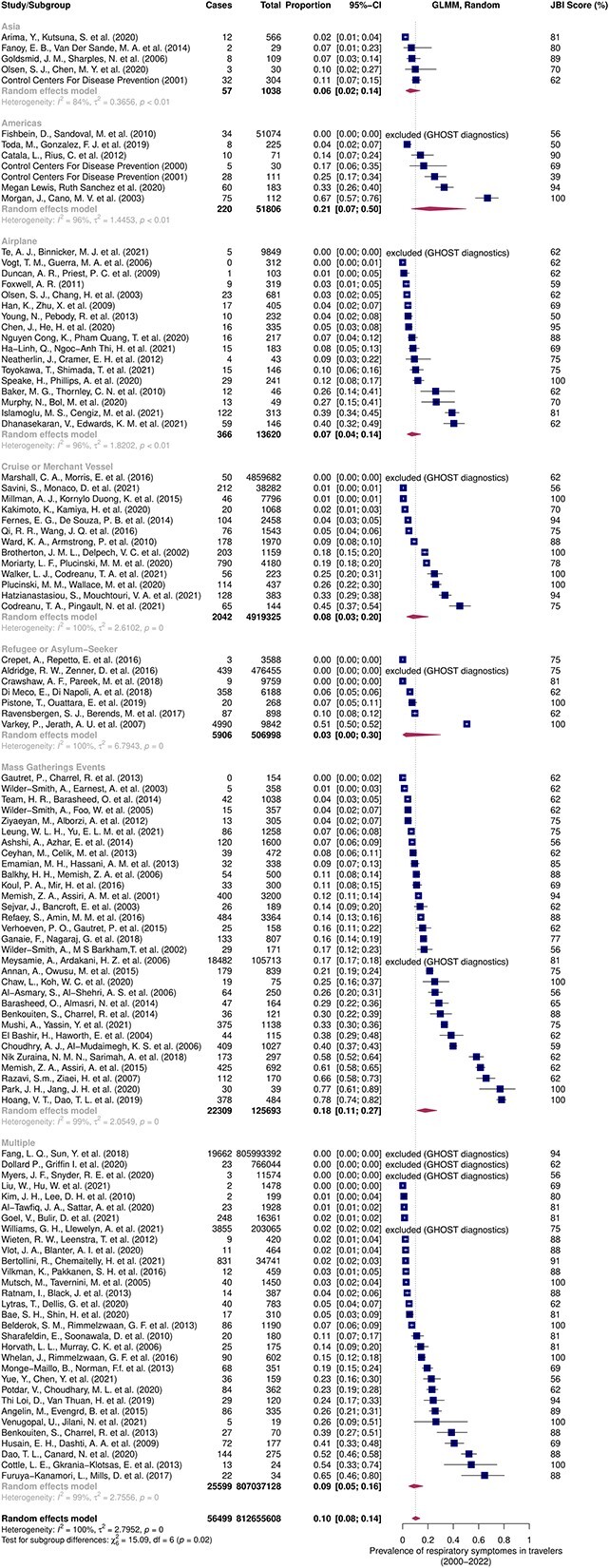
Meta-analysis estimating the prevalence of respiratory cases in travellers from 2000 to 2022 with subgroup analysis by UN region and specific groups

For respiratory infection cases, 111 studies were included in the meta-analysis, 9 of which were removed by the GOSH analysis.^4^^,^^6^^,^^10^^,^^15^^,^^17–20^^,^^22^^,^^28^^,^^31–37^^,^^40^^,^^43^^,^^45^^,^^46^^,^^49^^,^^51^^,^^53^^,^^54^^,^^56^^,^^58^^,^^59^^,^^64–66^^,^^69^^,^^72–74^^,^  ^77^^,^^79–154^ The prevalence of confirmed respiratory illness among travellers was estimated to be 10% [8%; 14%]. Americas and mass gatherings were the groups at highest risk, with 21% [7%; 50%] and 18% [11%; 27%], respectively. Asia had a lower-than-average risk of 6% [2%; 14%] (Figure 5).

## Discussion

This systematic review included 429 studies published between 2000 and January 2022 with an overall low risk of bias that reported respiratory symptoms or confirmed RTIs in travellers. We found a prevalence of respiratory symptoms and confirmed RTI cases of 37% [27%; 48%] and 10% [8%; 14%], respectively. This is the first meta-analysis to estimate global prevalences among travellers by region and specific area. These results demonstrate the high burden of respiratory infections among travellers regardless of risk group.

It is well known that mass gatherings are conducive to the spread of airborne diseases and that participants are therefore at high risk of suffering from respiratory symptoms and infections. In our study, the prevalence in mass gatherings was 1.7 and 1.8 higher than the overall prevalence for respiratory symptoms and infections, respectively, compared with other traveller groups. Furthermore, in absolute numbers, mass gatherings accounted for 60% of the reported cases of respiratory infections for which the area of acquisition was available, underscoring the risk of transmission during these events. Preventive measures, such as frequent changing of face masks, have been suggested to reduce respiratory transmission at mass gatherings. One study demonstrated a positive association between changing masks every 4 hours and fewer upper respiratory tract infections.^47^ However, other studies have failed to provide clear evidence of the effectiveness of face masks against viral respiratory infections in this setting, possibly because of non-compliance with protocols.^42^ In contrast to mass gatherings events, cases among airplane and cruise ship passengers were essentially limited to airborne viruses with SARS-CoV-2 and Influenza A virus accounting for most of the cases reported in our study.

The Americas, particularly the Latin America and Caribbean subregion, was the region with the highest absolute number of respiratory symptoms among travellers. The estimated prevalence of respiratory symptoms and confirmed RTIs is also higher than in other continents. However, the region with the highest absolute number of confirmed respiratory infections was Asia, particularly Southern Asia (Figure 3). This difference may be explained by the fact that mild respiratory symptoms or infections are underrepresented in the literature. Indeed, only 8–55% of travellers seek medical attention when they become ill during their trip, so mild respiratory symptoms or illnesses are more likely to go unreported.^155^ In addition, most studies are from developed countries, so reported cases depend on the preferred destinations of these countries. In fact, Asia is the second most visited continent after Europe, with 360 million international tourist arrivals in 2019.

Most URTIs are caused by viral pathogens. In the last 22 years, 94% of the causative agents of RTIs in travellers reported in the literature were viruses. It is therefore not surprising that 49% of medically diagnosed infections in our studies were URTIs, compared with 29% LRTIs. Fever was found to be frequently associated with respiratory symptoms, confirming the findings of many studies, namely that respiratory infections are the most common cause of fever in travellers.^156–158^ Influenza-like illness (ILI), defined by the Centers for Disease Control and Prevention as fever (temperature of 37.8°C or higher) and cough and/or sore throat, was frequently observed in certain areas of increased risk for respiratory virus transmission, such as cruises and mass gatherings. For example, in a 3-year prospective study, 33% of ill passengers and crew members were diagnosed with ILI.^159^ In our systematic review, we found two cases of H5N1 infection in travellers. The first case was reported in November 2010 and involved a traveller returning from a poultry market in Shanghai.^160^ The second case was reported in December 2013 and involved a Canadian traveller who had recently returned from a 3-week stay in Beijing.^161^ Avian Influenza Weekly Update number 881, published on 3 February 2023, reports that a total of nine cases of H5N1 were reported in China during 2010–14.^162^ These findings highlight the critical role of travellers as sentinels in detecting the spread of influenza and emphasize the importance of continuous surveillance of travellers to prevent respiratory infection outbreaks.

We observed that men were proportionately more often affected by respiratory symptoms and infections, which contrasts with other studies that have shown that the male sex is associated with a lower incidence of RTIs such as pneumonia and bronchitis.^163^ Age appears to have a lesser impact on respiratory infections in our study that were previously described. However, due to the large number of missing values for age and sex, we were not able to statistically verify these differences and the results should therefore be interpreted with care. Further studies are needed to estimate the sex differences in respiratory infections among travellers.

The number of articles published annually on respiratory infections in travellers has been increasing since the 2000s, indicating a growing interest among researchers (Figure 2). While this positive trend may be partially attributed to factors such as improved diagnosis, the identification of new viruses and an overall increase in awareness, the positive cyclical pattern observed, with up to four times more publications in the year following the four respiratory virus epidemics/pandemics of the twenty-first century, cannot be explained by these factors alone. This cyclical pattern suggests that travellers are significantly affected by these viruses and reflect the global spread of respiratory infectious diseases. In addition, maps created using reported cases in the literature provide further evidence of this overlap with official maps reporting the total number of cases (Appendix section 6). Since 1995, GeoSentinel, a network of travel and tropical medicine clinics, has identified geographic and temporal patterns of morbidity among travellers, immigrants and refugees. However, GeoSentinel relies on the reporting of illness from specified travel and tropical medicine clinics and sees only a small proportion of ill returning travellers, which makes it difficult to assess the true impact of respiratory infections in the global mass of travellers. Therefore, new studies are using apps to track travellers’ symptoms during travel to increase the accuracy of the data and to allow ‘bottom up’, real-time reporting of illness including respiratory illness. For instance, a pilot study that used an app called ITIT ‘Illness Tracking in Travellers’^164^ to capture travellers’ symptoms during their travel showed that 67% of the symptoms reported during travel were symptoms suggesting respiratory illness or infections.^165^

An important limitation of our study is that in many cases reference populations were not available, so that only absolute numbers could be calculated in the descriptive analysis. Another potential limitation is that most studies used a convenience sampling method because of the difficulty of following travellers throughout their journey. Therefore, most studies included participants who sought counselling before the trip or ill returned travellers. This may introduce bias into prevalence estimates, such as underestimation. In addition, studies reporting only ill travellers cannot be included in the meta-analysis to estimate prevalence. Overrepresentation of traveller groups, such as Hajj pilgrims, or imbalance in the number of reported cases from countries where testing is more readily available may also confound the results and partially explain why species identification is not possible in most cases. Finally, some high-risk groups such as VFRs, immunocompromised travellers or business travellers, and sociodemographic factors such as obesity are missing from our risk categories, so the results in this paper cannot be generalized to such subgroups.

## Conclusion

In summary, our systematic review and meta-analysis provide an estimate of the prevalences of respiratory symptoms and infections in travellers. In addition, we present the distribution of these infections across UN regions, specific groups of travellers and area of acquisition, as well as by sex. Respiratory symptoms and infections represent a significant burden for travellers and specific factors such as attendance at mass gatherings events increase the risk of infection. Travellers can act as sentinels and evaluations of traveller infections may be useful in identifying emerging respiratory infections of pandemic potential. Further studies are needed to better assess the true impact of these travel-acquired respiratory infections in terms of morbidity and quality-of-life impact. New digital tools such as mobile applications will allow researchers access to real-time data on travellers’ illness throughout their journey and allow for rapid response to emerging respiratory infections.

## Data sharing

The sources from which the data were obtained are listed in Appendix (section 2), and the absolute numbers used can be found there. The full data set is available upon request from the corresponding author (thibault.lovey@uzh.ch) after the article has been published and a data sharing request has been submitted.

## Author contributions

Thibault Lovey (Conceptualization-Equal, Data curation-Lead, Formal analysis-Lead, Investigation-Lead, Methodology-Equal, Visualization-Lead, Writing—original draft-Lead), Robin Hasler (Investigation-Equal, Writing—review & editing-Supporting), Philippe Gautret (Validation-Equal, Writing—review & editing-Equal), Patricia Schlagenhauf (Conceptualization-Equal, Investigation-Equal, Methodology-Equal, Supervision-Lead, Validation-Lead, Writing—review & editing-Lead).

**Conflict of interest**: None declared.

## Data availability statement

The full data set is available upon request from the corresponding author.

## Supplementary Material

Appendix_taad081
