# Supplementary material for: Travel-related respiratory symptoms and infections in travellers (2000–22): a systematic review and meta-analysis
Source: J Travel Med. 2023 Jun 13;30(5):taad081. doi: 10.1093/jtm/taad081 (PMC10481419; doi:10.1093/jtm/taad081)

Appendix (section 1). Search strings and number of articles retrieved per database.

Medline

(("Travel-Related Illness"[MeSH Terms] OR "Travel-Related Illness"[tiab] OR "Infections Travel-Associated"[tiab] OR "Travel Associated Infections"[tiab] OR "Travel-Associated Infection"[tiab] OR "travel related infections"[tiab] OR "travel related infections"[tiab] OR "Travel-Related Infection"[tiab] OR "travel related infectious diseases"[tiab] OR "Infectious Diseases Travel-Related"[tiab] OR "travel related infectious diseases"[tiab] OR "Travel-Related Infectious Disease"[tiab] OR "travel associated infectious diseases"[tiab] OR "travel associated infectious diseases"[tiab] OR "Travel-Associated Infectious Disease"[Title/Abstract]) AND ("english"[Language] OR "french"[Language] OR "spanish"[Language] OR "german"[Language]) AND ("Respiratory Tract Infections"[MeSH Terms] OR "infection respiratory tract"[tiab] OR "Respiratory Tract Infection"[tiab] OR "infections respiratory"[tiab] OR "infections respiratory tract"[tiab] OR "Respiratory Infections"[tiab] OR "Upper Respiratory Tract Infections"[tiab] OR "Upper Respiratory Infections"[tiab] OR "Upper Respiratory Tract Infection"[tiab] OR "infections upper respiratory tract"[tiab] OR "infections upper respiratory"[tiab] OR "respiratory infection upper"[Title/Abstract])) AND ((2000/1/1:2022/1/31[pdat]) AND (english[Filter] OR french[Filter] OR german[Filter] OR spanish[Filter])) **217**

Embase

('travel-related illness'/exp OR 'travel-related illness':ti,ab,kw OR 'infections travel-associated':ti,ab,kw OR 'travel associated infections':ti,ab,kw OR 'travel-associated infection':ti,ab,kw OR 'travel-related infections':ti,ab,kw OR 'infection travel-related':ti,ab,kw OR 'infections travel-related':ti,ab,kw OR 'travel related infections':ti,ab,kw OR 'travel-related infection':ti,ab,kw OR 'travel-related infectious diseases':ti,ab,kw OR 'disease travel-related infectious':ti,ab,kw OR 'diseases travel-related infectious':ti,ab,kw OR 'infectious disease travel-related':ti,ab,kw OR 'infectious diseases travel-related':ti,ab,kw OR 'travel related infectious diseases':ti,ab,kw OR 'travel-related infectious disease':ti,ab,kw OR 'travel-associated infectious diseases':ti,ab,kw OR 'disease travel-associated infectious':ti,ab,kw OR 'diseases travel-associated infectious':ti,ab,kw OR 'infectious disease travel-associated':ti,ab,kw OR 'infectious diseases travel-associated':ti,ab,kw OR 'travel associated infectious diseases':ti,ab,kw OR 'travel-associated infectious disease':ti,ab,kw OR 'travel-related sickness':ti,ab,kw OR 'sickness travel-related':ti,ab,kw OR 'sicknesses travel-related':ti,ab,kw OR 'travel related sickness':ti,ab,kw OR 'travel-related sicknesses':ti,ab,kw) AND ([english]/lim OR [german]/lim OR [french]/lim OR [spanish]/lim) AND ('respiratory tract infections'/exp OR 'infection, respiratory tract':ti,ab,kw OR 'respiratory tract infection':ti,ab,kw OR 'infections, respiratory':ti,ab,kw OR 'infections, respiratory tract':ti,ab,kw OR 'respiratory infections':ti,ab,kw OR 'upper respiratory tract infections':ti,ab,kw OR 'upper respiratory infections':ti,ab,kw OR 'upper respiratory tract infection':ti,ab,kw OR 'infections, upper respiratory tract':ti,ab,kw OR 'infections, upper respiratory':ti,ab,kw OR 'respiratory infection, upper':ti,ab,kw) AND ([english]/lim OR [french]/lim OR [german]/lim OR [spanish]/lim) AND [2000-2022]/py **376**

Web of science

(TS=(«travel-related illness» OR «infections travel-associated» OR «travel associated infections» OR «travel-associated infection» OR «travel-related infections» OR «infection travel-related» OR «infections travel-related» OR «travel related infections» OR «travel-related infection» OR «travel-related infectious diseases» OR «disease travel-related infectious» OR «diseases travel-related infectious» OR «infectious disease travel-related» OR «infectious diseases travel-related» OR «travel related infectious diseases» OR «travel-related infectious disease» OR «travel-associated infectious diseases» OR «disease travel-associated infectious» OR «diseases travel-associated infectious» OR «infectious disease travel-associated» OR «infectious diseases travel-associated» OR «travel associated infectious diseases» OR «travel-associated infectious disease» OR «travel-related sickness» OR «sickness travel-related» OR «sicknesses travel-related» OR «travel related sickness» OR «travel-related sicknesses») AND TS=(«respiratory tract infections» OR «infection, respiratory tract» OR «respiratory tract infection» OR «infections, respiratory» OR «infections, respiratory tract» OR «respiratory infections» OR «upper respiratory tract infections» OR «upper respiratory infections» OR «upper respiratory tract infection» OR «infections, upper respiratory tract» OR «infections, upper respiratory» OR «respiratory infection, upper»)) AND (PY==("2000" OR "2001" OR "2002" OR "2003" OR "2004" OR "2005" OR "2022" OR "2021" OR "2020" OR "2019" OR "2018" OR "2017" OR "2016" OR "2015" OR "2014" OR "2013" OR "2012" OR "2011" OR "2010") AND LA==("ENGLISH" OR "SPANISH" OR "FRENCH" OR "GERMAN")) **1265**

Scopus

TITLE-ABS-KEY ("Travel-Related Illness" OR "Infections Travel-Associated" OR "Travel Associated Infections" OR "Travel-Associated Infection" OR "Travel-Related Infections" OR "Infection Travel-Related" OR "Infections Travel-Related" OR "Travel Related Infections" OR "Travel-Related Infection" OR "Travel-Related Infectious Diseases" OR "Disease Travel-Related Infectious" OR "Diseases Travel-Related Infectious" OR "Infectious Disease Travel-Related" OR "Infectious Diseases Travel-Related" OR "Travel Related Infectious Diseases" OR "Travel-Related Infectious Disease" OR "Travel-Associated Infectious Diseases" OR "Disease Travel-Associated Infectious" OR "Diseases Travel-Associated Infectious" OR "Infectious Disease Travel-Associated" OR "Infectious Diseases Travel-Associated" OR "Travel Associated Infectious Diseases" OR "Travel-Associated Infectious Disease" OR "Travel-Related Sickness" OR "Sickness Travel-Related" OR "Sicknesses Travel-Related" OR "Travel Related Sickness" OR "Travel-Related Sicknesses") AND TITLE-ABS-KEY ("Respiratory Tract Infections" OR "Infection, Respiratory Tract" OR "Respiratory Tract Infection" OR "Infections, Respiratory" OR "Infections, Respiratory Tract" OR "Respiratory Infections" OR "Upper Respiratory Tract Infections" OR "Upper Respiratory Infections" OR "Upper Respiratory Tract Infection" OR "Infections, Upper Respiratory Tract" OR "Infections, Upper Respiratory" OR "Respiratory Infection, Upper") AND (EXCLUDE (PUBYEAR , 1999) OR EXCLUDE (PUBYEAR , 1998) OR EXCLUDE (PUBYEAR , 1991)) AND (LIMIT-TO (LANGUAGE , "English") OR LIMIT-TO (LANGUAGE , "French") OR LIMIT-TO (LANGUAGE , "German") OR LIMIT-TO (LANGUAGE , "Spanish")) **74**

Cochrane
MeSH descriptor: [Travel-Related Illness] explode all trees AND MeSH descriptor: [Respiratory Tract Infections] explode all trees **3**

Science direct
("Travel-Related Illness" OR "Travel-Related Infection" OR "Travel-Related Infectious Diseases" OR "Travel-Related Sickness") AND ("Respiratory Tract Infection" OR "Respiratory Infections" OR "Upper Respiratory Infections" OR "Upper Respiratory Tract) **6**

MedRxiv/BioRxiv
for abstract or title "Respiratory Tract Infections" (match all words) and full text or abstract or title "Travel-Related Illness" (match whole all) **bioRxiv 16 / medRxiv 73**

SSRN
respiratory infection AND travel **10 (**Title, Abstract & Keywords)

IEEE Xplore
("Full Text & Metadata":travel-related illness) AND ("Full Text & Metadata":respiratory tract infection) **2**

Exclusion:
Older than 2000
Not in English, French, Spanish, German

Appendix (section 2). The complete numbered, alphabetically sorted literature list of articles included in the systematic review and the number of reported cases with respiratory symptoms and/or respiratory cases. The reference population, if any, and stratification by age group and sex are also provided. In green and blue are the studies included in the meta-analysis for prevalence of respiratory symptoms and respiratory cases, respectively.

Study ID	Authors	Publication Year	Title	Journal	Volume	Issue	Pages	Study Design	Starting date	Ending date	Country of detection	JBI Critical Score (%)	Respiratory Symptoms										Respiratory Cases Reported									
													Total cases (n)	Age Distribution					Gender Distribution			Total population (N)	Total cases (n)	Age Distribution					Gender Distribution			Total population (N)
														Child/Young	Adult	Middle Age Adult	Senior Adult	Unknown Age	Female	Male	Unknown Gender			Child/Young	Adult	Middle Age Adult	Senior Adult	Unknown Age	Female	Male	Unknown Gender	
1	Aberle, J. H., Popow-Kraupp, T. et al.	2015	Influenza A and B Viruses but Not MERS-CoV in Hajj Pilgrims, Austria, 2014	Emerging Infectious Diseases	21	4	726-727	Case series	2014-10-02	2014-10-27	Austria	70	7	NA	NA	6	1	NA	2	5	NA	7	7	NA	NA	6	1	NA	2	5	NA	7
2	Abroug, F., Slim, A. et al.	2014	Family Cluster of Middle East Respiratory Syndrome Coronavirus Infections, Tunisia, 2013	Emerging Infectious Diseases	20	9	1527-1530	Case series	2013-03-20	2013-05-18	Tunisia	95	3	NA	2	NA	1	NA	1	2	NA	3	3	NA	2	NA	1	NA	1	2	NA	3
3	Aguilera, J. F., Perrocheau, A. et al.	2002	Outbreak of Serogroup W135 Meningococcal Disease after the Hajj Pilgrimage, Europe, 2000	Emerging Infectious Diseases	8	8	761-767	Cross-sectional studies	2000-03-18	2000-07-31	Several	62	NA	NA	NA	NA	NA	NA	NA	NA	NA	NA	12	NA	NA	NA	NA	12	8	4	NA	90
4	Ahmad Abou Tayoun, Tom Loney et al.	2020	Multiple early introductions of SARS-CoV-2 into a global travel hub in the Middle East	Scientific Reports	10	1	NA	Case series	2020-01-29	2020-03-18	United Arab Emirates	100	NA	NA	NA	NA	NA	NA	NA	NA	NA	NA	40	2	NA	NA	2	36	17	32	NA	49
5	Ahmad, N., Ali, N. F. M. et al.	2021	Clinical Characteristics of Patients with Coronavirus Disease 2019 (COVID-19) in a Teaching Hospital in Malaysia	Journal Of Infection In Developing Countries	15	8	1059-1065	Case series	2020-03-30	2020-04-22	Malaysia	94	5	NA	NA	NA	NA	5	NA	NA	5	19	19	NA	NA	NA	NA	19	NA	NA	19	147
6	Ake, O. W., Elimian, K. O. et al.	2021	Epidemiological comparison of the first and second waves of the COVID-19 pandemic in Nigeria, February 2020-April 2021	BMJ Global Health	6	11	NA	Cross-sectional studies	2020-02-27	2021-04-03	Nigeria	94	NA	NA	NA	NA	NA	NA	NA	NA	NA	NA	3396	NA	NA	NA	NA	3396	NA	NA	3396	71829
7	Al-Abdallat, M. M., Rha, B. et al.	2017	Acute respiratory infections among returning Hajj pilgrims—Jordan, 2014	Journal Of Clinical Virology	89	NA	34-37	Cross-sectional studies	2014-10-08	2014-10-23	Jordan	94	NA	NA	NA	NA	NA	NA	NA	NA	NA	125	73	NA	NA	NA	NA	73	NA	NA	73	125
8	Al-Asmary, S., Al-Shehri, A. S. et al.	2006	Acute respiratory tract infections among Hajj medical mission personnel, Saudi Arabia	International Journal Of Infectious Diseases	11	3	268-272	Cross-sectional studies	2005-01-18	2005-01-23	Saudi Arabia	56	NA	NA	NA	NA	NA	NA	NA	NA	NA	NA	64	NA	NA	NA	NA	64	NA	NA	64	250
9	Al-Jasser, F. S., Kabbash, I. A. et al.	2013	Patterns of diseases and preventive measures among domestic hajjis from Central, Saudi Arabia	Saudi Medical Journal	33	8	879-886	Cross-sectional studies	2009-11-01	2009-12-01	Saudi Arabia	69	800	NA	NA	NA	NA	800	317	483	NA	1507	NA	NA	NA	NA	NA	NA	NA	NA	NA	NA
10	Al-Rifai, R. H., Acuna, J. et al.	2021	Epidemiological characterization of symptomatic and asymptomatic COVID-19 cases and positivity in subsequent RT-PCR tests in the United Arab Emirates	PLOS One	16	2	NA	Cohort studies	2020-02-28	2020-04-08	United Arab Emirates	86	112	NA	NA	NA	NA	112	NA	NA	112	187	187	NA	NA	NA	NA	187	NA	NA	187	706
11	Al-Tawfiq, J. A., Sattar, A. et al.	2020	Incidence of COVID-19 among returning travelers in quarantine facilities: A longitudinal study and lessons learned	Travel Medicine & Infectious Disease	38	NA	NA	Case series	2020-03-14	2020-06-07	Saudi Arabia	81	NA	NA	NA	NA	NA	NA	NA	NA	NA	NA	23	5	NA	NA	NA	41	NA	NA	23	1928
12	Alahmari, A. A., Khan, A. A. et al.	2021	Epidemiological and clinical features of COVID-19 patients in Saudi Arabia	Journal Of Infection & Public Health	14	4	437-443	Cross-sectional studies	2020-03-01	2020-06-20	Saudi Arabia	100	NA	NA	NA	NA	NA	NA	NA	NA	NA	NA	11307	NA	NA	NA	NA	11307	NA	NA	11307	25073
13	Alahmari, A. A., Khan, A. A. et al.	2020	Tracking Australian Hajj Pilgrims' Health Behavior before, during and after Hajj, and the Effective Use of Preventive Measures in Reducing Hajj-Related Illness: A Cohort Study	Pharmacy	8	2	NA	Cohort studies	2015-08-01	2015-12-01	Australia	68	248	NA	NA	NA	NA	248	NA	NA	248	391	NA	NA	NA	NA	NA	NA	NA	NA	NA	NA
14	Alasmari, A. K., Edwards, P. J. et al.	2020	Use of face masks and other personal preventive measures by Hajj pilgrims and their impact on health problems during the Hajj	Journal Of Travel Medicine	27	8	NA	Cross-sectional studies	2017-09-04	2017-09-30	Saudi Arabia	81	1150	NA	NA	NA	NA	1150	NA	NA	1150	2973	NA	NA	NA	NA	NA	NA	NA	NA	NA	NA
15	Alborzi, A., Aelami, M. H. et al.	2009	Viral Etiology of Acute Respiratory Infections Among Iranian Hajj Pilgrims, 2006	Journal Of Travel Medicine	16	4	239-242	Cross-sectional studies	2006-12-01	2007-01-31	Iran (Islamic Republic of)	62	NA	NA	NA	NA	NA	NA	NA	NA	NA	255	83	NA	NA	NA	NA	83	NA	NA	83	255
16	Aldridge, R. W., Zenner, D. et al.	2016	Prevalence of and risk factors for active tuberculosis in migrants screened before entry to the UK: a population-based cross-sectional study	Lancet Infectious Diseases	16	8	962-970	Cross-sectional studies	2005-10-01	2013-12-31	UK	75	NA	NA	NA	NA	NA	NA	NA	NA	NA	NA	439	NA	NA	NA	NA	439	NA	NA	439	476455
17	Alfelali, M., Haworth, E. A. et al.	2021	Facemask versus No Facemask in Preventing Viral Respiratory Infections During Hajj: A Cluster Randomised Open Label Trial	SSRN Electronic Journal	NA	NA	NA	RCT	2013-01-01	2015-12-31	Saudi Arabia	62	676	NA	NA	NA	NA	676	NA	NA	676	6338	277	NA	NA	NA	NA	277	NA	NA	277	650
18	Almazroa, M. A., Memish, Z. A. et al.	2010	Pandemic influenza A (H1N1) in Saudi Arabia: description of the first one hundred cases	Annals Of Saudi Medicine	30	1	11-14	Cross-sectional studies	2009-06-01	2009-09-03	Saudi Arabia	62	NA	NA	NA	NA	NA	NA	NA	NA	NA	NA	47	NA	NA	NA	NA	47	NA	NA	47	100
19	Alon, D., Shitrit, P. et al.	2010	Risk Behaviors and Spectrum of Diseases Among Elderly Travelers: A Comparison of Younger and Older Adults	Journal Of Travel Medicine	17	4	250-255	Cohort studies	2008-01-01	2008-06-01	Israel	68	21	NA	12	NA	9	NA	NA	NA	21	394	NA	NA	NA	NA	NA	NA	NA	NA	NA	NA

Study ID	Authors	Publication Year	Title	Journal	Volume	Issue	Pages	Study Design	Starting date	Ending date	Country of detection	JBI Critical Score (%)	Respiratory Symptoms										Respiratory Cases Reported									
													Total cases (n)	Age Distribution: Child/Young	Adult	Middle Age Adult	Senior Adult	Unknown Age	Gender Distribution: Female	Male	Unknown Gender	Total population (N)	Total cases (n)	Age Distribution: Child/Young	Adult	Middle Age Adult	Senior Adult	Unknown Age	Gender Distribution: Female	Male	Unknown Gender	Total population (N)
20	Alqahtani, A. S., Bindhim, N. F. et al.	2016	Pilot use of a novel smartphone application to track traveller health behaviour and collect infectious disease data during a mass gathering: Hajj pilgrimage 2014	Journal Of Epidemiology & Global Health	6	3	147-55	Cohort studies	2014-09-05	2014-10-30	Australia	77	NA	NA	NA	NA	NA	NA	NA	NA	NA	25	NA	NA	NA	NA	NA	NA	NA	NA	NA	NA
21	Alzahrani, A. G., Choudhry, A. J. et al.	2011	Pattern of diseases among visitors to Mina health centers during the Hajj season, 1429 H (2008 G)	Journal Of Infection & Public Health	5	1	22-34	Cross-sectional studies	2008-12-06	2008-12-10	Saudi Arabia	100	NA	NA	NA	NA	NA	NA	NA	NA	NA	NA	2424	NA	NA	NA	NA	2424	NA	NA	2424	3696
22	Alzurba, F., Saab, B. et al.	2007	Medical Problems Encountered Among Travelers in Bahrain International Airport Clinic	Journal Of Travel Medicine	14	1	37-41	Cross-sectional studies	2004-01-01	2004-12-31	Bahrain	56	NA	NA	NA	NA	NA	NA	NA	NA	NA	NA	816	NA	NA	NA	NA	816	NA	NA	816	3350
23	Andrews, M. A., Areekal, B. et al.	2020	First confirmed case of COVID-19 infection in India: A case report	Indian Journal Of Medical Research	151	5	490-492	Case report	2020-01-27	2020-01-27	India	81	1	NA	1	NA	NA	NA	1	NA	NA	1	1	NA	NA	NA	NA	1	1	NA	NA	1
24	Angelin, M., Evengrd, B. et al.	2015	Illness and risk behaviour in health care students studying abroad	Medical Education	49	7	684-691	Prevalence studies	2010-04-01	2014-01-31	Sweden	89	NA	NA	NA	NA	NA	NA	NA	NA	NA	NA	86	NA	NA	NA	NA	86	NA	NA	86	335
25	Angelo, K. M., Haulman, N. J. et al.	2018	Illness among US resident student travellers after return to the USA: a GeoSentinel analysis, 2007–17	Journal Of Travel Medicine	25	1	NA	Cross-sectional studies	2007-01-01	2017-12-31	United States of America	62	NA	NA	NA	NA	NA	NA	NA	NA	NA	581	58	NA	NA	NA	NA	58	NA	NA	58	581
26	Annan, A., Owusu, M. et al.	2015	High prevalence of common respiratory viruses and no evidence of Middle East Respiratory Syndrome Coronavirus in Hajj pilgrims returning to Ghana, 2013	Tropical Medicine & International Health	20	6	807-812	Cross-sectional studies	2013-11-01	2013-11-30	Ghana	75	651	NA	NA	NA	NA	651	NA	NA	651	839	179	NA	NA	NA	NA	179	NA	NA	179	839
27	Ansart, S., Pajot, O. et al.	2006	Pneumonia among Travelers Returning from Abroad	Journal Of Travel Medicine	11	2	87-91	Cross-sectional studies	2001-08-01	2002-09-30	France	62	NA	NA	NA	NA	NA	NA	NA	NA	NA	NA	17	NA	NA	NA	NA	17	8	9	NA	17
28	Ansart, S., Perez, L. et al.	2005	Illnesses in Travelers Returning from the Tropics: A Prospective Study of 622 Patients	Journal Of Travel Medicine	12	6	312-318	Cross-sectional studies	2002-11-01	2003-05-01	France	88	NA	NA	NA	NA	NA	NA	NA	NA	NA	622	90	NA	NA	NA	NA	90	NA	NA	90	622
29	Aoun, O., Roqueplo, C. et al.	2014	Spectrum and impact of health problems during deployment: A prospective, multicenter study of French soldiers operating in Afghanistan, Lebanon and Côte d'Ivoire	Travel Medicine & Infectious Disease	12	4	378-384	Cohort studies	2008-06-01	2008-10-01	Several	64	NA	NA	NA	NA	NA	NA	NA	NA	NA	NA	428	NA	NA	NA	NA	428	NA	NA	428	4349
30	Arashiro, T., Nakamura, S. et al.	2020	SARS-CoV-2 and Legionella co-infection in a person returning from a Nile cruise	Journal Of Travel Medicine	27	3	NA	Case report	2020-02-26	2020-03-04	Japan	94	1	NA	NA	NA	1	NA	NA	1	NA	1	1	NA	NA	NA	1	NA	NA	1	NA	1
31	Arce Arnez, A., Iigo Martnez, J. et al.	2009	Tuberculosis e inmigracin en un rea sanitaria de Madrid. Situacin epidemiolgica y evolucin en la dcada 1994-2003	Medicina Clínica	125	6	210-212	Cross-sectional studies	1994-01-01	2003-12-31	Spain	81	NA	NA	NA	NA	NA	NA	NA	NA	NA	NA	269	NA	NA	NA	NA	269	NA	NA	269	2211
32	Arima, Y., Kutsuna, S. et al.	2020	Severe Acute Respiratory Syndrome Coronavirus 2 Infection among Returnees to Japan from Wuhan, China, 2020	Emerging Infectious Diseases	26	7	1596-1600	Cross-sectional studies	2020-01-29	2020-01-31	Japan	81	8	NA	NA	8	NA	NA	NA	NA	12	12	12	NA	NA	NA	1	7	NA	NA	NA	566
33	Asghar, A. H., Ashshi, A. M. et al.	2011	Profile of bacterial pneumonia during Hajj	Indian Journal Of Medical Research	133	NA	510-513	Cross-sectional studies	2005-01-01	2005-01-20	Saudi Arabia	56	110	NA	NA	NA	NA	110	NA	NA	110	141	141	NA	2	5	85	49	33	43	65	141
34	Ashshi, A., Azhar, E. et al.	2014	Demographic distribution and transmission potential of influenza A and 2009 pandemic influenza A H1N1 in pilgrims	Journal Of Infection In Developing Countries	8	9	1169-1175	Cross-sectional studies	2010-11-14	2010-11-19	Saudi Arabia	56	NA	NA	NA	NA	NA	NA	NA	NA	NA	1600	120	NA	NA	NA	NA	120	49	71	NA	1600
35	Askling, H. H., Lesko, B. et al.	2009	Serologic Analysis of Returned Travelers with Fever, Sweden	Emerging Infectious Diseases	15	11	1805-1808	Cross-sectional studies	2005-03-14	2008-03-14	Sweden	88	NA	NA	NA	NA	NA	NA	NA	NA	NA	NA	82	NA	NA	NA	NA	82	NA	NA	82	1432
36	Assiri, A. M., Asiri, S. I. et al.	2018	Burden of influenza-related severe acute respiratory infections during Hajj season 1438 (2017). Lessons and future directions	Saudi Medical Journal	39	5	524-525	Cross-sectional studies	2017-08-30	2017-09-04	Saudi Arabia	75	NA	NA	NA	NA	NA	NA	NA	NA	NA	NA	289	NA	NA	103	175	11	NA	NA	289	909
37	Assiri, A., Al-Tawfiq, J. A. et al.	2013	Epidemiological, demographic, and clinical characteristics of 47 cases of Middle East respiratory syndrome coronavirus disease from Saudi Arabia: a descriptive study	Lancet Infectious Diseases	13	9	752-761	Case series	2012-09-01	2013-06-15	Saudi Arabia	90	NA	NA	NA	NA	NA	NA	NA	NA	NA	NA	4	NA	NA	NA	NA	4	NA	NA	4	47
38	Avni, C., Stienlauf, S. et al.	2018	Region-Specific, Life-Threatening Diseases among International Travelers from Israel, 2004–2015	Emerging Infectious Diseases	24	4	790-793	Cross-sectional studies	2004-01-01	2015-01-01	Israel	88	NA	NA	NA	NA	NA	NA	NA	NA	NA	NA	86	NA	NA	NA	NA	86	NA	NA	86	722
39	Bae, S. H., Shin, H. et al.	2020	Asymptomatic Transmission of SARS-CoV-2 on Evacuation Flight	Emerging Infectious Diseases	26	11	2705-2708	Cross-sectional studies	2020-03-31	2020-04-15	Several	81	NA	NA	NA	NA	NA	NA	NA	NA	NA	NA	17	NA	NA	NA	NA	17	2	4	11	310

Study ID	Authors	Publication Year	Title	Journal	Volume	Issue	Pages	Study Design	Starting date	Ending date	Country of detection	JBI Critical Score (%)	Respiratory Symptoms										Respiratory Cases Reported									
													Total cases (n)	Age Distribution					Gender Distribution			Total population (N)	Total cases (n)	Age Distribution					Gender Distribution			Total population (N)
														Child/Young	Adult	Middle Age Adult	Senior Adult	Unknown Age	Female	Male	Unknown Gender			Child/Young	Adult	Middle Age Adult	Senior Adult	Unknown Age	Female	Male	Unknown Gender	
40	Baharoon, S., Al-Jahdali, H. et al.	2008	Severe sepsis and septic shock at the Hajj: Etiologies and outcomes	Travel Medicine & Infectious Disease	7	4	247-252	Cross-sectional studies	2004-01-08	2004-02-21	Saudi Arabia	81	NA	NA	NA	NA	NA	NA	NA	NA	NA	NA	23	NA	NA	NA	NA	23	NA	NA	23	42
41	Bajema, K. L., Oster, A. M. et al.	2020	Persons Evaluated for 2019 Novel Coronavirus — United States, January 2020	Morbidity & Mortality Weekly Report	69	6	166-170	Cross-sectional studies	2020-01-01	2020-01-31	United States of America	81	9	NA	NA	NA	NA	9	NA	NA	9	148	9	NA	NA	NA	NA	NA	NA	NA	NA	148
42	Baker, M. G., Thornley, C. N. et al.	2010	Transmission of pandemic A/H1N1 2009 influenza on passenger aircraft: retrospective cohort study	BMJ	340	NA	c2424-c2424	Cross-sectional studies	2009-04-25	2009-05-04	New Zealand	62	NA	NA	NA	NA	NA	NA	NA	NA	NA	NA	12	NA	NA	NA	NA	12	NA	NA	12	46
43	Balaban, V., Stauffer, W. M. et al.	2012	Protective Practices and Respiratory Illness Among US Travelers to the 2009 Hajj	Journal Of Travel Medicine	19	3	163-168	Prevalence studies	2009-10-01	2010-02-08	United States of America	89	76	NA	NA	NA	NA	76	NA	NA	76	221	NA	NA	NA	NA	NA	NA	NA	NA	NA	NA
44	Balkhy, H. H., Memish, Z. A. et al.	2006	Influenza a Common Viral Infection among Hajj Pilgrims: Time for Routine Surveillance and Vaccination	Journal Of Travel Medicine	11	2	82-86	Cross-sectional studies	2003-02-09	2003-02-14	Saudi Arabia	88	NA	NA	NA	NA	NA	NA	NA	NA	NA	NA	54	NA	NA	NA	NA	54	22	32	NA	500
45	Barasheed, O., Almasri, N. et al.	2014	Pilot Randomised Controlled Trial to Test Effectiveness of Facemasks in Preventing Influenza-like Illness Transmission among Australian Hajj Pilgrims in 2011	Infectious Disorders - Drug Targets	14	2	110-116	RCT	2010-11-04	2010-11-10	Saudi Arabia	65	NA	NA	NA	NA	NA	NA	NA	NA	NA	164	47	NA	NA	NA	NA	47	NA	NA	47	164
46	Barbero González, A., Álvarez De Toledo Saavedra, F. et al.	2013	Management of vaccinations and prophylaxis of international travellers from community pharmacy (vintaf study)	Atención Primaria	32	5	276-281	Cross-sectional studies	2000-06-10	2000-10-10	Spain	88	14	NA	NA	NA	NA	14	NA	NA	14	437	NA	NA	NA	NA	NA	NA	NA	NA	NA	NA
47	Barbosa, F., Barnett, E. D. et al.	2017	Bordetella pertussis infections in travelers: data from the GeoSentinel global network	Journal Of Travel Medicine	24	3	NA	Cross-sectional studies	1999-01-01	2015-07-01	Several	81	NA	NA	NA	NA	NA	NA	NA	NA	NA	NA	74	6	NA	NA	9	58	38	36	NA	74
48	Barnett, E. D., Weld, L. H. et al.	2012	Spectrum of Illness in International Migrants Seen at GeoSentinel Clinics in 19972009, Part 1: US-Bound Migrants Evaluated by Comprehensive Protocol-Based Health Assessment	Clinical Infectious Diseases	56	7	913-924	Cross-sectional studies	1997-03-01	2012-11-30	United States	69	NA	NA	NA	NA	NA	NA	NA	NA	NA	NA	3572	NA	NA	NA	NA	3572	NA	NA	3572	7792
49	Bastola, A., Sah, R. et al.	2020	The first 2019 novel coronavirus case in Nepal	Lancet Infectious Diseases	20	3	279-280	Case report	2020-01-13	2020-01-31	Nepal	100	1	NA	1	NA	NA	NA	NA	1	NA	1	1	NA	NA	NA	NA	1	NA	1	NA	1
50	Belderok, S. M., Rimmelzwaan, G. F. et al.	2013	Effect of Travel on Influenza Epidemiology	Emerging Infectious Diseases	19	6	925-931	Cross-sectional studies	2006-10-01	2007-10-01	Netherlands	100	NA	NA	NA	NA	NA	NA	NA	NA	NA	86	86	NA	22	13	5	46	55	31	NA	1190
51	Benkouiten, S., Charrel, R. et al.	2013	Circulation of Respiratory Viruses Among Pilgrims During the 2012 Hajj Pilgrimage	Clinical Infectious Diseases	57	7	1000-992	Cross-sectional studies	2012-08-01	2012-11-30	France	88	124	NA	NA	NA	NA	124	NA	NA	124	137	27	NA	NA	NA	NA	27	NA	NA	27	70
52	Benkouiten, S., Charrel, R. et al.	2014	Respiratory Viruses and Bacteria among Pilgrims during the 2013 Hajj	Emerging Infectious Diseases	20	11	1821-1827	Cross-sectional studies	2013-09-15	2013-10-24	France	88	117	NA	NA	NA	NA	117	NA	NA	117	129	36	NA	NA	NA	NA	36	NA	NA	36	121
53	Bermingham, A., Ch, M. A. et al.	2012	Severe respiratory illness caused by a novel coronavirus, in a patient transferred to the United Kingdom from the Middle East, September 2012	Eurosurveillance	17	40	6-10	Case report	2012-09-14	2012-10-02	UK	81	1	NA	NA	1	NA	NA	NA	1	1	1	1	NA	NA	1	NA	NA	NA	1	NA	1
54	Bernard Stoecklin, S., Rolland, P. et al.	2020	First cases of coronavirus disease 2019 (COVID-19) in France: surveillance, investigations and control measures, January 2020	Eurosurveillance	25	6	NA	Case series	2020-01-10	2020-01-24	France	80	3	NA	2	1	NA	NA	1	2	NA	NA	3	NA	2	1	NA	NA	1	2	NA	3
55	Bertollini, R., Chemaitelly, H. et al.	2021	Associations of Vaccination and of Prior Infection With Positive PCR Test Results for SARS-CoV-2 in Airline Passengers Arriving in Qatar	JAMA	326	2	184-188	Cohort studies	2021-02-18	2021-04-26	Qatar	91	NA	NA	NA	NA	NA	NA	NA	NA	NA	NA	831	NA	NA	NA	NA	831	NA	NA	831	34741
56	Bhari, D., Pey, P.	2018	Health problems while working as a volunteer or humanitarian aid worker in post-earthquake Nepal	Journal Of Nepal Medical Association	56	211	691-695	Cross-sectional studies	2015-05-01	2015-11-30	Nepal	75	6	NA	NA	NA	NA	6	NA	NA	6	95	NA	NA	NA	NA	NA	NA	NA	NA	NA	NA
57	Bhari, S., Bhargava, A. et al.	2020	Clinical Profile of Covid-19 Infected Patients Admitted in a Tertiary Care Hospital in North India	Journal Of The Association Of Physicians Of India	68	5	13-17	Case series	NA	2020-03-03	India	80	11	1	9	2	3	NA	5	10	NA	15	15	1	9	2	3	NA	5	10	NA	15
58	Bialek, S. R., Allen, D. et al.	2014	First Confirmed Cases of Middle East Respiratory Syndrome Coronavirus (MERS-CoV) Infection in the United States, Updated Information on the Epidemiology of MERS-CoV Infection, and Guidance for the Public, Clinicians, and Public Health Authorities - May 2014	Morbidity & Mortality Weekly Report	63	19	431-436	Case series	2012-09-01	2014-04-12	United States of America	65	NA	NA	NA	NA	NA	NA	NA	NA	NA	NA	20	NA	NA	NA	NA	20	NA	NA	20	NA

Study ID	Authors	Publication Year	Title	Journal	Volume	Issue	Pages	Study Design	Starting date	Ending date	Country of detection	JBI Critical Score (%)	Respiratory Symptoms										Respiratory Cases Reported									
													Total cases (n)	Age Distribution					Gender Distribution			Total population (N)	Total cases (n)	Age Distribution					Gender Distribution			Total population (N)
														Child/Young	Adult	Middle Age Adult	Senior Adult	Unknown Age	Female	Male	Unknown Gender			Child/Young	Adult	Middle Age Adult	Senior Adult	Unknown Age	Female	Male	Unknown Gender	
59	Binsaeed, A. A.	2010	Characteristics of pandemic influenza A (H1N1) infection in patients presenting to a university hospital in Riyadh, Saudi Arabia	Annals Of Saudi Medicine	30	1	59-62	Cross-sectional studies	2009-09-01	2009-09-12	Saudi Arabia	62	NA	NA	NA	NA	NA	NA	NA	NA	NA	NA	23	NA	NA	NA	NA	23	NA	NA	23	117
60	Birnie, E., Savelkoel, J. et al.	2019	Melioidosis in travelers: An analysis of Dutch melioidosis registry data 19852018	Travel Medicine & Infectious Disease	32	NA	101461	Cross-sectional studies	1985-01-01	2018-12-31	Netherlands	88	NA	NA	NA	NA	NA	NA	NA	NA	NA	NA	33	NA	NA	NA	NA	33	10	23	NA	33
61	Blyth, C. C., Foo, H. et al.	2010	Influenza Outbreaks during World Youth Day 2008 Mass Gathering	Emerging Infectious Diseases	16	5	809-815	Cross-sectional studies	2008-07-01	2008-07-31	Australia	62	NA	NA	NA	NA	NA	NA	NA	NA	NA	NA	100	NA	NA	NA	NA	100	NA	NA	100	227
62	Boehmer, M. M., Buchholz, U. et al.	2020	Investigation of a COVID-19 outbreak in Germany resulting from a single travel-associated primary case: a case series	Lancet Infectious Diseases	20	8	920-928	Case series	2020-01-27	2020-02-11	Germany	100	1	NA	NA	NA	NA	1	NA	1	NA	1	1	NA	NA	NA	NA	1	NA	1	NA	1
63	Boggild, A. K., Castelli, F. et al.	2010	Vaccine preventable diseases in returned international travelers: Results from the GeoSentinel Surveillance Network	Vaccine	28	46	7389-7395	Cohort studies	1997-03-01	2007-12-01	Several	68	NA	NA	NA	NA	NA	NA	NA	NA	NA	NA	139	NA	NA	NA	NA	139	NA	NA	139	37542
64	Boggild, A. K., Esposito, D. H. et al.	2016	Differential Diagnosis of Illness in Travelers Arriving From Sierra Leone, Liberia, or Guinea: A Cross-sectional Study From the GeoSentinel Surveillance Network	Annals Of Internal Medicine	162	11	757-764	Cross-sectional studies	2009-09-01	2014-08-01	Several	94	NA	NA	NA	NA	NA	NA	NA	NA	NA	NA	54	NA	NA	NA	NA	54	NA	NA	54	805
65	Boggild, A. K., Geduld, J. et al.	2014	Travel-acquired infections and illnesses in Canadians: surveillance report from CanTravNet surveillance data, 2009-2011	Open Medicine: A Peer-Reviewed, Independent, Open-Access Journal	8	1	32-e20	Cross-sectional studies	2009-09-01	2011-09-30	Several	100	NA	NA	NA	NA	NA	NA	NA	NA	NA	NA	210	NA	NA	NA	NA	210	NA	NA	210	3943
66	Booth, C. M.	2003	Clinical Features and Short-term Outcomes of 144 Patients With SARS in the Greater Toronto Area	JAMA	289	21	2801	Cross-sectional studies	2003-03-07	2003-04-10	Canada	88	NA	NA	NA	NA	NA	NA	NA	NA	NA	NA	3	NA	NA	NA	NA	3	NA	NA	3	144
67	Botelho-Nevers, E., Gautret, P. et al.	2010	Travel-Related Influenza A/H1N1 Infection at a Rock Festival in Hungary: One Virus May Hide Another One	Journal Of Travel Medicine	17	3	197-198	Case report	2009-08-11	2009-08-18	Several	94	1	NA	1	NA	NA	NA	NA	1	NA	1	15	NA	1	NA	NA	14	NA	1	14	15
68	Bottieau, E., Clerinx, J. et al.	2006	Etiology and Outcome of Fever After a Stay in the Tropics	Archives Of Internal Medicine	166	15	1642	Cross-sectional studies	2000-01-01	2005-12-31	Netherlands	88	609	NA	NA	NA	NA	609	NA	NA	609	1842	315	NA	NA	NA	NA	315	NA	NA	315	1842
69	Brosh-Nissimov, T., Grupel, D. et al.	2019	Case Report: Imported Melioidosis from Goa, India to Israel, 2018	American Journal Of Tropical Medicine & Hygiene	101	3	580-584	Case report	2017-08-01	2017-10-01	France	94	1	NA	1	NA	NA	NA	NA	1	NA	NA	1	NA	1	NA	NA	NA	NA	1	NA	1
70	Brotherton, J. M. L., Delpech, V. C. et al.	2002	A large outbreak of influenza A and B on a cruise ship causing widespread morbidity	Epidemiology & Infection	130	2	263-271	Cross-sectional studies	2000-09-01	2000-09-30	Australia	100	NA	NA	NA	NA	NA	NA	NA	NA	NA	1159	203	NA	NA	NA	NA	203	NA	NA	203	1159
71	Buchholz, U., Altmann, D. et al.	2020	Differential Seasonality of Legionnaires' Disease by Exposure Category	International Journal Of Environmental Research & Public Health	17	9	NA	Cross-sectional studies	2005-01-01	2015-12-31	Germany	62	NA	NA	NA	NA	NA	NA	NA	NA	NA	NA	1477	NA	NA	NA	NA	1477	NA	NA	1477	7351
72	Buitrago, M. J., Bernalmartnez, L. et al.	2010	Histoplasmosis and Paracoccidioidomycosis in a NonEndemic Area: A Review of Cases and Diagnosis	Journal Of Travel Medicine	18	1	26-33	Cross-sectional studies	2006-01-01	2009-12-31	Spain	75	NA	NA	NA	NA	NA	NA	NA	NA	NA	NA	45	NA	NA	NA	NA	45	18	27	NA	45
73	Burrell, A. J. C., Pellegrini, B. et al.	2020	Outcomes for patients with COVID-19 admitted to Australian intensive care units during the first four months of the pandemic	Medical Journal Of Australia	214	1	23-30	Cross-sectional studies	2020-02-27	2020-06-30	Australia	100	NA	NA	NA	NA	NA	NA	NA	NA	NA	NA	114	NA	NA	NA	NA	144	NA	NA	144	204
74	Cabada, M. M., Maldonado, F. et al.	2008	Self-reported health problems among travelers visiting Cuzco: A Peruvian Airport survey	Travel Medicine & Infectious Disease	7	1	25-29	Prevalence studies	2002-08-01	2002-11-01	Peru	83	617	NA	NA	NA	NA	NA	NA	NA	NA	5988	NA	NA	NA	NA	NA	NA	NA	NA	NA	NA
75	Calvani, J., Feuillet, S. et al.	2017	A case of lung infection imported from the United-States	Annals Of Pathology	37	2	202-205	Case report	NA	NA	France	38	NA	NA	NA	NA	NA	NA	NA	NA	NA	NA	1	NA	NA	NA	1	NA	NA	1	NA	1
76	Camprubí-Ferrer, D., Cobuccio, L. et al.	2022	Causes of fever in returning TRAVELERS: A EUROPEAN MULTICENTER prospective cohort study	Journal Of Travel Medicine	NA	NA	NA	Cohort studies	2017-11-01	2019-11-30	Several	68	NA	NA	NA	NA	NA	NA	NA	NA	NA	NA	155	NA	NA	NA	NA	155	NA	NA	155	765
77	Camps, M., Vilella, A. et al.	2008	Incidence of respiratory viruses among travelers with a febrile syndrome returning from tropical and subtropical areas	Journal Of Medical Virology	80	4	711-715	Cross-sectional studies	2005-08-01	2006-10-31	Spain	62	73	NA	NA	NA	NA	73	NA	NA	73	118	44	NA	NA	NA	NA	44	NA	NA	44	118
78	Cassier, P., Campese, C. et al.	2014	Epidemiologic characteristics associated with ST23 clones compared to ST1 and ST47 clones of Legionnaires disease cases in France	New Microbes & New Infections	3	NA	29-33	Cohort studies	2008-01-01	2012-12-31	France	68	NA	NA	NA	NA	NA	NA	NA	NA	NA	NA	204	NA	NA	NA	NA	NA	NA	NA	NA	1192

Study ID	Authors	Publication Year	Title	Journal	Volume	Issue	Pages	Study Design	Starting date	Ending date	Country of detection	JBI Critical Score (%)	Respiratory Symptoms										Respiratory Cases Reported									
													Total cases (n)	Age Distribution					Gender Distribution			Total population (N)	Total cases (n)	Age Distribution					Gender Distribution			Total population (N)
														Child/Young	Adult	Middle Age Adult	Senior Adult	Unknown Age	Female	Male	Unknown Gender			Child/Young	Adult	Middle Age Adult	Senior Adult	Unknown Age	Female	Male	Unknown Gender	
79	Castillo, C., Nicklas, C. et al.	2006	Andes Hantavirus as possible cause of disease in travellers to South America	Travel Medicine & Infectious Disease	5	1	30-34	Cross-sectional studies	1997-01-01	2004-12-31	Chile	62	NA	NA	NA	NA	NA	NA	NA	NA	NA	NA	8	NA	NA	NA	NA	8	NA	NA	8	54
80	Catala, L., Rius, C. et al.	2012	Pandemic A/H1N1 influenza: Transmission of the first cases in Spain	Enfermedades Infecciosas Y Microbiologia Clinica	30	2	60-63	Case series	2009-04-15	2009-05-02	Spain	90	NA	NA	NA	NA	NA	NA	NA	NA	NA	NA	10	NA	10	NA	NA	NA	4	6	NA	71
81	Cdc Covid-19 Response Team	2021	SARS-CoV-2 B.1.1.529 (Omicron) Variant — United States, December 1–8, 2021	Morbidity & Mortality Weekly Report	70	50	1731-1734	Case series	2021-12-01	2021-12-08	United States of America	75	NA	NA	NA	NA	NA	NA	NA	NA	NA	NA	14	NA	NA	NA	NA	14	NA	NA	14	43
82	Ceyhan, M., Celik, M. et al.	2013	Acquisition of Meningococcal Serogroup W-135 Carriage in Turkish Hajj Pilgrims Who Had Received the Quadrivalent Meningococcal Polysaccharide Vaccine	Clinical & Vaccine Immunology	20	1	66-68	Cross-sectional studies	2010-11-14	2010-11-19	Turkey	62	NA	NA	NA	NA	NA	NA	NA	NA	NA	NA	39	NA	NA	NA	NA	39	NA	NA	39	472
83	Chamorro-Tojeiro, S., González-Sarria, A. et al.	2020	Acute pulmonary paracoccidioidomycosis in a traveler to Mexico	Journal Of Travel Medicine	27	4	NA	Case report	2019-05-01	2019-05-31	Spain	88	1	NA	NA	1	NA	NA	NA	1	NA	1	1	NA	NA	1	NA	NA	NA	1	NA	1
84	Chaw, L., Koh, W. C. et al.	2020	Analysis of SARS-CoV-2 Transmission in Different Settings, Brunei	Emerging Infectious Diseases	26	11	2598-2606	Cross-sectional studies	2020-02-27	2020-03-01	Brunei	100	42	NA	NA	NA	NA	NA	NA	NA	NA	75	19	1	9	4	5	19	NA	19	NA	75
85	Chen, J., He, H. et al.	2020	Potential transmission of SARS-CoV-2 on a flight from Singapore to Hangzhou, China: An epidemiological investigation	Travel Medicine & Infectious Disease	36	NA	NA	Case series	2020-01-24	2020-02-15	China	95	10	NA	6	4	NA	NA	6	4	NA	16	16	NA	9	6	NA	NA	10	6	NA	335
86	Chen, L. H., Leder, K. et al.	2019	Business travel-associated illness: a GeoSentinel analysis	Journal Of Travel Medicine	25	1	NA	Cross-sectional studies	1997-01-01	2014-12-31	Several	88	NA	NA	NA	NA	NA	NA	NA	NA	NA	NA	1403	NA	NA	NA	NA	1403	NA	NA	1403	14045
87	Chen, L. H., Wilson, M. E. et al.	2009	Illness in Long-Term Travelers Visiting GeoSentinel Clinics	Emerging Infectious Diseases	15	11	1773-1782	Cross-sectional studies	1996-06-01	2008-12-31	Several	69	NA	NA	NA	NA	NA	NA	NA	NA	NA	NA	NA	NA	NA	NA	NA	NA	NA	NA	NA	NA
88	Choi, E. M., Chu, D. K. W. et al.	2020	In Flight Transmission of Severe Acute Respiratory SARS-CoV-2	Journal Of Travel Medicine	10	5	299-300	Case series	2020-03-09	2020-03-10	China, Hong Kong Special Administrative Region	60	3	NA	NA	2	1	NA	2	1	NA	4	4	NA	1	2	1	NA	2	2	NA	NA
89	Choudhry, A. J., Al-Mudaimegh, K. S. et al.	2006	Hajj-associated acute respiratory infection among hajjis from Riyadh	Eastern Mediterranean Health Journal	12	3	300-309	Cohort studies	2002-02-20	2002-02-25	Saudi Arabia	59	NA	NA	NA	NA	NA	NA	NA	NA	NA	NA	409	NA	NA	NA	NA	409	106	303	NA	1027
90	Cido, D. D. S., Watts, A. et al.	2020	Routes for COVID-19 importation in Brazil	Journal Of Travel Medicine	27	3	NA	Case series	2020-01-01	2020-03-05	Brazil	50	NA	NA	NA	NA	NA	NA	NA	NA	NA	NA	29	NA	NA	NA	NA	29	NA	NA	29	34
91	Clancey, S. A., Ciccone, E. J. et al.	2019	Cryptococcus deuterogattii VGIIa Infection Associated with Travel to the Pacific Northwest Outbreak Region in an Anti-Granulocyte-Macrophage Colony-Stimulating Factor Autoantibody-Positive Patient in the United States	Biorxiv	NA	NA	493239	Case report	NA	NA	United States	88	1	NA	NA	NA	1	NA	NA	1	NA	1	1	NA	NA	NA	1	NA	NA	1	NA	1
92	Codreanu, T. A., Pingault, N. et al.	2021	The Healthy Crew, Clean Vessel, and Set Departure Date Triad: Successful Control of Outbreaks of COVID-19 On Board Four Cargo Vessels	Prehospital & Disaster Medicine	36	5	611-620	Cross-sectional studies	2020-05-07	2020-10-05	Australia	75	50	NA	NA	NA	NA	50	NA	NA	50	65	65	NA	NA	NA	NA	65	NA	NA	65	144
93	Control Centers For Disease Prevention	2000	Coccidioidomycosis in Travelers Returning From Mexico — Pennsylvania, 2000	NA	NA	NA	NA	Cross-sectional studies	2000-01-24	2000-02-02	United States	69	23	NA	NA	NA	NA	23	NA	NA	23	30	5	NA	NA	NA	NA	5	NA	NA	5	30
94	Control Centers For Disease Prevention	2001	Exposure to Patients With Meningococcal Disease on Aircrafts ---United States, 1999--2001	Morbidity & Mortality Weekly Report	50	23	485-9	Case report	1999-01-01	2001-12-31	United States of America	25	NA	NA	NA	NA	NA	NA	NA	NA	NA	NA	1	NA	NA	NA	1	NA	NA	1	NA	1
95	Control Centers For Disease Prevention	2001	Influenza B virus outbreak on a cruise ship–Northern Europe, 2000	Morbidity & Mortality Weekly Report	50	8	137-140	Cross-sectional studies	2000-06-23	2000-07-05	Several	62	118	NA	NA	NA	NA	118	NA	NA	118	1817	NA	NA	NA	NA	NA	NA	NA	NA	NA	NA
96	Control Centers For Disease Prevention	2001	Public Health Dispatch: Coccidioidomycosis Among Persons Attending the World Championship of Model Airplane Flying — Kern County, California, October 2001	NA	NA	NA	NA	Case report	2001-10-08	2001-12-04	United States	25	1	NA	NA	NA	1	NA	NA	NA	1	1	1	NA	NA	NA	1	NA	NA	NA	1	1
97	Control Centers For Disease Prevention	2001	Public Health Dispatch: Update: Assessment of Risk for Meningococcal Disease Associated With the Hajj 2001	Morbidity & Mortality Weekly Report	50	12	221-222	Cross-sectional studies	2001-02-16	2001-02-27	United States	62	NA	NA	NA	NA	NA	NA	NA	NA	NA	811	NA	NA	NA	NA	NA	NA	NA	NA	NA	NA
98	Control Centers For Disease Prevention	2000	Serogroup W-135 Meningococcal Disease Among Travelers Returning From Saudi Arabia --- United States, 2000	Morbidity & Mortality Weekly Report	49	16	345-346	Case series	2000-03-13	2000-04-20	United States	60	NA	NA	NA	NA	NA	NA	NA	NA	NA	NA	3	NA	NA	NA	NA	3	NA	NA	3	3
99	Control Centers For Disease Prevention	2007	Surveillance for travel-associated legionnaires disease–United States, 2005-2006	Morbidity & Mortality Weekly Report	56	48	1261-1263	Cross-sectional studies	2005-01-01	2006-12-31	United States	62	NA	NA	NA	NA	NA	NA	NA	NA	NA	NA	54	NA	NA	NA	NA	54	NA	NA	54	319

Study ID	Authors	Publication Year	Title	Journal	Volume	Issue	Pages	Study Design	Starting date	Ending date	Country of detection	JBI Critical Score (%)	Respiratory Symptoms										Respiratory Cases Reported									
													Total cases (n)	Age Distribution					Gender Distribution			Total population (N)	Total cases (n)	Age Distribution					Gender Distribution			Total population (N)
														Child/Young	Adult	Middle Age Adult	Senior Adult	Unknown Age	Female	Male	Unknown Gender			Child/Young	Adult	Middle Age Adult	Senior Adult	Unknown Age	Female	Male	Unknown Gender	
100	Control Centers For Disease Prevention	2001	Update: outbreak of acute febrile illness among athletes participating in Eco-Challenge-Sabah 2000–Borneo, Malaysia, 2000	Morbidity & Mortality Weekly Report	50	2	21-24	Cross-sectional studies	2000-08-21	2000-09-03	United States	62	NA	NA	NA	NA	NA	NA	NA	NA	NA	NA	32	NA	NA	NA	NA	68	NA	NA	68	304
101	Control Centers For Disease Prevention	2001	Update: Outbreak of Acute Febrile Respiratory Illness Among College Students --- Acapulco, Mexico, March 2001	Morbidity & Mortality Weekly Report	50	18	359-60	Prevalence studies	2001-03-01	2001-05-03	United States of America	39	NA	NA	NA	NA	NA	NA	NA	NA	NA	NA	28	NA	NA	NA	NA	NA	NA	NA	NA	111
102	Corpolongo, A., Giancola, M. L. et al.	2014	An imported case of acute pulmonary coccidioidomycosis in an Italian traveller	Infection	42	5	921-924	Case report	2012-08-26	2013-02-28	Italy	88	NA	NA	NA	1	NA	NA	NA	1	NA	1	1	NA	NA	1	NA	NA	NA	1	NA	1
103	Correa-Martinez, C. L., Kampmeier, S. et al.	2020	A Pandemic in Times of Global Tourism: Superspreading and Exportation of COVID-19 Cases from a Ski Area in Austria	Journal Of Clinical Microbiology	58	6	NA	Case series	2020-03-09	2020-03-16	Germany	85	36	NA	NA	NA	NA	36	14	22	NA	90	36	NA	NA	NA	NA	36	14	22	NA	90
104	Cottle, L. E., Gkrania-Klotsas, E. et al.	2013	A Multinational Outbreak of Histoplasmosis Following a Biology Field Trip in the Ugandan Rainforest	Journal Of Travel Medicine	20	2	83-87	Case series	2011-09-01	NA	Several	100	13	NA	12	NA	NA	1	7	6	NA	24	13	NA	12	NA	NA	1	7	6	NA	24
105	Crawshaw, A. F., Pareek, M. et al.	2018	Infectious disease testing of UK-bound refugees: a population-based, crosssectional study	BMC Medicine	16	1	143	Cross-sectional studies	2013-03-01	2017-08-01	UK	81	NA	NA	NA	NA	NA	NA	NA	NA	NA	NA	9	NA	NA	NA	NA	9	NA	NA	9	9759
106	Crepet, A., Repetto, E. et al.	2016	Lessons learnt from TB screening in closed immigration centres in Italy	International Health	8	5	324-329	Cross-sectional studies	2012-06-01	2013-12-31	Italy	75	NA	NA	NA	NA	NA	NA	NA	NA	NA	NA	3	NA	NA	NA	NA	3	NA	NA	3	3588
107	Da Silva Filipe, A., Shepherd, J. G. et al.	2020	Genomic epidemiology reveals multiple introductions of SARS-CoV-2 from mainland Europe into Scotland	Nature Microbiology	6	1	112-+	Cross-sectional studies	2020-01-01	2020-03-31	UK	62	NA	NA	NA	NA	NA	NA	NA	NA	NA	NA	114	NA	NA	NA	NA	114	NA	NA	114	1090
108	Dabrera, G., Brsema, P. et al.	2017	Increase in Legionnaires' disease cases associated with travel to Dubai among travellers from the United Kingdom, Sweden and the Netherlands, October 2016 to end August 2017	Eurosurveillance	22	38	NA	Case series	2016-10-01	2017-08-31	Several	80	NA	NA	NA	NA	NA	NA	NA	NA	NA	NA	51	NA	NA	NA	NA	51	NA	NA	51	51
109	Dahl, V., Wallensten, A.	2017	Self-reported infections during international travel and notifiable infections among returning international travellers, Sweden, 2009-2013 diseases: A cross-sectional analysis	PLOS One	12	7	NA	Cross-sectional studies	2009-01-01	2013-12-31	Sweden	62	NA	NA	NA	NA	NA	NA	NA	NA	NA	NA	103	NA	NA	NA	NA	103	NA	NA	103	NA
110	Dam Larsen, F., Jespersen, S. et al.	2017	One-sixth of inpatients in a Danish infectious disease ward have imported diseases: A cross-sectional analysis	Travel Medicine & Infectious Disease	20	NA	43-48	Cross-sectional studies	2015-01-01	2015-12-31	Denmark	62	NA	NA	NA	NA	NA	NA	NA	NA	NA	NA	52	NA	NA	NA	NA	52	NA	NA	52	240
111	Dao, T. L., Canard, N. et al.	2020	Risk factors for symptoms of infection and microbial carriage among French medical students abroad	International Journal Of Infectious Diseases	100	NA	104-111	Cross-sectional studies	2018-01-01	2019-12-31	France	88	105	NA	NA	NA	NA	105	NA	NA	105	293	144	NA	NA	NA	NA	144	NA	NA	144	275
112	Davis, X. M., Hay, K. A. et al.	2013	International Travelers as Sentinels for Sustained Influenza Transmission During the 2009 Influenza A(H1N1)pdm09 Pandemic	Journal Of Travel Medicine	20	3	177-184	Cross-sectional studies	2009-04-01	2009-10-24	Several	94	NA	NA	NA	NA	NA	NA	NA	NA	NA	NA	673	75	174	NA	NA	423	329	343	NA	4143
113	Davis, X. M., Macdonald, S. et al.	2008	Health risks in travelers to China: the GeoSentinel experience and implications for the 2008 Beijing Olympics	American Journal Of Tropical Medicine & Hygiene	79	1	4-8	Cross-sectional studies	1998-01-01	2007-11-30	Several	88	NA	NA	NA	NA	NA	NA	NA	NA	NA	NA	1431	NA	NA	NA	NA	1431	NA	NA	1431	15267
114	De Jong, B., Payne Hallstrm, L. et al.	2013	Travel-associated Legionnaires disease in Europe, 2010	Eurosurveillance	18	23	NA	Cross-sectional studies	2009-01-01	2010-12-31	Several	75	NA	NA	NA	NA	NA	NA	NA	NA	NA	NA	1685	NA	NA	NA	265	1420	240	624	821	1685
115	De Souza Luna, L. K., Panning, M. et al.	2007	Spectrum of Viruses and Atypical Bacteria in Intercontinental Air Travelers with Symptoms of Acute Respiratory Infection	Journal Of Infectious Diseases	195	5	675-679	Cross-sectional studies	NA	NA	Several	62	NA	NA	NA	NA	NA	NA	NA	NA	NA	NA	67	NA	NA	NA	NA	67	NA	NA	67	155
116	Deris, Z. Z., Hasan, H. et al.	2010	The Prevalence of Acute Respiratory Symptoms and Role of Protective Measures Among Malaysian Hajj Pilgrims	Journal Of Travel Medicine	17	2	82-88	Cross-sectional studies	2007-12-17	2007-12-22	Malaysia	88	NA	NA	NA	NA	NA	NA	NA	NA	NA	387	NA	NA	NA	NA	NA	NA	NA	NA	NA	387
117	Desenclos, J. C., Van Der Werf, S. et al.	2004	Introduction of SARS in France, MarchApril, 2003	Emerging Infectious Diseases	10	2	195-200	Case series	2003-03-12	2003-04-30	France	75	4	NA	NA	NA	NA	4	NA	NA	4	4	4	NA	NA	NA	NA	4	NA	NA	4	4
118	Devaux, I., Kreidl, P. et al.	2010	Initial surveillance of 2009 influenza A(H1N1) pandemic in the European Union and European Economic Area, April – September 2009	Eurosurveillance	15	49	6-16	Cross-sectional studies	2009-06-01	2009-09-30	Several	75	NA	NA	NA	NA	NA	NA	NA	NA	NA	NA	3542	NA	NA	NA	NA	3542	NA	NA	3542	10643

Study ID	Authors	Publication Year	Title	Journal	Volume	Issue	Pages	Study Design	Starting date	Ending date	Country of detection	JBI Critical Score (%)	Respiratory Symptoms										Respiratory Cases Reported									
													Total cases (n)	Age Distribution					Gender Distribution			Total population (N)	Total cases (n)	Age Distribution					Gender Distribution			Total population (N)
														Child/Young	Adult	Middle Age Adult	Senior Adult	Unknown Age	Female	Male	Unknown Gender			Child/Young	Adult	Middle Age Adult	Senior Adult	Unknown Age	Female	Male	Unknown Gender	
119	Dhanasekaran, V., Edwards, K. M. et al.	2021	Air travel-related outbreak of multiple SARS-CoV-2 variants	Journal Of Travel Medicine	28	8	NA	Cross-sectional studies	2020-06-01	2021-04-30	China	62	NA	NA	NA	NA	NA	NA	NA	NA	NA	NA	59	NA	NA	NA	NA	59	NA	NA	59	146
120	Di Meco, E., Di Napoli, A. et al.	2018	Infectious and dermatological diseases among arriving migrants on the Italian coasts	European Journal Of Public Health	28	5	910-916	Cross-sectional studies	2015-01-01	2016-12-31	Italy	62	NA	NA	NA	NA	NA	NA	NA	NA	NA	NA	358	NA	NA	NA	NA	358	NA	NA	358	6188
121	Dia, A., Gautret, P. et al.	2010	Illness in French Travelers to Senegal: Prospective Cohort Followup and Sentinel Surveillance Data	Journal Of Travel Medicine	17	5	296-302	Cross-sectional studies	2008-01-01	2008-12-31	France	100	NA	NA	NA	NA	NA	NA	NA	NA	26	358	NA	NA	NA	NA	NA	NA	NA	NA	NA	NA
122	Diel, R., Rsch-Gerdes, S. et al.	2004	Molecular Epidemiology of Tuberculosis among Immigrants in Hamburg, Germany	Journal Of Clinical Microbiology	42	7	2952-2960	Cross-sectional studies	1997-01-01	2002-06-30	Germany	100	NA	NA	NA	NA	NA	NA	NA	NA	NA	NA	97	NA	NA	NA	NA	97	NA	NA	97	108
123	Diz, S., Lopez-Velez, R. et al.	2007	Epidemiology and clinical features of tuberculosis in immigrants at an infectious diseases department in Madrid	International Journal Of Tuberculosis & Lung Disease	11	7	769-774	Cross-sectional studies	1990-01-01	2002-12-31	Spain	75	NA	NA	NA	NA	NA	NA	NA	NA	NA	NA	98	NA	NA	NA	NA	98	NA	NA	98	1353
124	Dollard P., Griffin I. et al.	2020	Risk Assessment and Management of COVID-19 Among Travelers Arriving at Designated U.S. Airports, January 17–September 13, 2020	Morbidity & Mortality Weekly Report	69	45	1681-1685	Cross-sectional studies	2020-01-17	2020-09-13	United States	62	278	NA	NA	NA	NA	278	NA	NA	278	766044	23	NA	NA	NA	NA	23	NA	NA	23	766044
125	Duncan, A. R., Priest, P. C. et al.	2009	Screening for Influenza Infection in International Airline Travelers	American Journal Of Public Health	99	NA	S360-S362	Cross-sectional studies	2007-07-01	2007-12-31	New Zealand	62	NA	NA	NA	NA	NA	NA	NA	NA	NA	359	1	NA	NA	NA	NA	1	NA	NA	1	103
126	Dunne, E. M., Maxwell, T. et al.	2021	Investigation and public health response to a COVID-19 outbreak in a rural resort community—Blaine County, Idaho, 2020	PLOS One	16	4	NA	Cross-sectional studies	2020-03-13	2020-04-10	United States	62	NA	NA	NA	NA	NA	NA	NA	NA	NA	NA	44	NA	NA	NA	NA	44	NA	NA	44	450
127	El Bashir, H., Haworth, E. et al.	2004	Influenza among U.K. Pilgrims to Hajj, 2003	Emerging Infectious Diseases	10	10	1882-1883	Cross-sectional studies	2002-12-01	2003-01-31	UK	62	93	NA	NA	NA	NA	93	NA	NA	93	115	44	NA	NA	NA	NA	44	NA	NA	44	115
128	Emamian, M. H., Hassani, A. M. et al.	2013	Respiratory Tract Infections and its Preventive Measures among Hajj Pilgrims, 2010: A Nested Case Control Study	International Journal Of Preventive Medicine	4	9	1030-1035	Case control	2010-11-14	2010-11-19	Iran (Islamic Republic of)	85	NA	NA	NA	NA	NA	NA	NA	NA	NA	NA	32	NA	NA	NA	18	14	12	20	NA	338
129	Epelboin, L., Blonde, R. et al.	2020	COVID-19 and dengue co-infection in a returning traveller	Journal Of Travel Medicine	27	6	NA	Case report	2020-03-06	2020-03-14	France	75	1	NA	NA	1	NA	NA	NA	1	NA	1	1	NA	NA	1	NA	NA	NA	1	NA	1
130	Erdem, H., Ak, O. et al.	2016	Infections in travellers returning to Turkey from the Arabian peninsula: a retrospective cross-sectional multicenter study	European Journal Of Clinical Microbiology & Infectious Diseases	35	6	903-910	Cross-sectional studies	2013-01-01	2015-03-01	Turkey	81	NA	NA	NA	NA	NA	NA	NA	NA	NA	NA	169	NA	NA	NA	NA	169	NA	NA	169	185
131	Esposito, D. H., Han, P. V. et al.	2012	Characteristics and Spectrum of Disease Among Ill Returned Travelers from Pre- and Post-Earthquake Haiti: The GeoSentinel Experience	American Journal Of Tropical Medicine & Hygiene	86	1	23-28	Cross-sectional studies	2007-01-13	2011-01-12	Several	69	NA	NA	NA	NA	NA	NA	NA	NA	NA	NA	31	NA	NA	NA	NA	31	NA	NA	31	280
132	Fang, L. Q., Sun, Y. et al.	2018	Travel-related infections in mainland China, 2014–16: an active surveillance study	Lancet Public Health	3	8	E385-E394	Cross-sectional studies	2014-01-01	2016-12-31	China	94	NA	NA	NA	NA	NA	NA	NA	NA	NA	NA	19662	6340	6759	4850	1627	86	6534	13128	NA	805993392
133	Fanoy, E. B., Van Der Sande, M. A. et al.	2014	Travel-related MERS-CoV cases: an assessment of exposures and risk factors in a group of Dutch travellers returning from the Kingdom of Saudi Arabia, May 2014	Emerging Themes In Epidemiology	11	NA	16-16	Case series	2014-04-01	2014-05-31	Netherlands	80	NA	NA	NA	NA	NA	NA	NA	NA	NA	NA	2	NA	NA	NA	2	NA	1	1	NA	29
134	Farooq, H. Z., Davies, E. et al.	2020	Middle East respiratory syndrome coronavirus (MERS-CoV) — Surveillance and testing in North England from 2012 to 2019	International Journal Of Infectious Diseases	93	NA	237-244	Cross-sectional studies	2012-01-01	2019-02-01	UK	75	NA	NA	NA	NA	NA	NA	NA	NA	NA	NA	264	16	NA	NA	93	155	103	161	NA	264
135	Fenner, L., Weber, R. et al.	2007	Imported Infectious Disease and Purpose of Travel, Switzerland	Emerging Infectious Diseases	13	2	217-222	Cohort studies	2004-01-01	2005-06-01	Switzerland	73	86	NA	NA	NA	NA	86	NA	NA	86	338	62	NA	NA	NA	NA	62	NA	NA	62	338
136	Fernes, E. G., De Souza, P. B. et al.	2014	Influenza B Outbreak on a Cruise Ship off the São Paulo Coast, Brazil	Journal Of Travel Medicine	21	5	298-303	Cross-sectional studies	2012-02-15	2012-02-27	Several	94	NA	NA	NA	NA	NA	NA	NA	NA	NA	104	104	16	NA	NA	1	87	56	NA	NA	2458
137	Ferson, M., Paraskevopoulos, P. et al.	2000	Presumptive summer influenza A: an outbreak on a trans-Tasman cruise	Communicable Diseases Intelligence	24	3	45-47	Case series	2000-02-01	2000-02-16	Australia	50	108	NA	NA	NA	NA	108	NA	NA	108	1479	NA	NA	NA	NA	NA	NA	NA	NA	NA	NA
138	Ficko, C., Andriamanantena, D. et al.	2012	Mycoplasma pneumoniae: a cause of febrile hemolytic anemia in travelers	Médecine & Santé Tropicales	22	4	344-5	Case report	NA	NA	France	62	NA	NA	1	NA	NA	NA	NA	1	NA	1	1	NA	1	NA	NA	NA	NA	1	NA	1
139	Field, V., Gautret, P. et al.	2010	Travel and migration associated infectious diseases morbidity in Europe, 2008	BMC Infectious Diseases	10	NA	NA	Cross-sectional studies	2008-01-01	2008-12-31	Several	88	NA	NA	NA	NA	NA	NA	NA	NA	NA	NA	540	NA	NA	NA	NA	540	NA	NA	540	6957

Study ID	Authors	Publication Year	Title	Journal	Volume	Issue	Pages	Study Design	Starting date	Ending date	Country of detection	JBI Critical Score (%)	Respiratory Symptoms										Respiratory Cases Reported									
													Total cases (n)	Age Distribution					Gender Distribution			Total population (N)	Total cases (n)	Age Distribution					Gender Distribution			Total population (N)
														Child/Young	Adult	Middle Age Adult	Senior Adult	Unknown Age	Female	Male	Unknown Gender			Child/Young	Adult	Middle Age Adult	Senior Adult	Unknown Age	Female	Male	Unknown Gender	
140	Firestone, M. J., Lorentz, A. et al.	2021	First Identified Cases of SARS-CoV-2 Variant P.1 in the United States — Minnesota, January 2021	Morbidity & Mortality Weekly Report	70	10	346-347	Case series	2021-01-01	2021-01-31	Several	50	NA	NA	NA	NA	NA	NA	NA	NA	NA	NA	3	NA	NA	NA	NA	3	NA	NA	3	3
141	Fishbein, D., Sandoval, M. et al.	2010	Public Health Surveillance Using Emergency Medical Service Logs --- U.S.--Mexico Land Border, El Paso, Texas, 2009	Morbidity & Mortality Weekly Report	59	21	649-653	Cross-sectional studies	2009-01-01	2009-12-31	United States	56	NA	NA	NA	NA	NA	NA	NA	NA	NA	NA	34	NA	NA	NA	NA	34	NA	NA	34	51074
142	Flores-Figueroa, J., Okhuysen, P. C. et al.	2011	Patterns of Illness in Travelers Visiting Mexico and Central America: The GeoSentinel Experience	Clinical Infectious Diseases	53	6	523-531	Cross-sectional studies	1996-12-01	2010-02-28	Several	88	NA	NA	NA	NA	NA	NA	NA	NA	NA	NA	330	NA	NA	NA	NA	330	NA	NA	330	4779
143	Fohlman, J., Benni, H. et al.	2009	Coccidioidomycosis as Imported Atypical Pneumonia in Sweden	Scandinavian Journal Of Infectious Diseases	32	4	440-441	Case report	NA	NA	Sweden	75	1	NA	NA	NA	1	NA	NA	1	NA	1	1	NA	NA	NA	1	NA	NA	1	NA	1
144	Follin, P., Lindqvist, A. et al.	2009	A variety of respiratory viruses found in symptomatic travellers returning from countries with ongoing spread of the new influenza A(H1N1)v virus strain	Eurosurveillance	14	24	NA	Cross-sectional studies	2009-04-24	2009-06-10	Sweden	62	71	NA	NA	NA	NA	71	NA	NA	71	79	47	NA	NA	NA	NA	47	NA	NA	47	79
145	Foo, H., Blyth, C.c. et al.	2009	Laboratory test performance in young adults during influenza outbreaks at World Youth Day 2008	Journal Of Clinical Virology	46	4	384-386	Cross-sectional studies	2008-07-15	2008-07-20	Australia	62	227	NA	NA	NA	NA	227	NA	NA	227	227	95	NA	NA	NA	NA	95	NA	NA	95	230
146	Foxwell, A. R.	2011	Transmission of Influenza on International Flights, May 2009	Emerging Infectious Diseases	17	7	1188-1194	Cohort studies	2009-05-23	2009-05-24	Australia	59	NA	NA	NA	NA	NA	NA	NA	NA	NA	319	9	NA	NA	NA	NA	9	NA	NA	9	319
147	Freedman, D. O., Weld, L. H. et al.	2006	Spectrum of Disease and Relation to Place of Exposure among Ill Returned Travelers	New England Journal Of Medicine	354	2	119-130	Cross-sectional studies	1996-06-01	2004-08-31	Several	88	NA	NA	NA	NA	NA	NA	NA	NA	NA	NA	1891	NA	NA	NA	NA	77	NA	NA	77	17353
148	Fujino, T., Nomoto, H. et al.	2021	Novel SARS-CoV-2 Variant in Travelers from Brazil to Japan	Emerging Infectious Diseases	27	4	1243-1245	Case report	2021-01-02	2021-01-31	Japan	62	1	NA	NA	1	NA	3	NA	1	3	4	4	NA	NA	1	NA	3	NA	1	3	4
149	Furuya-Kanamori, L., Mills, D. et al.	2017	Medical and psychological problems faced by young Australian gap year travellers	Journal Of Travel Medicine	24	5	NA	Cross-sectional studies	2013-12-01	2014-04-30	Australia	88	NA	NA	NA	NA	NA	NA	NA	NA	NA	NA	22	NA	NA	NA	NA	22	NA	NA	22	34
150	Ganaie, F., Nagaraj, G. et al.	2018	Impact of Hajj on the S. pneumoniae carriage among Indian pilgrims during 2016-a longitudinal molecular surveillance study	Travel Medicine & Infectious Disease	23	NA	64-71	Cohort studies	2016-09-10	2016-09-15	India	77	NA	NA	NA	NA	NA	NA	NA	NA	NA	807	133	NA	NA	NA	NA	133	NA	NA	133	807
151	Gastanaduy, P. A.	2013	Update: Severe Respiratory Illness Associated with Middle East Respiratory Syndrome Coronavirus (MERS-CoV) — Worldwide, 2012–2013	Morbidity & Mortality Weekly Report	62	23	480-483	Case series	2012-04-01	2013-05-29	United States	55	NA	NA	NA	NA	NA	NA	NA	NA	NA	NA	10	NA	NA	NA	NA	10	NA	NA	10	55
152	Gautier, M., Michel, J. et al.	2015	Cochliobolus hawaiiensis Sinusitis, a Tropical Disease? A Case Report and Review of the Literature	Mycopathologia	180	1	117-121	Case report	NA	NA	France	94	1	NA	NA	1	NA	NA	NA	1	NA	1	1	NA	NA	1	NA	NA	NA	1	NA	1
153	Gautret, P., Angelo, K. M. et al.	2019	International mass gatherings and travel-associated illness: A GeoSentinel cross-sectional, observational study	Travel Medicine & Infectious Disease	32	NA	NA	Cross-sectional studies	2015-08-17	2019-04-30	Several	81	NA	NA	NA	NA	NA	NA	NA	NA	NA	NA	NA	NA	NA	NA	NA	NA	NA	NA	NA	260
154	Gautret, P., Benkouiten, S. et al.	2015	The inevitable Hajj cough: Surveillance data in French pilgrims, 2012–2014	Travel Medicine & Infectious Disease	13	6	485-489	Cross-sectional studies	2012-01-01	2014-12-31	France	56	309	NA	NA	NA	NA	309	NA	NA	309	382	NA	NA	NA	NA	NA	NA	NA	NA	NA	NA
155	Gautret, P., Charrel, R. et al.	2013	Lack of nasal carriage of novel corona virus (HCoV-EMC) in French Hajj pilgrims returning from the Hajj 2012, despite a high rate of respiratory symptoms	Clinical Microbiology & Infection	19	7	E315-E317	Cross-sectional studies	2012-09-01	2012-11-30	France	62	142	NA	NA	NA	NA	142	NA	NA	142	157	0	NA	NA	NA	NA	0	NA	NA	0	154
156	Gautret, P., Cramer, J. P. et al.	2012	Infectious diseases among travellers and migrants in Europe, EuroTravNet 2010	Eurosurveillance	17	26	16-26	Cross-sectional studies	2008-01-01	2010-12-31	Several	75	NA	NA	NA	NA	NA	NA	NA	NA	NA	NA	632	NA	NA	NA	NA	632	NA	NA	632	27488
157	Gautret, P., Gaudart, J. et al.	2012	Travel-Associated Illness in Older Adults (>60 y)	Journal Of Travel Medicine	19	3	169-177	Cross-sectional studies	1997-01-01	2009-12-31	Several	88	6799	NA	NA	NA	1027	5772	NA	NA	6799	63076	6958	NA	NA	NA	NA	6958	NA	NA	6958	63076
158	Gautret, P., Parola, P. et al.	2013	Relative risk for influenza like illness in French Hajj pilgrims compared to non-Hajj attending controls during the 2009 influenza pandemic	Travel Medicine & Infectious Disease	11	2	95-97	Cross-sectional studies	2009-10-26	2009-12-27	France	62	NA	NA	NA	NA	NA	NA	NA	NA	NA	273	NA	NA	NA	NA	NA	NA	NA	NA	NA	NA
159	Gautret, P., Soula, G. et al.	2009	Common Health Hazards in French Pilgrims During the Hajj of 2007: A Prospective Cohort Study	Journal Of Travel Medicine	16	6	377-381	Cross-sectional studies	2007-10-22	2007-12-06	France	94	NA	NA	NA	NA	NA	NA	NA	NA	NA	462	NA	NA	NA	NA	NA	NA	NA	NA	NA	NA

Study ID	Authors	Publication Year	Title	Journal	Volume	Issue	Pages	Study Design	Starting date	Ending date	Country of detection	JBI Critical Score (%)	Respiratory Symptoms										Respiratory Cases Reported									
													Total cases (n)	Age Distribution					Gender Distribution			Total population (N)	Total cases (n)	Age Distribution					Gender Distribution			Total population (N)
														Child/Young	Adult	Middle Age Adult	Senior Adult	Unknown Age	Female	Male	Unknown Gender			Child/Young	Adult	Middle Age Adult	Senior Adult	Unknown Age	Female	Male	Unknown Gender	
160	Gautret, P., Vu Hai, V. et al.	2010	Protective Measures Against Acute Respiratory Symptoms in French Pilgrims Participating in the Hajj of 2009	Journal Of Travel Medicine	18	1	53-55	Cross-sectional studies	2009-10-07	2009-11-06	France	62	NA	NA	NA	NA	NA	NA	NA	NA	NA	274	NA	NA	NA	NA	NA	NA	NA	NA	NA	NA
161	Gautret, P., Yong, W. et al.	2014	Incidence of Hajj-associated febrile cough episodes among French pilgrims: a prospective cohort study on the influence of statin use and risk factors	Clinical Microbiology & Infection	15	4	335-340	Cross-sectional studies	2006-11-04	2006-12-08	France	100	NA	NA	NA	NA	NA	NA	NA	NA	NA	447	NA	NA	NA	NA	NA	NA	NA	NA	NA	NA
162	German, M., Olsha, R. et al.	2015	Acute Respiratory Infections in Travelers Returning from MERS-CoV–Affected Areas	Emerging Infectious Diseases	21	9	1654-1656	Cross-sectional studies	2012-11-01	2014-06-30	Canada	69	NA	NA	NA	NA	NA	NA	NA	NA	NA	NA	89	NA	NA	NA	NA	89	NA	NA	89	177
163	Ghinai, I., Mcpherson, T. D. et al.	2020	First known person-to-person transmission of severe acute respiratory syndrome coronavirus 2 (SARS-CoV-2) in the USA	Lancet	395	10230	1137-1144	Case report	2020-01-15	2020-01-31	United States	75	1	NA	NA	NA	1	NA	1	NA	NA	1	1	NA	NA	NA	1	NA	1	NA	NA	1
164	Gobbi, F., Angheben, A. et al.	2012	Coccidioidomycosis: First Imported Case in Italy	Journal Of Travel Medicine	19	3	192-194	Case report	2007-07-15	2008-01-18	Italy	88	1	NA	1	NA	NA	NA	NA	1	NA	1	1	NA	1	NA	NA	NA	NA	1	NA	1
165	Goel, V., Bulir, D. et al.	2021	COVID-19 International Border Surveillance Cohort Study at Toronto's Pearson Airport	Medrxiv	NA	NA	2021.02.25.21252404	Cross-sectional studies	2020-09-01	2021-10-31	Canada	81	NA	NA	NA	NA	NA	NA	NA	NA	NA	NA	248	NA	NA	NA	4	244	107	141	NA	16361
166	Goldsmid, J. M., Sharples, N. et al.	2006	A Preliminary Study on Travel Health Issues of Medical Students Undertaking Electives	Journal Of Travel Medicine	10	3	160-162	Prevalence studies	NA	NA	Australia	89	NA	NA	NA	NA	NA	NA	NA	NA	NA	NA	8	NA	NA	NA	NA	8	NA	NA	8	109
167	Goumballa, N., Hoang, V.t. et al.	2020	Lack of Neisseria meningitidis among pilgrims during the 2017, 2018 and 2019 Grand Magal of Touba, Senegal	Clinical Microbiology & Infection	26	12	1697-1698	Cross-sectional studies	2017-01-01	2019-12-31	Senegal	62	132	NA	NA	NA	NA	132	NA	NA	132	304	NA	NA	NA	NA	NA	NA	NA	NA	NA	NA
168	Graham, F. F., White, P. S. et al.	2011	Changing epidemiological trends of legionellosis in New Zealand, 1979–2009	Epidemiology & Infection	140	8	1481-1496	Cross-sectional studies	1979-01-01	2009-12-31	New Zealand	69	NA	NA	NA	NA	NA	NA	NA	NA	NA	NA	52	NA	NA	NA	NA	52	NA	NA	52	805
169	Griffiths, K. M., Savini, H. et al.	2018	Surveillance of travel-associated diseases at two referral centres in Marseille, France: a 12-year survey	Journal Of Travel Medicine	25	NA	NA	Cross-sectional studies	2003-01-01	2015-10-31	France	94	331	NA	NA	NA	NA	331	NA	NA	331	3460	NA	NA	NA	NA	NA	NA	NA	NA	NA	NA
170	Grobusch, M. P., Weld, L. et al.	2020	Travel-related infections presenting in Europe: A 20-year analysis of EuroTravNet surveillance data	Lancet Regional Health-Europe	1	NA	NA	Cross-sectional studies	1998-03-01	2018-03-31	Several	94	NA	NA	NA	NA	NA	NA	NA	NA	NA	NA	NA	NA	NA	NA	NA	NA	NA	NA	NA	103739
171	Gu, H., Chu, D. K. W. et al.	2021	Genetic Diversity of SARS-CoV-2 among Travelers Arriving in Hong Kong	Emerging Infectious Diseases	27	10	2666-2668	Case series	2020-01-01	2021-03-31	China, Hong Kong Special Administrative Region	60	NA	NA	NA	NA	NA	NA	NA	NA	NA	NA	2192	NA	NA	NA	NA	2192	NA	NA	2192	2192
172	Gu, W., Deng, X. et al.	2020	Associations of Early COVID-19 Cases in San Francisco With Domestic and International Travel	Clinical Infectious Diseases	71	11	2976-2980	Cross-sectional studies	2020-03-10	2020-03-20	United States	69	NA	NA	NA	NA	NA	NA	NA	NA	NA	NA	6	NA	NA	NA	NA	6	NA	NA	6	46
173	Gudbjartsson, D. F., Helgason, A. et al.	2020	Spread of SARS-CoV-2 in the Icelandic Population	New England Journal Of Medicine	382	24	2302-2315	Cross-sectional studies	2020-01-31	2020-04-04	Iceland	88	NA	NA	NA	NA	NA	NA	NA	NA	NA	NA	297	NA	NA	NA	NA	297	NA	NA	297	1321
174	Guery, B., Poissy, J. et al.	2013	Clinical features and viral diagnosis of two cases of infection with Middle East Respiratory Syndrome coronavirus: a report of nosocomial transmission	Lancet	381	9885	2265-2272	Case report	2013-04-01	2013-05-31	France	88	1	NA	NA	NA	1	NA	NA	1	NA	1	1	NA	NA	NA	1	NA	NA	1	NA	1
175	Gundlapalli, A. V., Rubin, M. A. et al.	2006	Influenza, Winter Olympiad, 2002	Emerging Infectious Diseases	12	1	144-146	Cross-sectional studies	2002-02-01	2002-03-31	United States	100	NA	NA	NA	NA	NA	NA	NA	NA	NA	188	36	NA	NA	NA	NA	36	NA	28	8	188
176	Guner, A. E., Surmeli, A. et al.	2021	First known COVID-19 case and contact tracing efforts in Istanbul, Turkey	Turkish Journal Of Medical Sciences	51	4	1653-1658	Case series	2020-03-11	NA	Turkey	55	2	NA	2	NA	NA	NA	NA	2	NA	2	2	NA	2	NA	NA	NA	NA	2	NA	2
177	Gupta, N., John, A. et al.	2021	Clinical profile and outcomes of asymptomatic vs. symptomatic travellers diagnosed with COVID-19: An observational study from a coastal town in South India	Drug Discoveries & Therapeutics	15	1	1-8	Cross-sectional studies	2020-05-01	2020-09-10	India	81	NA	NA	NA	NA	NA	NA	NA	NA	NA	NA	12	NA	NA	NA	NA	12	NA	NA	12	127
178	Gutirrez, I., Litzroth, A. et al.	2009	Community transmission of influenza A (H1N1)v virus at a rock festival in Belgium, 2-5 July 2009	Eurosurveillance	14	31	NA	Cross-sectional studies	2009-05-11	2009-07-13	Belgium	62	NA	NA	NA	NA	NA	NA	NA	NA	NA	NA	12	NA	NA	NA	NA	12	NA	NA	12	30
179	Ha-Linh, Q., Ngoc-Anh Thi, H. et al.	2021	Successful containment of a flight-imported COVID-19 outbreak through extensive contact tracing, systematic testing and mandatory quarantine: Lessons from Vietnam	Travel Medicine & Infectious Disease	42	NA	NA	Cross-sectional studies	2020-03-01	2020-03-31	Vietnam	69	10	NA	NA	NA	NA	10	NA	NA	10	15	15	NA	NA	NA	NA	15	NA	NA	15	183
180	Hampton, L. M., Garrison, L. et al.	2016	Legionnaires' Disease Outbreak at a Resort in Cozumel, Mexico	Open Forum Infectious Diseases	3	3	NA	Case series	2008-05-01	2010-04-01	United States	65	NA	NA	NA	NA	NA	NA	NA	NA	NA	NA	9	NA	NA	NA	NA	9	4	5	NA	9

Study ID	Authors	Publication Year	Title	Journal	Volume	Issue	Pages	Study Design	Starting date	Ending date	Country of detection	JBI Critical Score (%)	Respiratory Symptoms										Respiratory Cases Reported									
													Total cases (n)	Age Distribution					Gender Distribution			Total population (N)	Total cases (n)	Age Distribution					Gender Distribution			Total population (N)
														Child/Young	Adult	Middle Age Adult	Senior Adult	Unknown Age	Female	Male	Unknown Gender			Child/Young	Adult	Middle Age Adult	Senior Adult	Unknown Age	Female	Male	Unknown Gender	
181	Han, K., Zhu, X. et al.	2009	Lack of Airborne Transmission during Outbreak of Pandemic (H1N1) 2009 among Tour Group Members, China, June 2009	Emerging Infectious Diseases	15	10	1578-1581	Cross-sectional studies	2009-05-27	2009-06-12	China	69	NA	NA	NA	NA	NA	NA	NA	NA	NA	11	17	NA	NA	NA	NA	17	NA	NA	17	405
182	Hannawi, S., Hannawi, H. et al.	2021	Clinical and Laboratory Profile of Hospitalized Symptomatic COVID-19 Patients: Case Series Study From the First COVID-19 Center in the UAE	Frontiers In Cellular & Infection Microbiology	11	NA	NA	Cross-sectional studies	2020-03-01	2020-08-31	United Arab Emirates	81	NA	NA	NA	NA	NA	NA	NA	NA	NA	NA	29	NA	NA	NA	NA	29	NA	NA	29	525
183	Harvey, K., Esposito, D. H. et al.	2013	Surveillance for travel-related disease–GeoSentinel Surveillance System, United States, 1997-2011	Morbidity & Mortality Weekly Report	62	NA	1-23	Cross-sectional studies	1997-09-01	2011-12-31	Several	62	NA	NA	NA	NA	NA	NA	NA	NA	NA	NA	1081	NA	NA	NA	NA	1081	NA	NA	1081	13059
184	Hashem, A. M., Al-Subhi, T. L. et al.	2019	MERS-CoV, influenza and other respiratory viruses among symptomatic pilgrims during 2014 Hajj season	Journal Of Medical Virology	91	6	911-917	Cross-sectional studies	2014-10-03	2014-10-07	Saudi Arabia	100	132	NA	NA	NA	63	61	44	88	NA	132	67	NA	NA	NA	36	26	25	42	NA	132
185	Hashim, S., Ayub, Z. N. et al.	2016	The prevalence and preventive measures of the respiratory illness among Malaysian pilgrims in 2013 hajj season	Journal Of Travel Medicine	23	2	NA	Prevalence studies	2013-10-01	2013-10-31	Malyasia	89	437	NA	NA	NA	NA	437	NA	NA	437	468	NA	NA	NA	NA	NA	NA	NA	NA	NA	NA
186	Hassan, S., Imtiaz, R. et al.	2013	Public health surveillance at a mass gathering: urs of Baba Farid, Pakpattan district, Punjab, Pakistan, December 2010	Eastern Mediterranean Health Journal	19	NA	S24-S28	Cross-sectional studies	2010-12-01	2010-12-31	Pakistan	62	NA	NA	NA	NA	NA	NA	NA	NA	NA	NA	721	NA	NA	NA	NA	721	NA	NA	721	5918
187	Hatzianastasiou, S., Mouchtouri, V. A. et al.	2021	COVID-19 Outbreak on a Passenger Ship and Assessment of Response Measures, Greece, 2020	Emerging Infectious Diseases	27	7	1927-1930	Cross-sectional studies	2020-03-07	2020-03-28	Greece	94	69	NA	NA	NA	NA	69	NA	NA	69	128	128	NA	NA	NA	NA	128	NA	NA	128	383
188	Hirata, K., Ogawa, T. et al.	2018	Characteristics of health problems in returned overseas travelers at a tertiary teaching hospital in a suburban area in Japan	Journal Of Infection & Chemotherapy	24	8	682-685	Cross-sectional studies	2008-01-01	2016-12-31	Japan	75	NA	NA	NA	NA	NA	NA	NA	NA	NA	NA	26	NA	NA	NA	NA	26	NA	NA	26	183
189	Hoang, V. T., Ali-Salem, S. et al.	2019	Respiratory tract infections among French Hajj pilgrims from 2014 to 2017	Scientific Reports	9	1	NA	Cross-sectional studies	2014-01-01	2017-12-31	France	81	390	NA	NA	NA	NA	390	NA	NA	390	475	NA	NA	NA	NA	NA	NA	NA	NA	NA	NA
190	Hoang, V. T., Dao, T. L. et al.	2019	The dynamics and interactions of respiratory pathogen carriage among French pilgrims during the 2018 Hajj	Emerging Microbes & Infections	8	1	1701-1710	Cross-sectional studies	2018-08-19	2018-08-24	France	100	113	NA	NA	NA	NA	113	NA	NA	113	121	378	NA	NA	NA	NA	378	NA	NA	378	484
191	Hoang, V. T., Goumballa, N. et al.	2019	Respiratory and gastrointestinal infections at the 2017 Grand Magal de Touba, Senegal: A prospective cohort survey	Travel Medicine & Infectious Disease	32	NA	101410	Cross-sectional studies	2017-11-04	2017-11-23	Senegal	75	46	NA	NA	NA	NA	46	NA	NA	46	110	NA	NA	NA	NA	NA	NA	NA	NA	NA	NA
192	Hoang, V. T., Meftah, M. et al.	2018	Bacterial respiratory carriage in French Hajj pilgrims and the effect of pneumococcal vaccine and other individual preventive measures: A prospective cohort survey	Travel Medicine & Infectious Disease	31	NA	NA	Cross-sectional studies	2015-09-21	2015-09-26	France	100	91	NA	NA	NA	NA	91	NA	NA	91	119	NA	NA	NA	NA	NA	NA	NA	NA	NA	NA
193	Hoang, V. T., Nguyen, T. T. et al.	2019	Antibiotic use for respiratory infections among Hajj pilgrims: A cohort survey and review of the literature	Travel Medicine & Infectious Disease	30	NA	39-45	Cross-sectional studies	2012-01-01	2017-12-31	France	100	641	NA	NA	NA	NA	641	NA	NA	641	754	NA	NA	NA	NA	NA	NA	NA	NA	NA	NA
194	Hochedez, P., Vinsentini, P. et al.	2006	Changes in the pattern of health disorders diagnosed among two cohorts of French travelers to Nepal, 17 years apart	Journal Of Travel Medicine	11	6	341-346	Cross-sectional studies	2001-01-01	2001-12-31	France	56	48	NA	NA	NA	NA	48	NA	NA	48	21457	NA	NA	NA	NA	NA	NA	NA	NA	NA	NA
195	Hoehl, S., Rabenau, H. et al.	2020	Evidence of SARS-CoV-2 Infection in Returning Travelers from Wuhan, China	New England Journal Of Medicine	382	13	1278-1280	Cross-sectional studies	2020-02-01	NA	Germany	62	NA	NA	NA	NA	NA	NA	NA	NA	NA	NA	2	NA	NA	NA	NA	2	NA	NA	2	114
196	Holshue, M. L., Debolt, C. et al.	2020	First Case of 2019 Novel Coronavirus in the United States	New England Journal Of Medicine	382	10	929-936	Case report	2020-01-19	2020-01-30	United States	100	1	NA	1	NA	NA	NA	NA	1	NA	1	1	NA	1	NA	NA	NA	NA	1	NA	NA
197	Horvath, L. L., Murray, C. K. et al.	2006	Effect of maximizing a travel medicine clinic's prevention strategies	Journal Of Travel Medicine	12	6	332-337	Cross-sectional studies	2002-08-01	2003-08-01	United States	81	NA	NA	NA	NA	NA	NA	NA	NA	NA	NA	25	NA	NA	NA	NA	25	NA	NA	25	175
198	Hsueh, P. R., Chen, P. J. et al.	2004	Patient data, early SARS epidemic, Taiwan	Emerging Infectious Diseases	10	3	489-493	Case report	2003-03-08	2003-04-16	China	56	7	NA	NA	NA	NA	7	NA	NA	7	10	7	NA	NA	NA	NA	7	NA	NA	7	10
199	Husain, E. H., Dashti, A. A. et al.	2009	Absence of Neisseria meningitidis from Throat Swabs of Kuwaiti Pilgrims after Returning from the Hajj	Medical Principles & Practice	19	4	321-323	Cross-sectional studies	2005-01-18	2005-01-23	Kuwait	69	89	NA	NA	NA	NA	89	NA	NA	89	177	72	NA	NA	NA	NA	72	NA	NA	72	177
200	Hwang, M., Bae, J. M.	2021	Sources of Infection Among Confirmed Cases of COVID-19 in Jeju Province, Korea	Journal Of Preventive Medicine & Public Health	54	4	245-250	Cross-sectional studies	2020-01-20	2021-03-31	Democratic People's Republic of Korea	75	NA	NA	NA	NA	NA	NA	NA	NA	NA	NA	37	NA	NA	NA	NA	37	NA	NA	37	627

Study ID	Authors	Publication Year	Title	Journal	Volume	Issue	Pages	Study Design	Starting date	Ending date	Country of detection	JBI Critical Score (%)	Respiratory Symptoms										Respiratory Cases Reported									
													Total cases (n)	Age Distribution					Gender Distribution			Total population (N)	Total cases (n)	Age Distribution					Gender Distribution			Total population (N)
														Child/Young	Adult	Middle Age Adult	Senior Adult	Unknown Age	Female	Male	Unknown Gender			Child/Young	Adult	Middle Age Adult	Senior Adult	Unknown Age	Female	Male	Unknown Gender	
201	Imani, R., Karimi, A. et al.	2013	Acute respiratory viral infections among Tamattu'Hajj pilgrims in Iran	Life Science Journal	10	3	NA	Cross-sectional studies	2007-01-01	2008-12-31	Iran (Islamic Republic of)	44	284	NA	NA	NA	NA	284	NA	NA	284	338	NA	NA	NA	NA	NA	NA	NA	NA	NA	NA
202	Inglis, T. J. J., Spittle, C. et al.	2018	Legionnaires' Disease Outbreak on a Merchant Vessel, Indian Ocean, Australia, 2015	Emerging Infectious Diseases	24	7	1345-1348	Case series	2015-08-01	2015-08-31	Australia	60	3	NA	NA	3	NA	NA	NA	NA	3	3	3	NA	NA	3	NA	NA	NA	NA	3	3
203	Isea-Pena, M. C., Brezmes-Valdivieso, M. F. et al.	2012	Mycobacterium africanum, an emerging disease in high-income countries?	International Journal Of Tuberculosis & Lung Disease	16	10	1400-1404	Cross-sectional studies	2000-01-01	2010-12-31	Spain	75	NA	NA	NA	NA	NA	NA	NA	NA	NA	NA	46	NA	NA	NA	NA	46	NA	46	NA	57
204	Ishii, H., Asai, S. et al.	2005	Virus-associated hemophagocytic syndrome in an international traveler as a differential diagnosis of SARS	Internal Medicine Journal	44	4	342-345	Case report	2003-06-12	2003-06-16	Japan	100	1	NA	1	NA	NA	NA	1	NA	NA	1	1	NA	1	NA	NA	NA	1	NA	NA	1
205	Islamoglu, M. S., Cengiz, M. et al.	2021	COVID-19 seroconversion in the aircrew from Turkey	Travel Medicine & Infectious Disease	44	NA	NA	Cross-sectional studies	2020-12-01	2021-01-13	Turkey	81	NA	NA	NA	NA	NA	NA	NA	NA	NA	NA	122	NA	NA	NA	NA	122	NA	NA	122	313
206	Jansen, L., Tegomoh, B. et al.	2021	Investigation of a SARS-CoV-2 B.1.1.529 (Omicron) Variant Cluster - Nebraska, November-December 2021	Morbidity & Mortality Weekly Report	70	5152	1782-1784	Case report	2021-11-20	2021-11-26	United States	31	1	NA	NA	NA	NA	1	NA	NA	1	1	1	NA	NA	NA	NA	1	NA	NA	1	1
207	Jaureguiberry, S., Boutolleau, D. et al.	2011	Clinical and Microbiological Evaluation of Travel-Associated Respiratory Tract Infections in Travelers Returning From Countries Affected by Pandemic A(H1N1) 2009 Influenza	Journal Of Travel Medicine	19	1	22-27	Cross-sectional studies	2009-04-01	2009-07-30	France	75	NA	NA	NA	NA	NA	NA	NA	NA	NA	NA	99	NA	NA	NA	NA	99	NA	NA	99	113
208	Jennings, L. C., Priest, P. C. et al.	2015	Respiratory viruses in airline travellers with influenza symptoms: Results of an airport screening study	Journal Of Clinical Virology	67	NA	8-13	Cross-sectional studies	2008-06-23	2008-09-12	New Zealand	100	NA	NA	NA	NA	NA	NA	NA	NA	NA	NA	342	NA	NA	NA	NA	342	NA	NA	342	1313
209	Jones, G., Haeghebaert, S. et al.	2016	Measles outbreak in a refugee settlement in Calais, France: January to February 2016	Eurosurveillance	21	11	NA	Case series	2016-01-01	2016-02-28	France	80	NA	NA	NA	NA	NA	NA	NA	NA	NA	NA	9	NA	NA	NA	NA	9	NA	NA	9	13
210	Joseph, C. A.	2003	Legionnaires' disease in Europe 2000-2002	Epidemiology & Infection	132	3	417-424	Cross-sectional studies	2000-01-01	2002-12-31	UK	62	NA	NA	NA	NA	NA	NA	NA	NA	NA	NA	1424	NA	NA	NA	NA	1424	NA	NA	1424	10322
211	Joseph, C. A., Ricketts, K. D.	2010	Legionnaires' disease in Europe 2007-2008	Eurosurveillance	15	8	1-8	Prevalence studies	2007-01-01	2008-12-31	UK	62	NA	NA	NA	NA	NA	NA	NA	NA	NA	NA	1480	NA	NA	NA	NA	1480	NA	NA	1480	11867
212	Joseph, C. A., Ricketts, K. D. et al.	2010	Travel-associated Legionnaires disease in Europe in 2009	Eurosurveillance	15	41	NA	Cross-sectional studies	2009-01-01	2009-12-31	Several	75	NA	NA	NA	NA	NA	NA	NA	NA	NA	NA	818	NA	NA	NA	NA	818	NA	NA	818	818
213	Joseph, C., Van Wijngaarden, J. et al.	2005	Cruise-ship-associated legionnaires disease, November 2003-may 2004	Morbidity & Mortality Weekly Report	54	45	1153-1155	Case series	2003-11-01	2004-05-31	United States	70	NA	NA	NA	NA	NA	NA	NA	NA	NA	NA	8	NA	1	3	4	NA	3	5	NA	8
214	Kakimoto, K., Kamiya, H. et al.	2020	Initial Investigation of Transmission of COVID-19 Among Crew Members During Quarantine of a Cruise Ship - Yokohama, Japan, February 2020	Morbidity & Mortality Weekly Report	69	11	312-313	Case series	2020-02-04	2020-02-12	Japan	70	NA	NA	NA	NA	NA	NA	NA	NA	NA	NA	20	NA	NA	NA	NA	20	NA	NA	20	1068
215	Kapoor, M., Pringle, K. et al.	2014	Clinical and Laboratory Findings of the First Imported Case of Middle East Respiratory Syndrome Coronavirus to the United States	Clinical Infectious Diseases	59	11	1511-1518	Case report	2014-04-18	NA	United States	75	1	NA	NA	NA	1	NA	NA	1	NA	NA	1	NA	NA	NA	1	NA	NA	1	NA	1
216	Khamis, N. K.	2008	Epidemiological pattern of diseases and risk behaviors of pilgrims attending mina hospitals, hajj 1427 h (2007 g)	Journal Of The Egyptian Public Health Association	83	1	15-33	Cross-sectional studies	2007-12-17	2007-12-22	Saudi Arabia	88	NA	NA	NA	NA	NA	NA	NA	NA	NA	248	74	NA	NA	NA	NA	74	NA	NA	74	248
217	Khan, I. D., Khan, S. A. et al.	2017	Morbidity and mortality amongst Indian Hajj pilgrims: A 3-year experience of Indian Hajj medical mission in mass-gathering medicine	Journal Of Infection & Public Health	11	2	165-170	Cross-sectional studies	2014-01-01	2016-12-31	Saudi Arabia	75	NA	NA	NA	NA	NA	NA	NA	NA	NA	NA	616031	NA	NA	NA	NA	616031	NA	NA	616031	1215817
218	Khan, M., Khan, H. et al.	2020	Epidemiological and clinical characteristics of coronavirus disease (COVID-19) cases at a screening clinic during the early outbreak period: a single-centre study	Journal Of Medical Microbiology	69	8	1114-1123	Cross-sectional studies	2020-03-15	2020-04-21	Pakistan	75	NA	NA	NA	NA	NA	NA	NA	NA	NA	NA	22	NA	5	12	5	22	7	15	NA	121
219	Kim, J. H., Lee, D. H. et al.	2010	In-Flight Transmission of Novel Influenza A (H1N1)	Epidemiology & Health	32	NA	e2010006	Case series	2009-04-17	2009-05-03	Democratic People's Republic of Korea	80	5	NA	NA	1	1	NA	2	NA	NA	338	2	NA	NA	1	1	NA	2	NA	NA	199
220	Kobayashi, A., Yamamoto, Y. et al.	2004	Severe Legionella pneumophila pneumonia associated with the public bath on a cruise ship in Japan	Journal Of Anesthesia	18	2	129-131	Case report	NA	NA	Japan	62	1	NA	NA	NA	1	NA	NA	1	NA	1	1	NA	NA	NA	1	NA	NA	1	NA	1

Study ID	Authors	Publication Year	Title	Journal	Volume	Issue	Pages	Study Design	Starting date	Ending date	Country of detection	JBI Critical Score (%)	Respiratory Symptoms										Respiratory Cases Reported									
													Total cases (n)	Age Distribution					Gender Distribution			Total population (N)	Total cases (n)	Age Distribution					Gender Distribution			Total population (N)
														Child/Young	Adult	Middle Age Adult	Senior Adult	Unknown Age	Female	Male	Unknown Gender			Child/Young	Adult	Middle Age Adult	Senior Adult	Unknown Age	Female	Male	Unknown Gender	
221	Kocazeybek, B. S., Yuksel, P. et al.	2016	Travel-associated infections caused by unusual serogroups of Legionella pneumophila identified using Legionella BIOCHIP slides in Turkey and Iraq	Travel Medicine & Infectious Disease	14	3	248-254	Case series	2014-01-10	2014-02-19	Turkey	75	NA	NA	NA	NA	NA	NA	NA	NA	NA	NA	2	NA	NA	2	NA	NA	1	1	NA	50
222	Kofman, A., Eggers, P. et al.	2018	Contact Tracing Investigation after First Case of Andes Virus in the United States - Delaware, February 2018	Morbidity & Mortality Weekly Report	67	41	1162-1163	Case series	2018-01-20	2018-02-28	United States	75	1	NA	NA	NA	NA	1	1	NA	NA	1	1	NA	NA	NA	NA	1	1	NA	NA	1
223	Kofman, A., Rahav, G. et al.	2018	Exported Case of Sin Nombre Hantavirus Pulmonary Syndrome - Israel, 2017	Morbidity & Mortality Weekly Report	67	40	1129-1129	Case report	2017-10-03	2017-10-09	Israel	88	1	NA	NA	1	NA	NA	NA	1	NA	1	1	NA	NA	1	NA	NA	NA	1	NA	1
224	Korea Centers For Disease Control Prevention	2020	Early Trend of Imported COVID-19 Cases in South Korea	Osong Public Health & Research Perspectives	11	3	140-145	Cross-sectional studies	2020-01-20	2020-03-23	Democratic People's Republic of Korea	69	NA	NA	NA	NA	NA	NA	NA	NA	NA	NA	171	10	116	33	12	171	NA	NA	171	171
225	Koul, P. A., Mir, H. et al.	2016	Influenza not MERS CoV among returning Hajj and Umrah pilgrims with respiratory illness, Kashmir, north India, 2014–15	Travel Medicine & Infectious Disease	15	NA	45-47	Cross-sectional studies	2014-10-01	2015-04-30	India	69	NA	NA	NA	NA	NA	NA	NA	NA	NA	300	33	NA	NA	NA	NA	33	NA	NA	33	300
226	Kraaij Dirkzwager, M., Timen, A. et al.	2014	Middle East respiratory syndrome coronavirus (MERS-CoV) infections in two returning travellers in the Netherlands, May 2014	Eurosurveillance	19	21	NA	Case report	2014-04-26	2014-05-15	Netherlands	75	2	NA	NA	NA	2	NA	1	1	NA	2	2	NA	NA	NA	2	NA	1	1	NA	2
227	Kreidl, P., Schmid, D. et al.	2020	Emergence of coronavirus disease 2019 (COVID-19) in Austria	Wiener Klinische Wochenschrift	132	21	645-652	Case series	2020-01-20	2020-03-31	Austria	65	NA	NA	NA	NA	NA	NA	NA	NA	NA	NA	3	NA	1	1	1	NA	NA	2	1	3
228	Kuenzli, A. B., Marschall, J. et al.	2018	Hantavirus Cardiopulmonary Syndrome Due to Imported Andes Hantavirus Infection in Switzerland: A Multidisciplinary Challenge, Two Cases and a Literature Review	Clinical Infectious Diseases	67	11	1788-1795	Case report	2016-09-01	2016-11-30	Switzerland	94	2	NA	NA	2	NA	NA	1	1	NA	2	2	NA	NA	2	NA	NA	1	1	NA	2
229	Kutsuna, S., Asai, Y. et al.	2020	Epidemiological trends of imported infectious diseases in Japan: Analysis of imported 2-year infectious disease registry data	Journal Of Infection & Chemotherapy	27	4	632-638	Prevalence studies	2017-10-01	2019-09-30	Japan	75	NA	NA	NA	NA	NA	NA	NA	NA	NA	NA	678	NA	NA	NA	NA	678	NA	NA	678	3046
230	Lago, K., Telu, K. et al.	2020	Impact of Doxycycline as Malaria Prophylaxis on Risk of Influenza-Like Illness among International Travelers	American Journal Of Tropical Medicine & Hygiene	102	4	821-826	Cross-sectional studies	NA	NA	United States	94	NA	NA	NA	NA	NA	NA	NA	NA	NA	3227	NA	NA	NA	NA	NA	NA	NA	NA	NA	NA
231	Lau, H., Khosrawipour, V. et al.	2020	Internationally lost COVID-19 cases	Journal Of Microbiology Immunology & Infection	53	3	454-458	Cross-sectional studies	2020-01-20	2020-02-18	Several	62	NA	NA	NA	NA	NA	NA	NA	NA	NA	NA	173	NA	NA	NA	NA	173	NA	NA	173	337
232	Lau, S. K., Chan, J. F. et al.	2016	Middle East Respiratory Syndrome Interpreted: Human Herpesvirus 6B Pneumonia	American Journal Of Medicine	129	9	945-7	Case report	NA	NA	China, Hong Kong Special Administrative Region	75	1	NA	NA	1	NA	NA	NA	1	NA	1	1	NA	NA	1	NA	NA	NA	1	NA	1
233	Leder, K., Sundararajan, V. et al.	2003	Respiratory Tract Infections in Travelers: A Review of the GeoSentinel Surveillance Network	Clinical Infectious Diseases	36	4	399-406	Cross-sectional studies	1997-11-01	2001-09-30	Several	88	NA	NA	NA	NA	NA	NA	NA	NA	NA	NA	1719	NA	NA	NA	NA	1719	874	830	15	21960
234	Leder, K., Tong, S. et al.	2006	Illness in travelers visiting friends and relatives: A review of the GeoSentinel Surveillance Network	Clinical Infectious Diseases	43	9	1185-1193	Cross-sectional studies	1997-11-01	2004-12-01	Several	100	NA	NA	NA	NA	NA	NA	NA	NA	NA	NA	576	NA	NA	NA	NA	576	NA	NA	576	12504
235	Leder, K., Torresi, J. et al.	2015	GeoSentinel Surveillance of Illness in Returned Travelers, 2007–2011	Annals Of Internal Medicine	158	6	456-468	Cross-sectional studies	2007-01-01	2011-12-31	Several	88	NA	NA	NA	NA	NA	NA	NA	NA	NA	NA	4613	NA	NA	NA	NA	4613	2228	2385	NA	42173
236	Lee, J. J., Choe, Y. J. et al.	2021	Importation and Transmission of SARS-CoV-2 B.1.1.529 (Omicron) Variant of Concern in Korea, November 2021	Journal Of Korean Medical Science	36	50	e346	Case series	2021-11-24	2021-12-10	Democratic People's Republic of Korea	70	NA	NA	NA	NA	NA	NA	NA	NA	NA	NA	14	NA	NA	NA	NA	14	NA	NA	14	80
237	Lehman, B., Procop, G. W. et al.	2019	Chronic laryngitis caused by Mycobacterium kansasii in a traveler	Laryngoscope	129	11	2534-2536	Case report	2012-01-01	2017-12-31	United States	94	1	NA	NA	1	NA	NA	1	NA	NA	1	1	NA	NA	1	NA	NA	1	NA	NA	1
238	Leroy, H., Arvieux, C. et al.	2008	A retrospective study of 230 consecutive patients hospitalized for presumed travel-related illness (2000–2006)	European Journal Of Clinical Microbiology & Infectious Diseases	27	11	1137-1140	Cross-sectional studies	2000-01-01	2006-03-31	France	75	NA	NA	NA	NA	NA	NA	NA	NA	NA	NA	46	NA	NA	NA	NA	47	NA	NA	47	230
239	Leung, G. M., Hedley, A. J. et al.	2004	The Epidemiology of Severe Acute Respiratory Syndrome in the 2003 Hong Kong Epidemic: An Analysis of All 1755 Patients	Annals Of Internal Medicine	141	9	662	Cross-sectional studies	2003-01-01	2003-12-31	China	94	NA	NA	NA	NA	NA	NA	NA	NA	NA	NA	79	NA	NA	NA	NA	79	NA	NA	79	1755
240	Leung, W. L. H., Yu, E. L. M. et al.	2021	Findings from the first public COVID-19 temporary test centre in Hong Kong	Hong Kong Medical Journal	27	2	99-105	Cross-sectional studies	2020-01-01	2020-04-30	China	75	NA	NA	NA	NA	NA	NA	NA	NA	NA	NA	86	2	NA	NA	1	83	40	46	NA	1258

Study ID	Authors	Publication Year	Title	Journal	Volume	Issue	Pages	Study Design	Starting date	Ending date	Country of detection	JBI Critical Score (%)	Respiratory Symptoms										Respiratory Cases Reported									
													Total cases (n)	Age Distribution					Gender Distribution			Total population (N)	Total cases (n)	Age Distribution					Gender Distribution			Total population (N)
														Child/Young	Adult	Middle Age Adult	Senior Adult	Unknown Age	Female	Male	Unknown Gender			Child/Young	Adult	Middle Age Adult	Senior Adult	Unknown Age	Female	Male	Unknown Gender	
241	Leutscher, P. D. C., Bagley, S. W.	2006	Health-related challenges in United States Peace Corps Volunteers serving for two years in Madagascar	Journal Of Travel Medicine	10	5	263-267	Cross-sectional studies	2001-01-01	2001-12-31	Madagascar	69	95	NA	NA	NA	NA	95	NA	NA	95	640	NA	NA	NA	NA	NA	NA	NA	NA	NA	NA
242	Li, L., Ross, D. et al.	2020	Two cases of imported respiratory diphtheria in Edinburgh, Scotland, October 2019	Epidemiology & Infection	148	NA	NA	Case report	2019-10-18	2019-11-22	United Kingdom of Great Britain and Northern Ireland	94	2	NA	NA	2	NA	NA	1	1	NA	2	2	NA	NA	2	NA	NA	1	1	NA	2
243	Liang, G., Shen, Y. et al.	2018	Coccidioidomycosis: Imported and possible domestic cases in China: A case report and review, 1958-2017	Mycoses	61	7	506-513	Case report	2014-04-01	2016-09-30	China	94	1	NA	1	NA	NA	NA	NA	1	NA	1	1	NA	1	NA	NA	NA	NA	1	NA	1
244	Lie, J., Maxwell, S. et al.	2009	A case of pulmonary melioidosis	Respiratory Medicine Cme	3	2	109-110	Case report	NA	NA	UK	75	1	NA	NA	1	NA	NA	NA	1	NA	1	1	NA	NA	1	NA	NA	NA	1	NA	1
245	Liu, J., Huang, J. et al.	2020	Large SARS-CoV-2 Outbreak Caused by Asymptomatic Traveler, China	Emerging Infectious Diseases	26	9	2260-2263	Case report	2020-03-19	2020-04-23	China	50	NA	NA	NA	NA	NA	NA	NA	NA	NA	NA	1	NA	NA	NA	NA	1	1	NA	NA	1
246	Liu, J., Liu, S. et al.	2020	Epidemiology, clinical characteristics of the first cases of COVID-19	European Journal Of Clinical Investigation	50	10	NA	Case series	2019-12-01	2020-07-09	Several	75	NA	NA	NA	NA	NA	NA	NA	NA	NA	NA	16	NA	9	4	3	NA	8	8	NA	16
247	Liu, W., Hu, W. et al.	2021	Travel-related infection in Guangzhou, China,2009–2019	Travel Medicine & Infectious Disease	43	NA	NA	Cross-sectional studies	2009-01-01	2019-12-31	China	69	NA	NA	NA	NA	NA	NA	NA	NA	NA	NA	2	NA	NA	NA	NA	2	NA	NA	2	1478
248	Loncarevic, G., Payne, L. et al.	2009	Public health preparedness for two mass gathering events in the context of pandemic influenza (H1N1) 2009 - Serbia, July 2009	Eurosurveillance	14	31	NA	Cross-sectional studies	2009-07-01	2009-07-31	Serbia	62	NA	NA	NA	NA	NA	NA	NA	NA	NA	NA	69	NA	NA	NA	NA	69	NA	NA	69	69
249	Louie, T., Kwan, B. et al.	2021	Respiratory failure, clinical course and community management of COVID-19 patients in a large Australian cohort	Internal Medicine Journal	51	3	334-340	Cross-sectional studies	2020-03-19	2020-05-15	Australia	94	NA	NA	NA	NA	NA	NA	NA	NA	NA	NA	58	NA	NA	NA	NA	58	NA	NA	58	99
250	Lytras, T., Dellis, G. et al.	2020	High prevalence of SARS-CoV-2 infection in repatriation flights to Greece from three European countries	Journal Of Travel Medicine	27	3	NA	Cross-sectional studies	2020-03-20	2020-03-25	Greece	62	NA	NA	NA	NA	NA	NA	NA	NA	NA	NA	40	NA	NA	NA	NA	40	NA	NA	40	783
251	Ma, X., Liu, F. et al.	2017	No MERS-CoV but positive influenza viruses in returning Hajj pilgrims, China, 20132015	BMC Infectious Diseases	17	1	715	Cross-sectional studies	2013-01-01	2015-12-31	China	75	NA	NA	NA	NA	NA	NA	NA	NA	NA	847	57	NA	NA	NA	NA	57	NA	NA	57	847
252	Madamar, Awala, P. S. et al.	2020	Is International Travel an Emerging Issue on Transmission of Beijing Lineage Mycobacterium tuberculosis?	Journal Of Tropical Medicine	2020	NA	NA	Cohort studies	2018-02-01	2019-09-30	Sri Lanka	77	NA	NA	NA	NA	NA	NA	NA	NA	NA	NA	13	NA	NA	NA	NA	13	NA	NA	13	94
253	Madani, T. A., Ghabrah, T. M. et al.	2007	Causes of admission to intensive care units in the Hajj period of the Islamic Year 1424 (2004)	Annals Of Saudi Medicine	27	2	101-105	Cross-sectional studies	2004-01-30	2004-02-04	Saudi Arabia	88	NA	NA	NA	NA	NA	NA	NA	NA	NA	NA	31	NA	NA	NA	NA	31	NA	NA	31	140
254	Madani, T. A., Ghabrah, T. M. et al.	2006	Causes of hospitalization of pilgrims during the Hajj period of the Islamic year 1423 (2003)	Annals Of Saudi Medicine	26	5	346-351	Cross-sectional studies	2003-02-08	2003-02-14	Saudi Arabia	81	NA	NA	NA	NA	NA	NA	NA	NA	NA	NA	9	NA	NA	NA	NA	9	NA	NA	9	808
255	Maechler, F., Gertler, M. et al.	2020	Epidemiological and clinical characteristics of SARS-CoV-2 infections at a testing site in Berlin, Germany, March and April 2020-a cross-sectional study	Clinical Microbiology & Infection	26	12	NA	Cross-sectional studies	2020-03-03	2020-04-13	Germany	94	NA	NA	NA	NA	NA	NA	NA	NA	NA	NA	71	NA	NA	NA	NA	71	NA	NA	71	796
256	Maggi, F., Novazzi, F. et al.	2021	Imported SARS-CoV-2 Variant P.1 in Traveler Returning from Brazil to Italy	Emerging Infectious Diseases	27	4	1249-1251	Case report	2020-11-23	2021-01-16	Italy	44	NA	NA	NA	NA	NA	NA	NA	NA	NA	NA	1	NA	1	NA	NA	NA	NA	1	NA	1
257	Mailles, A., Blanckaert, K. et al.	2013	First cases of Middle East Respiratory Syndrome Coronavirus (MERS-CoV) infections in France, investigations and implications for the prevention of human-to-human transmission, France, May 2013	Eurosurveillance	18	24	NA	Case report	2013-04-17	2013-05-28	France	88	1	NA	NA	NA	1	NA	NA	1	NA	1	1	NA	NA	NA	1	NA	NA	1	NA	1
258	Malhotra, S., Rahi, M. et al.	2021	Epidemiological profiles and associated risk factors of SARS-CoV-2 positive patients based on a high-throughput testing facility in India	Open Biology	11	6	NA	Cross-sectional studies	2020-04-06	2020-12-31	India	69	NA	NA	NA	NA	NA	NA	NA	NA	NA	NA	54	NA	NA	NA	NA	54	NA	NA	54	393
259	Mandourah, Y., Al-Radi, A. et al.	2012	Clinical and temporal patterns of severe pneumonia causing critical illness during Hajj	BMC Infectious Diseases	12	1	117	Cross-sectional studies	2009-01-01	2010-12-31	Saudi Arabia	81	NA	NA	NA	NA	NA	NA	NA	NA	NA	NA	123	NA	NA	NA	NA	123	NA	NA	123	123
260	Manuel Ramos, J., Masia, M. et al.	2011	Imported and non-imported diseases in the immigrant population. A decade of experience from an infectious diseases unit	Enfermedades Infecciosas Y Microbiologia Clinica	29	3	185-192	Cross-sectional studies	2001-06-01	2010-05-30	Spain	75	204	NA	NA	NA	NA	204	NA	NA	204	1071	475	NA	NA	NA	NA	475	NA	NA	475	1071

Study ID	Authors	Publication Year	Title	Journal	Volume	Issue	Pages	Study Design	Starting date	Ending date	Country of detection	JBI Critical Score (%)	Respiratory Symptoms										Respiratory Cases Reported									
													Total cases (n)	Age Distribution					Gender Distribution			Total population (N)	Total cases (n)	Age Distribution					Gender Distribution			Total population (N)
														Child/Young	Adult	Middle Age Adult	Senior Adult	Unknown Age	Female	Male	Unknown Gender			Child/Young	Adult	Middle Age Adult	Senior Adult	Unknown Age	Female	Male	Unknown Gender	
261	Marimoutou, C., Tufo, D. et al.	2017	Infection burden among medical events onboard cargo ships: a four-year study	Journal Of Travel Medicine	24	3	NA	Cross-sectional studies	2012-01-01	2015-12-31	Several	81	NA	NA	NA	NA	NA	NA	NA	NA	NA	NA	3	NA	NA	NA	NA	3	NA	3	NA	322
262	Marshall, C. A., Morris, E. et al.	2016	An epidemiological study of rates of illness in passengers and crew at a busy Caribbean cruise port	BMC Public Health	16	1	314	Cross-sectional studies	2009-01-01	2013-12-31	Barbados	62	NA	NA	NA	NA	NA	NA	NA	NA	NA	NA	50	NA	NA	NA	NA	50	NA	NA	50	4859682
263	Matsui, T., Nakamoto, T. et al.	2019	Case Report: Two Cases of Acute Q Fever from the Same Family Who Returned from Malawi to Japan	American Journal Of Tropical Medicine & Hygiene	101	6	1263-1264	Case report	2018-07-01	2018-07-31	Japan	75	2	1	NA	1	NA	NA	1	1	NA	2	2	1	NA	1	NA	NA	1	1	NA	2
264	Matsumura, K., Toyoda, Y. et al.	2020	Comparison of the Clinical Course of COVID-19 Pneumonia and Acute Respiratory Distress Syndrome in 2 Passengers from the Cruise Ship Diamond Princess in February 2020	American Journal Of Case Reports	21	NA	NA	Case report	NA	NA	Japan	81	2	NA	NA	NA	2	NA	NA	2	NA	2	2	NA	NA	NA	2	NA	NA	2	NA	2
265	Matteelli, A., Beltrame, A. et al.	2006	Respiratory syndrome and respiratory tract infections in foreign-born and national travelers hospitalized with fever in Italy	Journal Of Travel Medicine	12	4	190-196	Cross-sectional studies	1998-09-01	2000-12-31	Italy	100	NA	NA	NA	NA	NA	NA	NA	NA	NA	NA	40	NA	NA	NA	NA	40	NA	NA	40	515
266	May, M. L. A., Mcdougall, R. J. et al.	2013	Corynebacterium diphtheriae and the Returned Tropical Traveler	Journal Of Travel Medicine	21	1	39-44	Cross-sectional studies	2002-01-01	2012-12-31	Australia	81	NA	NA	NA	NA	NA	NA	NA	NA	NA	NA	44	NA	NA	NA	NA	44	12	32	NA	72
267	Mcgovern, O. L., Stenger, M. et al.	2021	Demographic, clinical, and epidemiologic characteristics of persons under investigation for Coronavirus Disease 2019-United States, January 17-February 29, 2020	PLOS One	16	4	NA	Cross-sectional studies	2020-01-17	2020-02-29	United States	75	NA	NA	NA	NA	NA	NA	NA	NA	NA	NA	19	NA	NA	NA	NA	19	NA	NA	19	36
268	Mckendrick, M.	2003	Infectious diseases and the returning traveller – experience from a regional infectious diseases unit over 20 years: INFECTION IN RETURNING TRAVELLERS	Journal Of Applied Microbiology	94	NA	25-30	Cross-sectional studies	1983-01-01	2002-12-31	UK	56	NA	NA	NA	NA	NA	NA	NA	NA	NA	NA	297	NA	NA	NA	NA	297	NA	NA	297	2202
269	Megan Lewis, Ruth Sanchez et al.	2020	COVID-19 Outbreak Among College Students After a Spring Break Trip to Mexico — Austin, Texas, March 26–April 5, 2020	Morbidity & Mortality Weekly Report	69	26	830-835	Cross-sectional studies	2020-03-27	2020-04-05	United States of America	94	89	NA	NA	NA	NA	89	NA	NA	89	183	60	NA	NA	NA	NA	60	NA	NA	NA	183
270	Memish, Z. A., Assiri, A. et al.	2015	Mass gathering and globalization of respiratory pathogens during the 2013 Hajj	Clinical Microbiology & Infection	21	6	571.e1-571.e8	Cross-sectional studies	2013-10-02	2013-10-24	Saudi Arabia	62	NA	NA	NA	NA	NA	NA	NA	NA	NA	NA	425	NA	NA	NA	NA	425	NA	NA	425	692
271	Memish, Z. A., Assiri, A. M. et al.	2001	Detection of Respiratory Viruses Among Pilgrims in Saudi Arabia During the Time of a Declared Influenza A(H1N1) Pandemic	Journal Of Travel Medicine	19	1	15-21	Cross-sectional studies	2009-09-25	2009-09-30	Saudi Arabia	94	NA	NA	NA	NA	NA	NA	NA	NA	NA	NA	400	NA	NA	NA	NA	400	NA	NA	400	3200
272	Mendelson, M., Han, P. V. et al.	2014	Regional Variation in Travel-related Illness acquired in Africa, March 1997–May 2011	Emerging Infectious Diseases	20	4	532-541	Cross-sectional studies	1997-03-01	2011-05-01	Several	94	NA	NA	NA	NA	NA	NA	NA	NA	NA	NA	120	NA	NA	NA	NA	120	NA	NA	120	16893
273	Merza, M. A., Al Mezori, A. A. H. et al.	2020	COVID-19 outbreak in Iraqi Kurdistan: The first report characterizing epidemiological, clinical, laboratory, and radiological findings of the disease	Diabetes & Metabolic Syndrome-Clinical Research & Reviews	14	4	547-554	Cross-sectional studies	2020-03-18	2020-04-07	Iraq	88	NA	NA	NA	NA	NA	NA	NA	NA	NA	NA	5	1	2	2	NA	NA	2	3	NA	15
274	Meyer, B., Basra, A. et al.	2015	MERS-COV disease associated ards -A case report	Critical Care Medicine	43	12	308	Case report	NA	NA	Austria	75	1	NA	1	NA	NA	NA	1	NA	NA	1	1	NA	1	NA	NA	NA	1	NA	NA	1
275	Meysamie, A., Ardakani, H. Z. et al.	2006	Comparison of mortality and morbidity rates among Iranian pilgrims in Hajj 2004 and 2005	Saudi Medical Journal	27	7	1049-1053	Cross-sectional studies	2004-01-01	2005-12-31	Saudi Arabia	81	63534	NA	NA	NA	NA	63534	NA	NA	63534	105713	18482	NA	NA	NA	NA	18482	NA	NA	18482	105713
276	Millman, A. J., Kornylo Duong, K. et al.	2015	Influenza Outbreaks among Passengers and Crew on Two Cruise Ships: a Recent Account of Preparedness and Response to an Ever-Present Challenge	Journal Of Travel Medicine	22	5	306-311	Cross-sectional studies	2014-03-15	2014-04-05	United States	100	NA	NA	NA	NA	NA	NA	NA	NA	NA	NA	46	NA	NA	NA	NA	46	NA	NA	46	7796
277	Moattari, A., Emami, A. et al.	2012	Influenza viral infections among the Iranian Hajj pilgrims returning to Shiraz, Fars province, Iran: Influenza infections of Hajj pilgrims in Iran	Influenza & Other Respiratory Viruses	6	6	e77-e79	Cross-sectional studies	2009-12-15	2009-12-21	Iran (Islamic Republic of)	62	NA	NA	NA	NA	NA	NA	NA	NA	NA	NA	33	NA	NA	NA	NA	33	NA	NA	33	275
278	Monge-Maillo, B., Norman, F.f. et al.	2013	Travelers visiting friends and relatives (VFR) and imported infectious disease: Travelers, immigrants or both? A comparative analysis	Travel Medicine & Infectious Disease	12	1	88-94	Cross-sectional studies	1989-04-01	2010-06-30	Spain	69	22	NA	NA	NA	NA	22	NA	NA	22	351	68	NA	NA	NA	NA	68	NA	NA	68	351

Study ID	Authors	Publication Year	Title	Journal	Volume	Issue	Pages	Study Design	Starting date	Ending date	Country of detection	JBI Critical Score (%)	Respiratory Symptoms										Respiratory Cases Reported									
													Total cases (n)	Age Distribution					Gender Distribution			Total population (N)	Total cases (n)	Age Distribution					Gender Distribution			Total population (N)
														Child/Young	Adult	Middle Age Adult	Senior Adult	Unknown Age	Female	Male	Unknown Gender			Child/Young	Adult	Middle Age Adult	Senior Adult	Unknown Age	Female	Male	Unknown Gender	
279	Morgan, J., Cano, M. V. et al.	2003	A large outbreak of histoplasmosis among American travelers associated with a hotel in Acapulco, Mexico, spring 2001	American Journal Of Tropical Medicine & Hygiene	69	6	663-669	Cross-sectional studies	2001-03-01	2001-05-31	United States	100	262	NA	NA	NA	NA	262	156	106	NA	502	75	NA	NA	NA	NA	75	NA	NA	75	112
280	Moriarty, L. F., Plucinski, M. M. et al.	2020	Public Health Responses to COVID-19 Outbreaks on Cruise Ships — Worldwide, February–March 2020	Morbidity & Mortality Weekly Report	69	12	347-352	Prevalence studies	2002-02-01	2020-03-31	Several	78	NA	NA	NA	NA	NA	NA	NA	NA	NA	NA	790	NA	NA	NA	NA	790	NA	NA	790	4180
281	Mukherjee, P., Lim, P. L. et al.	2010	Epidemiology of Travel-associated Pandemic (H1N1) 2009 Infection in 116 Patients, Singapore	Emerging Infectious Diseases	16	1	21-26	Cross-sectional studies	2009-04-27	2009-06-27	Singapore	88	NA	NA	NA	NA	NA	NA	NA	NA	NA	NA	116	NA	NA	NA	NA	116	NA	NA	116	152
282	Murphy, N., Bol, M. et al.	2020	A large national outbreak of COVID-19 linked to air travel, Ireland, summer 2020	Eurosurveillance	25	42	12-17	Case series	NA	NA	Ireland	70	NA	NA	NA	NA	NA	NA	NA	NA	NA	NA	13	NA	NA	NA	NA	13	NA	NA	13	49
283	Mushi, A., Yassin, Y. et al.	2021	A Longitudinal Study Regarding the Health Profile of the 2017 South African Hajj Pilgrims	International Journal Of Environmental Research & Public Health	18	7	NA	Cross-sectional studies	2017-10-13	2018-02-05	South Africa	75	413	NA	NA	NA	NA	413	NA	NA	413	1138	375	NA	NA	NA	NA	375	NA	NA	375	1138
284	Mustafa, A. N., Gessner, B. D. et al.	2003	A case-control study of influenza vaccine effectiveness among Malaysian pilgrims attending the Haj in Saudi Arabia	International Journal Of Infectious Diseases	7	3	210-214	Case control	2000-02-15	2000-03-14	Malaysia	100	1310	NA	NA	NA	NA	1310	NA	NA	1310	1310	NA	NA	NA	NA	NA	NA	NA	NA	NA	NA
285	Mutsch, M., Tavernini, M. et al.	2005	Influenza Virus Infection in Travelers to Tropical and Subtropical Countries	Clinical Infectious Diseases	40	9	1282-1287	Cross-sectional studies	1998-01-01	2000-03-31	Switzerland	100	NA	NA	NA	NA	NA	NA	NA	NA	NA	1450	40	NA	NA	NA	NA	40	16	24	NA	1450
286	Myers, J. F., Snyder, R. E. et al.	2020	Identification and Monitoring of International Travelers During the Initial Phase of an Outbreak of COVID-19-California, February 3-March 17, 2020	Morbidity & Mortality Weekly Report	69	19	599-602	Cross-sectional studies	2020-03-03	2020-03-17	United States	56	NA	NA	NA	NA	NA	NA	NA	NA	NA	NA	3	NA	NA	NA	NA	3	NA	NA	3	11574
287	Neatherlin, J., Cramer, E. H. et al.	2012	Influenza A(H1N1)pdm09 during air travel	Travel Medicine & Infectious Disease	11	2	110-118	Cross-sectional studies	2020-04-21	2020-05-20	United States	75	15	NA	NA	NA	NA	15	NA	NA	15	277	4	NA	NA	NA	NA	4	NA	NA	4	43
288	Neghina, A. M., Marincu, I. et al.	2010	Occurrence of Influenza A(H1N1)v Virus in Western Romania in Relationship to International Travel	Vector-Borne & Zoonotic Diseases	10	9	935-938	Case series	2009-06-15	2009-07-15	Roumania	85	1	NA	1	NA	NA	NA	NA	1	NA	1	2	NA	2	NA	NA	NA	1	1	NA	2
289	Nguyen Cong, K., Pham Quang, T. et al.	2020	Transmission of SARS-CoV 2 During Long-Haul Flight	Emerging Infectious Diseases	26	11	2617-2624	Cross-sectional studies	2020-03-02	2020-02-03	Vietnam	88	NA	NA	NA	NA	NA	NA	NA	NA	NA	NA	16	NA	NA	NA	9	7	7	9	NA	217
290	Nguyen, T. T., Pham, T. N. et al.	2020	Genetic diversity of SARS-CoV-2 and clinical, epidemiological characteristics of COVID-19 patients in Hanoi, Vietnam	PLOS One	15	11	e0242537	Cross-sectional studies	2020-03-06	2020-04-15	Vietnam	81	NA	NA	NA	NA	NA	NA	NA	NA	NA	NA	25	NA	NA	NA	NA	25	NA	NA	25	44
291	Nik Zuraina, N. M. N., Sarimah, A. et al.	2018	High frequency of Haemophilus influenzae associated with respiratory tract infections among Malaysian Hajj pilgrims	Journal Of Infection & Public Health	11	6	878-883	Cross-sectional studies	2016-09-27	2016-10-29	Malaysia	62	NA	NA	NA	NA	NA	NA	NA	NA	NA	NA	173	NA	NA	NA	NA	173	NA	NA	173	297
292	Obrien, D. P., Leder, K. et al.	2006	Illness in Returned Travelers and Immigrants/Refugees: The 6Year Experience of Two Australian Infectious Diseases Units	Journal Of Travel Medicine	13	3	145-152	Cross-sectional studies	1998-07-01	2004-09-30	Australia	75	210	NA	NA	NA	NA	210	NA	NA	210	1106	255	NA	NA	NA	NA	255	NA	NA	255	1106
293	Odolini, S., Parola, P. et al.	2012	Travel-related imported infections in Europe, EuroTravNet 2009	Clinical Microbiology & Infection	18	5	468-474	Cross-sectional studies	2008-01-01	2009-12-31	Several	100	NA	NA	NA	NA	NA	NA	NA	NA	NA	13349	NA	NA	NA	NA	NA	NA	NA	NA	NA	13349
294	Ojelade, M., Rodriguez, A. et al.	2021	Travel from the United Kingdom to the United States by a Symptomatic Patient Infected with the SARS-CoV-2 B.1.1.7 Variant-Texas, January 2021	Morbidity & Mortality Weekly Report	70	10	348-349	Case report	2021-01-10	2021-01-11	United States	81	NA	NA	NA	NA	NA	NA	NA	NA	NA	NA	1	NA	NA	NA	1	NA	NA	NA	1	1
295	Okada, P., Buathong, R. et al.	2020	Early transmission patterns of coronavirus disease 2019 (COVID-19) in travellers from Wuhan to Thailand, January 2020	Eurosurveillance	25	8	NA	Case report	2020-01-08	2020-01-18	China	81	2	NA	NA	NA	2	NA	2	NA	NA	2	2	NA	NA	NA	2	NA	2	NA	NA	2
296	Olanwijitwong, J., Piyaphanee, W. et al.	2017	Health problems among Thai tourists returning from India	Journal Of Travel Medicine	24	4	NA	Cross-sectional studies	2014-10-01	2015-03-31	Thailand	94	405	NA	NA	NA	NA	405	NA	NA	405	1304	NA	NA	NA	NA	NA	NA	NA	NA	NA	NA
297	Olsen, S. J., Chang, H. et al.	2003	Transmission of the severe acute respiratory syndrome on aircraft	New England Journal Of Medicine	349	25	2416-2422	Cross-sectional studies	2003-02-21	2003-03-21	China	62	NA	NA	NA	NA	NA	NA	NA	NA	NA	NA	23	NA	NA	NA	NA	23	NA	NA	23	681
298	Olsen, S. J., Chen, M. Y. et al.	2020	Early Introduction of Severe Acute Respiratory Syndrome Coronavirus 2 into Europe	Emerging Infectious Diseases	26	7	1567-1570	Case series	2020-01-01	2020-02-29	China	70	5	NA	NA	NA	NA	5	NA	NA	5	30	3	NA	NA	NA	NA	3	NA	NA	3	30
299	Ortiz, J. R., Wallis, T. R. et al.	2007	No Evidence of Avian Influenza A H5N1 among Returning US Travelers	Emerging Infectious Diseases	13	2	294-297	Cross-sectional studies	2003-02-01	2006-05-31	United States	62	NA	NA	NA	NA	NA	NA	NA	NA	NA	NA	27	NA	NA	NA	NA	27	NA	NA	27	59

Study ID	Authors	Publication Year	Title	Journal	Volume	Issue	Pages	Study Design	Starting date	Ending date	Country of detection	JBI Critical Score (%)	Respiratory Symptoms										Respiratory Cases Reported									
													Total cases (n)	Age Distribution					Gender Distribution			Total population (N)	Total cases (n)	Age Distribution					Gender Distribution			Total population (N)
														Child/Young	Adult	Middle Age Adult	Senior Adult	Unknown Age	Female	Male	Unknown Gender			Child/Young	Adult	Middle Age Adult	Senior Adult	Unknown Age	Female	Male	Unknown Gender	
300	Ospina, J. E., Orcau, N. et al.	2016	Epidemiology of Tuberculosis in Immigrants in a Large City with Large-Scale Immigration (1991-2013)	PLOS One	11	10	e0164736	Cross-sectional studies	1991-01-01	2013-12-31	Spain	100	NA	NA	NA	NA	NA	NA	NA	NA	NA	NA	3284	38	NA	NA	93	3150	1128	2156	NA	3284
301	Park, J. H., Jang, J. H. et al.	2020	COVID-19 Outbreak and Presymptomatic Transmission in Pilgrim Travelers Who Returned to Korea from Israel	Journal Of Korean Medical Science	35	48	NA	Cross-sectional studies	2020-02-21	2020-02-22	Democratic People's Republic of Korea	100	26	NA	NA	NA	NA	NA	NA	NA	NA	39	30	NA	NA	NA	9	21	22	8	NA	39
302	Parola, P., Renvoise, A. et al.	2012	Acanthamoeba polyphaga mimivirus Virophage Seroconversion in Travelers Returning from Laos	Emerging Infectious Diseases	18	9	1500-1502	Case report	2009-12-20	2010-01-22	France	75	NA	NA	NA	NA	NA	NA	NA	NA	NA	NA	2	NA	2	NA	NA	NA	1	1	NA	2
303	Parola, P., Soula, G. et al.	2005	Fever in travelers returning from tropical areas: prospective observational study of 613 cases hospitalised in Marseilles, France, 1999–2003	Travel Medicine & Infectious Disease	4	2	61-70	Cross-sectional studies	1999-01-01	2003-12-31	France	75	NA	NA	NA	NA	NA	NA	NA	NA	NA	NA	30	NA	NA	NA	NA	30	NA	NA	30	613
304	Payne, M., Skowronski, D. et al.	2018	Increase in Hospital Admissions for Severe Influenza A/B among Travelers on Cruise Ships to Alaska, 2015	Emerging Infectious Diseases	24	3	566-568	Case series	2015-06-01	2015-09-30	Canada	90	NA	NA	NA	NA	NA	NA	NA	NA	NA	NA	25	NA	NA	NA	NA	25	11	14	NA	25
305	Pebody, R. G., Chand , M. A. et al.	2012	The United Kingdom public health response to an imported laboratory confirmed case of a novel coronavirus in September 2012	Eurosurveillance	17	40	2-5	Case report	2012-07-31	2012-09-22	UK	62	1	NA	NA	1	NA	NA	NA	1	NA	1	1	NA	NA	1	NA	NA	NA	1	NA	1
306	Peck, A. J., Newbern, E. C. et al.	2004	Lack of SARS transmission and US SARS case-patient	Emerging Infectious Diseases	10	2	217-224	Case report	2003-03-29	2003-04-30	United States	94	1	NA	NA	1	NA	NA	NA	1	NA	1	1	NA	NA	1	NA	NA	NA	1	NA	1
307	Pey, P., Lee, K. et al.	2021	Health problems in travellers to Nepal visiting CIWEC clinic in Kathmandu — A GeoSentinel analysis	Travel Medicine & Infectious Disease	40	NA	NA	Cross-sectional studies	2009-01-01	2017-12-31	Nepal	81	NA	NA	NA	NA	NA	NA	NA	NA	NA	NA	5975	NA	NA	NA	NA	5975	NA	NA	5975	29281
308	Pfaff, G., Lohr, D. et al.	2010	Spotlight on measles 2010: Measles outbreak among travellers returning from a mass gathering, Germany, September to October 2010	Eurosurveillance	15	50	19750	Cross-sectional studies	2010-08-17	2010-09-19	Germany	62	NA	NA	NA	NA	NA	NA	NA	NA	NA	NA	13	8	1	NA	NA	4	10	3	NA	13
309	Pfefferle, S., Guenther, T. et al.	2020	SARS Coronavirus-2 variant tracing within the first Coronavirus Disease 19 clusters in northern Germany	Clinical Microbiology & Infection	27	1	NA	Case report	2020-02-22	2020-03-26	Germany	50	1	NA	NA	NA	NA	1	NA	1	NA	1	1	NA	NA	NA	NA	1	NA	1	NA	1
310	Pistone, T., Lacombe, K. et al.	2005	Imported concomitant coccidioidomycosis and histoplasmosis in an HIV-infected Colombian migrant in France	Transactions Of Royal Society Of Tropical Medicine & Hygiene	99	9	712-715	Case report	2001-07-03	2001-10-01	France	88	1	NA	NA	1	NA	NA	NA	1	NA	1	1	NA	NA	1	NA	NA	NA	1	NA	1
311	Pistone, T., Ouattara, E. et al.	2019	Travel-related health events and their risk factors in HIV-infected sub-Saharan migrants living in France and visiting their native country: The ANRS VIHVO cohort study	Travel Medicine & Infectious Disease	29	NA	40-47	Cross-sectional studies	2006-07-01	2009-06-01	France	100	NA	NA	NA	NA	NA	NA	NA	NA	NA	NA	20	NA	NA	NA	NA	20	NA	NA	20	268
312	Plucinski, M. M., Wallace, M. et al.	2020	Coronavirus Disease 2019 (COVID-19) in Americans Aboard the Diamond Princess Cruise Ship	Clinical Infectious Diseases	72	10	e448-e457	Cross-sectional studies	2020-01-20	2020-03-18	United States	100	NA	NA	NA	NA	NA	NA	NA	NA	NA	212	114	NA	NA	NA	NA	114	NA	NA	114	437
313	Potdar, V., Choudhary, M. L. et al.	2020	Respiratory virus detection among the overseas returnees during the early phase of COVID-19 pandemic in India	Indian Journal Of Medical Research	151	5	486-489	Cross-sectional studies	2020-01-22	2020-02-29	India	62	NA	NA	NA	NA	NA	NA	NA	NA	NA	NA	84	NA	NA	NA	NA	84	NA	NA	84	362
314	Poutanen, S. M., Low, D. E. et al.	2003	Identification of Severe Acute Respiratory Syndrome in Canada	New England Journal Of Medicine	348	20	1995-2005	Case series	2003-02-10	2003-03-20	Canada	90	2	NA	NA	1	1	NA	1	1	NA	10	3	NA	NA	NA	2	1	1	2	NA	10
315	Puca, E., Čivljak, R. et al.	2020	Short epidemiological overview of the current situation on COVID-19 pandemic in Southeast European (SEE) countries	Journal Of Infection In Developing Countries	14	5	433-437	Case series	2020-01-01	2020-04-14	Several	55	NA	NA	NA	NA	NA	NA	NA	NA	NA	NA	11	NA	NA	NA	NA	11	NA	NA	11	11
316	Qi, R. R., Wang, J. Q. et al.	2016	Descriptive epidemiology of deployment-related medical conditions and shipboard training-related injuries in a Chinese Navy population	Public Health	141	NA	170-177	Cross-sectional studies	2011-01-01	2015-12-31	China	75	NA	NA	NA	NA	NA	NA	NA	NA	NA	NA	76	NA	NA	NA	NA	76	NA	NA	76	1543
317	Rack, J., Wichmann, O. et al.	2006	Risk and spectrum of diseases in travelers to popular tourist destinations	Journal Of Travel Medicine	12	5	248-253	Cross-sectional studies	2003-07-01	2004-06-30	Germany	81	90	NA	NA	NA	NA	90	NA	NA	90	658	NA	NA	NA	NA	NA	NA	NA	NA	NA	NA
318	Rajabali, N., Lim, T. et al.	2015	Avian Influenza a (H5N1) Infection with Respiratory Failure and Meningoencephalitis in a Canadian Traveller	Canadian Journal Of Infectious Diseases & Medical Microbiology	26	4	221-223	Case report	2013-12-28	2014-01-02	Canada	88	1	NA	1	NA	NA	NA	1	NA	NA	1	1	NA	1	NA	NA	NA	1	NA	NA	1
319	Randhawa, H. S., Chowdhary, A. et al.	2013	Blastomycosis in India: report of an imported case and current status	Medical Mycology	51	2	185-192	Case report	2010-10-01	2012-01-30	India	100	1	NA	1	NA	NA	NA	1	NA	NA	1	1	NA	1	NA	NA	NA	1	NA	NA	1

Study ID	Authors	Publication Year	Title	Journal	Volume	Issue	Pages	Study Design	Starting date	Ending date	Country of detection	JBI Critical Score (%)	Respiratory Symptoms										Respiratory Cases Reported									
													Total cases (n)	Age Distribution					Gender Distribution			Total population (N)	Total cases (n)	Age Distribution					Gender Distribution			Total population (N)
														Child/Young	Adult	Middle Age Adult	Senior Adult	Unknown Age	Female	Male	Unknown Gender			Child/Young	Adult	Middle Age Adult	Senior Adult	Unknown Age	Female	Male	Unknown Gender	
320	Rashid, H., Barasheed, O. et al.	2013	Acute febrile respiratory infection symptoms in Australian Hajjis at risk of exposure to Middle East respiratory syndrome coronavirus	Medical Journal Of Australia	199	7	453-453	Prevalence studies	2013-10-23	2013-10-28	Australia	72	49	NA	NA	NA	NA	49	NA	NA	49	541	NA	NA	NA	NA	NA	NA	NA	NA	NA	NA
321	Rashid, H., Shafi, S. et al.	2008	Influenza and respiratory syncytial virus infections in British Hajj pilgrims	Emerging Health Threats Journal	2008	NA	NA	Cross-sectional studies	2005-01-18	2005-01-23	UK	75	205	NA	NA	NA	NA	205	NA	NA	205	205	37	NA	NA	NA	5	32	6	31	NA	202
322	Rashid, H., Shafi, S. et al.	2014	Viral respiratory infections at the Hajj: comparison between UK and Saudi pilgrims	Clinical Microbiology & Infection	14	6	569-574	Cross-sectional studies	2006-12-28	2007-01-02	UK	81	NA	NA	NA	NA	NA	NA	NA	NA	NA	260	NA	NA	NA	NA	NA	NA	NA	NA	NA	NA
323	Ratnam, I., Black, J. et al.	2013	Incidence and risk factors for acute respiratory illnesses and influenza virus infections in Australian travellers to Asia	Journal Of Clinical Virology	57	1	54-58	Cross-sectional studies	2007-08-01	2010-01-01	Australia	88	109	NA	NA	NA	NA	109	NA	NA	109	387	14	NA	NA	NA	NA	14	NA	NA	14	387
324	Ravensbergen, S. J., Berends, M. et al.	2017	High prevalence of MRSA and ESBL among asylum seekers in the Netherlands	PLOS One	12	4	e0176481	Cross-sectional studies	2014-01-01	2015-12-31	Netherlands	62	NA	NA	NA	NA	NA	NA	NA	NA	NA	NA	87	NA	NA	NA	NA	87	NA	NA	87	898
325	Razavi, S. M., Masoud, A. et al.	2014	Common infection and allergy induced biomarkers status in respiratory diseases among Iranian Hajj pilgrims	Health	6	1	137-140	Cross-sectional studies	NA	NA	Iran (Islamic Republic of)	56	118	NA	NA	NA	NA	118	NA	NA	118	130	NA	NA	NA	NA	NA	NA	NA	NA	NA	NA
326	Razavi, S. M., Sabouri-Kashani, A. et al.	2013	Trend of diseases among Iranian pilgrims during five consecutive years based on a Syndromic Surveillance System in Hajj	Medical Journal Of Islamic Republic Of Iran	27	4	179-185	Cross-sectional studies	2004-01-01	2008-12-31	Iran (Islamic Republic of)	50	NA	NA	NA	NA	NA	NA	NA	NA	NA	254823	NA	NA	NA	NA	NA	NA	NA	NA	NA	NA
327	Razavi, S.m., Ziaei, H. et al.	2007	Surveying respiratory infections among Iranian Hajj pilgrims	Life Science Journal	10	3	NA	Cross-sectional studies	NA	NA	Iran (Islamic Republic of)	62	NA	NA	NA	NA	NA	NA	NA	NA	NA	170	112	NA	NA	NA	NA	112	NA	NA	112	170
328	Redman, C. A., Maclennan, A. et al.	2006	Diarrhea and Respiratory Symptoms Among Travelers to Asia, Africa, and South and Central America From Scotland	Journal Of Travel Medicine	13	4	203-211	Cross-sectional studies	1997-01-01	2001-12-31	UK	88	337	44	128	53	29	79	195	142	NA	2006	NA	NA	NA	NA	NA	NA	NA	NA	NA	NA
329	Refaey, S., Amin, M. M. et al.	2016	Cross-sectional survey and surveillance for influenza viruses and MERS-CoV among Egyptian pilgrims returning from Hajj during 2012-2015	Influenza & Other Respiratory Viruses	11	1	57-60	Cross-sectional studies	2012-01-01	2015-12-31	Egypt	88	1170	NA	NA	NA	NA	1170	NA	NA	1170	3364	484	NA	NA	NA	NA	484	NA	NA	484	3364
330	Rha, B., Rudd, J. et al.	2015	Update on the epidemiology of Middle East respiratory syndrome coronavirus (MERS-CoV) infection, and guidance for the public, clinicians, and public health authorities - January 2015	Morbidity & Mortality Weekly Report	64	3	61-62	Case series	2014-08-01	2015-01-23	Several	45	NA	NA	NA	NA	NA	NA	NA	NA	NA	NA	3	NA	NA	NA	NA	3	NA	NA	3	102
331	Ricco, M., Peruzzi, S. et al.	2021	Epidemiology of Legionnaires' Disease in Italy, 2004-2019: A Summary of Available Evidence	Microorganisms	9	11	NA	Cross-sectional studies	2004-01-01	2020-12-31	Italy	100	NA	NA	NA	NA	NA	NA	NA	NA	NA	NA	2445	NA	NA	NA	NA	2445	NA	NA	2445	23554
332	Ricketts, K. D., Joseph, C. A. et al.	2007	Legionnaires' disease in Europe: 2005-2006	Eurosurveillance	12	12	7-8	Cross-sectional studies	2005-01-01	2006-12-31	Several	88	NA	NA	NA	NA	NA	NA	NA	NA	NA	NA	1395	NA	NA	NA	NA	1395	NA	NA	1395	11980
333	Ricketts, K. D., Joseph, C. et al.	2004	Travel Associated Legionnaires' Disease in Europe : 2003	Eurosurveillance	9	10	5-6	Cross-sectional studies	2003-01-01	2003-12-31	Several	62	NA	NA	NA	NA	NA	NA	NA	NA	NA	NA	NA	NA	NA	NA	NA	NA	NA	NA	NA	NA
334	Ripamonti, D., Barbo, R. et al.	2004	New times for an old disease: Intracranial mass lesions caused by Mycobacterium tuberculosis in 5 HIV-negative African immigrants	Clinical Infectious Diseases	39	5	E35-E45	Case series	1997-01-01	2003-06-30	Italy	100	NA	NA	NA	NA	NA	NA	NA	NA	NA	NA	5	NA	3	1	1	NA	2	3	NA	5
335	Roberts, M.t.m., Lever, A.m.l.	2003	An analysis of imported infections over a 5-year period at a teaching hospital in the United Kingdom	Travel Medicine & Infectious Disease	1	4	227-230	Cross-sectional studies	1998-01-01	2002-12-31	UK	62	NA	NA	NA	NA	NA	NA	NA	NA	NA	NA	19	NA	NA	NA	NA	19	NA	NA	19	301
336	Roed, T., Schonheyder, H. C. et al.	2015	Predictors of positive or negative legionella urinary antigen test in community-acquired pneumonia	Infectious Diseases	47	7	484-490	Cohort studies	2003-01-01	2013-12-31	Danemark	77	NA	NA	NA	NA	NA	NA	NA	NA	NA	NA	14	NA	NA	NA	NA	14	NA	NA	14	25
337	Ronald, L. A., Campbell, J. R. et al.	2018	Demographic predictors of active tuberculosis in people migrating to British Columbia, Canada: a retrospective cohort study	Canadian Medical Association Journal	190	8	E209-E216	Cross-sectional studies	1985-01-01	2012-12-31	Canada	100	NA	NA	NA	NA	NA	NA	NA	NA	NA	NA	362	NA	NA	NA	NA	362	NA	NA	362	2814
338	Roy, M., Benedict, K. et al.	2013	A Large Community Outbreak of Blastomycosis in Wisconsin With Geographic and Ethnic Clustering	Clinical Infectious Diseases	57	5	655-662	Cross-sectional studies	2005-01-01	2009-08-31	United States	100	NA	NA	NA	NA	NA	NA	NA	NA	NA	NA	48	NA	NA	NA	NA	48	NA	NA	48	108
339	Saidani, N., Griffiths, K. et al.	2015	Melioidosis as a travel-associated infection: Case report and review of the literature	Travel Medicine & Infectious Disease	13	5	367-381	Case series	1968-03-01	2014-12-31	Several	95	41	NA	NA	NA	NA	41	NA	NA	41	82	82	NA	NA	NA	NA	82	16	66	NA	82

Study ID	Authors	Publication Year	Title	Journal	Volume	Issue	Pages	Study Design	Starting date	Ending date	Country of detection	JBI Critical Score (%)	Respiratory Symptoms										Respiratory Cases Reported									
													Total cases (n)	Age Distribution					Gender Distribution			Total population (N)	Total cases (n)	Age Distribution					Gender Distribution			Total population (N)
														Child/Young	Adult	Middle Age Adult	Senior Adult	Unknown Age	Female	Male	Unknown Gender			Child/Young	Adult	Middle Age Adult	Senior Adult	Unknown Age	Female	Male	Unknown Gender	
340	Salva, E. P., Villarama, J. B. et al.	2020	Epidemiological and clinical characteristics of patients with suspected COVID-19 admitted in Metro Manila, Philippines	Tropical Medicine & Health	48	1	NA	Cross-sectional studies	2020-01-25	2020-03-29	Philippines	88	NA	NA	NA	NA	NA	NA	NA	NA	NA	NA	11	NA	NA	NA	NA	11	NA	NA	11	100
341	Salzer, H. J. F., Stoney, R. J. et al.	2018	Epidemiological aspects of travel-related systemic endemic mycoses: A GeoSentinel analysis, 1997-2017	Journal Of Travel Medicine	25	1	NA	Cross-sectional studies	1997-01-01	2017-12-31	Several	88	NA	NA	NA	NA	NA	NA	NA	NA	NA	NA	68	NA	NA	NA	NA	68	25	43	NA	68
342	Sanchez, S. M., Searle, E. F. et al.	2020	Travel-screening documentation to enable the "Identify-Isolate-Inform" framework for emerging infectious diseases: It's all in the details	Infection Control & Hospital Epidemiology	41	12	1449-1451	Cross-sectional studies	2018-08-01	2019-12-31	United States of America	56	NA	NA	NA	NA	NA	NA	NA	NA	NA	NA	NA	NA	NA	NA	NA	NA	NA	NA	NA	NA
343	Savini, H., Gautret, P. et al.	2013	Travel associated Diseases, Indian Ocean Islands, 1997-2010	Emerging Infectious Diseases	19	8	1297-1301	Cross-sectional studies	1997-01-01	2010-12-31	Several	81	NA	NA	NA	NA	NA	NA	NA	NA	NA	NA	102	NA	NA	NA	NA	102	NA	NA	102	1415
344	Savini, S., Monaco, D. et al.	2021	Prevention of the spread of SARS COV-2 by Rapid Antigenic Tests on the passengers entering an Italian seaport	Annali Di Igiene	33	5	518-520	Cross-sectional studies	2020-08-21	2020-09-27	Italy	56	NA	NA	NA	NA	NA	NA	NA	NA	NA	NA	212	NA	NA	NA	NA	212	NA	NA	212	38282
345	Scheifer, C., Rolland-Debord, C. et al.	2018	Re-emergence of Corynebacterium diphtheriae	Médecine & Maladies Infectieuses	49	6	463-466	Case report	2016-01-01	2016-12-31	France	75	NA	NA	NA	NA	NA	NA	NA	NA	NA	NA	2	NA	2	NA	NA	NA	NA	2	NA	2
346	Schlagenhauf, P., Chen, L. H. et al.	2010	Sex and Gender Differences in Travel-Associated Disease	Clinical Infectious Diseases	50	6	826-832	Cross-sectional studies	1997-03-01	2017-10-31	Several	88	NA	NA	NA	NA	NA	NA	NA	NA	NA	NA	6559	NA	NA	NA	NA	6559	3437	3122	NA	58908
347	Schlagenhauf, P., Loutan, L. et al.	2013	Acute and Potentially Life-Threatening Tropical Diseases in Western Travelers—A GeoSentinel Multicenter Study, 1996–2011	American Journal Of Tropical Medicine & Hygiene	88	2	397-404	Cross-sectional studies	1996-06-01	2011-08-31	Several	94	NA	NA	NA	NA	NA	NA	NA	NA	NA	NA	94	NA	NA	NA	NA	94	17	77	NA	820
348	Schlagenhauf, P., Weld, L. et al.	2014	Travel-associated infection presenting in Europe (2008-12): an analysis of EuroTravNet longitudinal, surveillance data, and evaluation of the effect of the pre-travel consultation	Lancet Infectious Diseases	15	1	55-64	Cross-sectional studies	2008-01-01	2012-12-31	Several	100	NA	NA	NA	NA	NA	NA	NA	NA	NA	NA	NA	NA	NA	NA	NA	NA	NA	NA	NA	NA
349	Schmalzle, S. A., Tabatabai, A. et al.	2019	Recreational 'mud fever': Leptospira interrogans induced diffuse alveolar hemorrhage and severe acute respiratory distress syndrome in a U.S. Navy seaman following 'mud-run' in Hawaii	IDCases	15	NA	NA	Case report	2014-03-01	2014-03-31	United States	100	1	NA	1	NA	NA	NA	NA	1	NA	1	1	NA	1	NA	NA	NA	NA	1	NA	1
350	Schmid, D., Holzmann, H. et al.	2008	Mumps outbreak in young adults following a festival in Austria, 2006	Eurosurveillance	13	7	11-12	Cross-sectional studies	2006-05-01	2006-08-31	Austria	75	NA	NA	NA	NA	NA	NA	NA	NA	NA	NA	76	NA	NA	NA	NA	76	NA	NA	76	214
351	Segel, M. J., Lindsley, M. D. et al.	2015	Histoplasmosis in Israeli Travelers	American Journal Of Tropical Medicine & Hygiene	92	6	1168-1172	Case series	2000-01-01	2012-12-31	Israel	85	10	1	6	3	NA	NA	4	6	NA	23	23	1	14	6	2	NA	6	17	NA	23
352	Sejvar, J., Bancroft, E. et al.	2003	Leptospirosis in "Eco-Challenge" Athletes, Malaysian Borneo, 2000	Emerging Infectious Diseases	9	6	702-707	Cross-sectional studies	2000-09-07	2000-09-30	Malaysia	62	80	NA	NA	NA	NA	80	NA	NA	80	189	26	NA	NA	NA	NA	26	NA	NA	26	189
353	Sekizuka, T., Kuramoto, S. et al.	2020	SARS-CoV-2 Genome Analysis of Japanese Travelers in Nile River Cruise	Frontiers In Microbiology	11	NA	NA	Case series	2020-03-05	2020-03-15	Japan	60	10	NA	NA	1	9	NA	6	4	NA	10	10	NA	NA	1	9	NA	6	4	NA	10
354	Shahkarami, M., Yen, C. et al.	2015	Laboratory Testing for Middle East Respiratory Syndrome Coronavirus, California, USA, 2013-2014	Emerging Infectious Diseases	21	9	1664-1666	Cross-sectional studies	2013-02-01	2014-11-30	United States	69	NA	NA	NA	NA	NA	NA	NA	NA	NA	NA	32	NA	NA	NA	NA	32	NA	NA	32	52
355	Sharafeldin, E., Soonawala, D. et al.	2010	Health risks encountered by Dutch medical students during an elective in the tropics and the quality and comprehensiveness of pre- and post-travel care	BMC Medical Education	10	1	89	Cross-sectional studies	2006-07-01	2008-12-31	Netherlands	81	NA	NA	NA	NA	NA	NA	NA	NA	NA	180	20	NA	NA	NA	NA	20	NA	NA	20	180
356	Shaw, M. T. M., Leggat, P. A.	2006	Life and death on the Amazon: Illness and injury to travelers on a South American expedition	Journal Of Travel Medicine	10	5	268-271	Cross-sectional studies	2001-01-01	2001-12-31	Several	50	NA	NA	NA	NA	NA	NA	NA	NA	NA	NA	29	NA	NA	NA	NA	29	NA	NA	29	78
357	Shaw, M. T. M., Leggat, P. A. et al.	2007	Illness in returned travellers presenting at GeoSentinel sites in New Zealand	Australian & New Zealand Journal Of Public Health	27	1	82-86	Cross-sectional studies	1997-11-01	2001-12-01	New Zealand	56	NA	NA	NA	NA	NA	NA	NA	NA	NA	NA	10	NA	NA	NA	NA	10	NA	NA	10	205
358	Sheng, W. H., Liu, W. D. et al.	2020	Dysosmia and dysgeusia in patients with COVID-19 in northern Taiwan	Journal Of Formosan Medical Association	120	1	311-317	Cohort studies	2020-01-22	2020-05-07	China	77	NA	NA	NA	NA	NA	NA	NA	NA	NA	NA	189	NA	NA	NA	NA	189	NA	NA	189	217
359	Shiel, E., Miyakis, S. et al.	2021	Clinical characteristics and outcomes of COVID-19 in a low-prevalence, well resourced setting, Sydney, Australia	Internal Medicine Journal	51	10	1605-1613	Cross-sectional studies	2020-01-01	2020-06-30	Australia	94	NA	NA	NA	NA	NA	NA	NA	NA	NA	NA	312	NA	NA	NA	NA	312	NA	NA	312	517

Study ID	Authors	Publication Year	Title	Journal	Volume	Issue	Pages	Study Design	Starting date	Ending date	Country of detection	JBI Critical Score (%)	Respiratory Symptoms: Total cases (n)	Respiratory Symptoms: Age Distribution: Child/Young	Adult	Middle Age Adult	Senior Adult	Unknown Age	Respiratory Symptoms: Gender Distribution: Female	Male	Unknown Gender	Respiratory Symptoms: Total population (N)	Respiratory Cases Reported: Total cases (n)	Respiratory Cases Reported: Age Distribution: Child/Young	Adult	Middle Age Adult	Senior Adult	Unknown Age	Respiratory Cases Reported: Gender Distribution: Female	Male	Unknown Gender	Respiratory Cases Reported: Total population (N)
360	Shorten, R. J., Norman, J. et al.	2019	An unusual presentation of Legionella pneumonia in a returning traveller	BMJ Case Reports	12	8	NA	Case report	NA	NA	UK	75	1	NA	NA	NA	1	NA	NA	1	NA	1	1	NA	NA	NA	1	NA	NA	1	NA	1
361	Skrip, L. A., Selvaraj, P. et al.	2021	Seeding COVID-19 across Sub-Saharan Africa: An Analysis of Reported Importation Events across 49 Countries	American Journal Of Tropical Medicine & Hygiene	104	5	1694-1702	Cross-sectional studies	2020-02-27	2020-04-21	Several	69	NA	NA	NA	NA	NA	NA	NA	NA	NA	NA	1129	NA	NA	NA	NA	1129	185	343	NA	NA
362	Sohail, A., Mcguinness, S. L. et al.	2018	Spectrum of illness among returned Australian travellers from Bali, Indonesia: a 5-year retrospective observational study	Internal Medicine Journal	49	1	34-40	Cross-sectional studies	2011-01-01	2015-12-31	Australia	81	NA	NA	NA	NA	NA	NA	NA	NA	NA	NA	51	NA	NA	NA	NA	51	NA	NA	51	464
363	Sokhna, C., Mboup, B. M. et al.	2017	Communicable and non-communicable disease risks at the Grand Magal of Touba: The largest mass gathering in Senegal	Travel Medicine & Infectious Disease	19	NA	56-60	Cross-sectional studies	2015-11-01	2015-11-30	Senegal	44	NA	NA	NA	NA	NA	NA	NA	NA	NA	NA	3255	NA	NA	NA	NA	3255	NA	NA	3255	32229
364	Speake, H., Phillips, A. et al.	2020	Flight-Associated Transmission of Severe Acute Respiratory Syndrome Coronavirus 2 Corroborated by Whole-Genome Sequencing	Emerging Infectious Diseases	26	12	2872-2880	Prevalence studies	2020-03-19	2020-04-01	Australia	100	29	NA	NA	NA	NA	29	NA	NA	29	241	29	NA	NA	NA	NA	29	NA	NA	29	241
365	Stienlauf, S., Segal, G. et al.	2006	Epidemiology of Travel-Related Hospitalization	Journal Of Travel Medicine	12	3	136-141	Cross-sectional studies	1999-01-01	2003-12-31	Israel	75	NA	NA	NA	NA	NA	NA	NA	NA	NA	NA	16	NA	NA	NA	NA	16	NA	NA	16	211
366	Stojanovic, K. S., Lionnet, F. et al.	2011	The risk of going abroad in sickle cell disease: a study of 148 adults	Transactions Of Royal Society Of Tropical Medicine & Hygiene	105	6	310-314	Prevalence studies	2007-06-01	2008-04-30	France	72	6	NA	NA	NA	NA	6	NA	NA	6	53	NA	NA	NA	NA	NA	NA	NA	NA	NA	NA
367	Stoney, R. J., Esposito, D. H. et al.	2018	Infectious diseases acquired by international travellers visiting the USA	Journal Of Travel Medicine	25	NA	NA	Cross-sectional studies	1997-01-01	2016-12-31	Several	88	NA	NA	NA	NA	NA	NA	NA	NA	NA	NA	226	NA	NA	NA	NA	226	NA	NA	226	1393
368	Sumadewi, K. T., Narendrakomaranatha, A. et al.	2018	Travel related infection among international travellers visiting clinics at Ubud Bali	3rd Annual Applied Science & Engineering Conference, Aasec 2018	434	NA	NA	Cross-sectional studies	2017-09-01	2017-10-31	Indonesia	69	NA	NA	NA	NA	NA	NA	NA	NA	NA	NA	62	16	NA	2	8	36	35	27	NA	453
369	Suryapranata, F., Boyd, A. et al.	2019	Symptoms of infectious diseases in HIV-positive travellers: A prospective study with exposure-matched controls	Travel Medicine & Infectious Disease	29	NA	28-33	Cohort studies	2003-09-01	2012-09-30	Netherlands	73	32	NA	NA	NA	NA	32	NA	NA	32	104	NA	NA	NA	NA	NA	NA	NA	NA	NA	NA
370	Svoboda, T., Henry, B. et al.	2004	Public health measures to control the spread of the severe acute respiratory syndrome during the outbreak in Toronto	New England Journal Of Medicine	350	23	2352-2361	Cross-sectional studies	2003-02-23	2003-07-01	Canada	94	NA	NA	NA	NA	NA	NA	NA	NA	NA	NA	4	NA	NA	NA	NA	4	1	NA	3	225
371	Taha, M. K., Achtman, M. et al.	2000	Serogroup W135 meningococcal disease in Hajj pilgrims	Lancet	356	9248	2159	Case series	2000-03-20	2000-05-10	Several	55	NA	NA	NA	NA	NA	NA	NA	NA	NA	NA	15	NA	NA	NA	NA	15	NA	NA	15	52
372	Taha, M. K., Kacou-N'douba, A. et al.	2013	Travel-related Neisseria meningitidis Serogroup W135 Infection, France	Emerging Infectious Diseases	19	6	1030-1032	Case series	2012-01-01	2012-03-11	France	60	NA	NA	NA	NA	NA	NA	NA	NA	NA	NA	14	9	NA	NA	3	2	8	6	NA	14
373	Te, A. J., Binnicker, M. J. et al.	2021	SARS-CoV-2 Testing Before International Airline Travel, December 2020 to May 2021	Mayo Clinic Proceedings	96	11	2856-2860	Cross-sectional studies	2020-12-19	2021-05-19	Several	62	NA	NA	NA	NA	NA	NA	NA	NA	NA	NA	5	NA	NA	NA	NA	5	NA	NA	5	9849
374	Team, C. N. I. R. S.	2020	D-19, Australia: Epidemiology Report 20 (Fortnightly reporting period ending 5 July 2020)	Communicable Diseases Intelligence	44	NA	NA	Cross-sectional studies	2020-01-01	2020-09-05	Australia	88	NA	NA	NA	NA	NA	NA	NA	NA	NA	NA	4963	NA	NA	NA	NA	4963	NA	NA	4963	8570
375	Team, H. R., Barasheed, O. et al.	2014	Viral respiratory infections among Hajj pilgrims in 2013	Virologica Sinica	29	6	364-371	Cross-sectional studies	2013-09-12	2013-09-17	Saudi Arabia	62	112	NA	NA	NA	NA	112	63	49	NA	1038	42	NA	NA	NA	NA	42	NA	NA	42	1038
376	Theocharis, G., Polyzos, K. A. et al.	2012	Morbidity of foreign travelers in Attica, Greece: a retrospective study	European Journal Of Clinical Microbiology & Infectious Diseases	31	9	2141-2146	Cross-sectional studies	2005-01-01	2009-12-31	Greece	88	NA	NA	NA	NA	NA	NA	NA	NA	NA	NA	1165	NA	NA	NA	NA	1165	NA	NA	1165	3414
377	Thi Loi, D., Van Thuan, H. et al.	2019	Infectious disease symptoms and microbial carriage among French medical students travelling abroad: A prospective study	Travel Medicine & Infectious Disease	34	NA	NA	Cross-sectional studies	2018-06-01	2018-08-31	France	94	52	NA	NA	NA	NA	52	NA	NA	52	134	29	NA	NA	NA	NA	29	NA	NA	29	120
378	Thomas, H. L., Zhao, H. et al.	2014	Enhanced MERS Coronavirus Surveillance of Travelers from the Middle East to England	Emerging Infectious Diseases	20	9	1562-1564	Cross-sectional studies	2012-09-24	2013-10-15	UK	75	NA	NA	NA	NA	NA	NA	NA	NA	NA	NA	24	NA	NA	NA	NA	24	NA	NA	24	77
379	Thompson, D. T., Ashley, D. V. M. et al.	2006	Incidence of Health Crises in Tourists Visiting Jamaica, West Indies, 1998 to 2000	Journal Of Travel Medicine	10	2	79-86	Cross-sectional studies	1998-06-01	2002-06-30	UK	62	NA	NA	NA	NA	NA	NA	NA	NA	NA	NA	29	NA	NA	NA	NA	29	17	12	NA	801
380	To, K. K. W., Ng, K. H. L. et al.	2012	Avian influenza A H5N1 virus: a continuous threat to humans	Emerging Microbes & Infections	1	NA	NA	Case report	2020-11-01	2020-11-30	China	75	1	NA	NA	1	NA	NA	1	NA	NA	1	1	NA	NA	1	NA	NA	1	NA	NA	1
381	Tochitani, K., Iwamoto, N. et al.	2020	Imported and locally transmitted mild SARS-CoV-2 pneumonia cases in Japan	Journal Of Infection & Chemotherapy	26	8	854-857	Case report	2020-01-28	2020-02-11	Japan	88	1	NA	1	NA	NA	NA	1	NA	NA	1	1	NA	1	NA	NA	NA	1	NA	NA	1

Study ID	Authors	Publication Year	Title	Journal	Volume	Issue	Pages	Study Design	Starting date	Ending date	Country of detection	JBI Critical Score (%)	Respiratory Symptoms										Respiratory Cases Reported									
													Total cases (n)	Age Distribution					Gender Distribution			Total population (N)	Total cases (n)	Age Distribution					Gender Distribution			Total population (N)
														Child/Young	Adult	Middle Age Adult	Senior Adult	Unknown Age	Female	Male	Unknown Gender			Child/Young	Adult	Middle Age Adult	Senior Adult	Unknown Age	Female	Male	Unknown Gender	
382	Toda, M., Gonzalez, F. J. et al.	2019	Notes from the Field: Multistate Coccidioidomycosis Outbreak in U.S. Residents Returning from Community Service Trips to Baja California, Mexico - July-August 2018	Morbidity & Mortality Weekly Report	68	14	332-333	Case series	2018-06-30	2018-08-11	United States	50	NA	NA	NA	NA	NA	NA	NA	NA	NA	NA	8	NA	NA	NA	NA	8	NA	8	NA	225
383	Toyokawa, T., Shimada, T. et al.	2021	Transmission of SARS-CoV-2 during a 2-h domestic flight to Okinawa, Japan, March 2020	Influenza & Other Respiratory Viruses	16	1	63-71	Cross-sectional studies	2020-03-23	2020-04-06	Japan	75	15	4	7	4	NA	NA	5	10	NA	146	15	4	8	4	NA	NA	5	10	NA	146
384	Valerio, L., Arranz, Y. et al.	2011	Caracterización epidemiológica y factores de riesgo asociados a la peregrinación religiosa a Arabia Saudí. Resultados de una cohorte prospectiva 2008-2009	Gaceta Sanitaria	26	3	251-255	Cross-sectional studies	2008-01-01	2009-12-31	Spain	100	16	NA	NA	NA	NA	16	NA	NA	16	193	NA	NA	NA	NA	NA	NA	NA	NA	NA	NA
385	Van Cuong, L., Giang, H. T. N. et al.	2020	The first Vietnamese case of COVID-19 acquired from China	Lancet Infectious Diseases	20	4	408-409	Case report	2020-01-17	2020-02-06	Vietnam	69	3	NA	1	NA	NA	2	1	NA	2	3	3	NA	1	NA	NA	2	1	NA	2	3
386	Van De Werve, C., Perignon, A. et al.	2013	TravelRelated Leptospirosis: A Series of 15 Imported Cases	Journal Of Travel Medicine	20	4	228-231	Case series	2008-01-01	2011-09-30	France	75	NA	NA	NA	NA	NA	NA	NA	NA	NA	NA	15	NA	NA	NA	NA	NA	1	14	NA	15
387	Van Hal, S. J., Foo, H. et al.	2009	Influenza Outbreak during Sydney World Youth Day 2008: The Utility of Laboratory Testing and Case Definitions on Mass Gathering Outbreak Containment	PLOS One	4	9	e6620	Cross-sectional studies	2008-07-15	2008-07-20	Australia	94	NA	NA	NA	NA	NA	NA	NA	NA	NA	242	45	NA	NA	NA	NA	45	NA	NA	45	242
388	Varkey, P., Jerath, A. U. et al.	2007	The Epidemiology of Tuberculosis Among Primary Refugee Arrivals in Minnesota Between 1997 and 2001	Journal Of Travel Medicine	14	1	1-8	Cross-sectional studies	1997-01-01	2001-12-31	united States	100	NA	NA	NA	NA	NA	NA	NA	NA	NA	NA	4990	1705	NA	NA	185	3100	1944	NA	NA	9842
389	Venkataram, T., Goyal, N. et al.	2020	Deployment of Neurosurgeons at the Warfront Against Coronavirus Disease of 2019 (COVID-19)	World Neurosurgery	144	NA	E561-E567	Cross-sectional studies	2020-04-25	2020-05-31	India	88	NA	NA	NA	NA	NA	NA	NA	NA	NA	NA	1	NA	NA	NA	NA	1	NA	NA	1	29
390	Venugopal, U., Jilani, N. et al.	2021	SARS-CoV-2 seroprevalence among health care workers in a New York City hospital: A cross-sectional analysis during the COVID-19 pandemic	International Journal Of Infectious Diseases	102	NA	63-69	Cross-sectional studies	2020-03-01	2020-05-01	United States	100	NA	NA	NA	NA	NA	NA	NA	NA	NA	NA	5	NA	NA	NA	NA	5	NA	NA	5	19
391	Verhoeven, P. O., Gautret, P. et al.	2015	Molecular dynamics of Staphylococcus aureus nasal carriage in Hajj pilgrims	Clinical Microbiology & Infection	21	7	650.e5-8	Cross-sectional studies	2012-10-23	2012-10-28	France	62	NA	NA	NA	NA	NA	NA	NA	NA	NA	NA	25	NA	NA	NA	NA	25	NA	NA	25	158
392	Vial, M. R., Peters, A. et al.	2020	Covid-19 in South America: clinical and epidemiological characteristics among 381 patients during the early phase of the pandemic in Santiago, Chile	BMC Infectious Diseases	20	1	NA	Cross-sectional studies	2020-03-03	2020-04-04	Chile	88	NA	NA	NA	NA	NA	NA	NA	NA	NA	NA	46	NA	NA	NA	NA	46	NA	NA	46	381
393	Viasus, D., Di Yacovo, S. et al.	2013	Community-Acquired Legionella pneumophila Pneumonia A Single-Center Experience With 214 Hospitalized Sporadic Cases Over 15 Years	Medicine	92	1	51-60	Cross-sectional studies	1995-02-13	2010-12-31	Spain	88	NA	NA	NA	NA	NA	NA	NA	NA	NA	NA	34	NA	NA	NA	NA	34	NA	NA	34	1560
394	Vilkman, K., Pakkanen, S. H. et al.	2016	Travelers' health problems and behavior: Prospective study with post-travel follow-up	BMC Infectious Diseases	16	1	NA	Cross-sectional studies	2008-12-01	2010-02-01	Finland	88	26	NA	NA	NA	NA	26	NA	NA	26	459	12	NA	NA	NA	NA	12	NA	NA	12	459
395	Vlot, J. A., Blanter, A. I. et al.	2020	Travel preparation and health risks in Dutch and Belgian medical students during an elective in low- or middle-income countries: A prospective self-reporting cohort study	Travel Medicine & Infectious Disease	37	NA	101779	Cross-sectional studies	2010-07-01	2016-11-30	Several	88	NA	NA	NA	NA	NA	NA	NA	NA	NA	NA	11	NA	NA	NA	NA	11	NA	NA	11	464
396	Vlot, J. A., Vive, M. G. D. et al.	2020	Predicting morbidity in older travellers during a short-term stay in the tropics: the ELDEST study	Journal Of Travel Medicine	28	1	NA	Cross-sectional studies	2016-07-01	2017-11-30	Netherlands	100	98	NA	NA	NA	NA	98	NA	NA	98	475	NA	NA	NA	NA	NA	NA	NA	NA	NA	NA
397	Vogt, T. M., Guerra, M. A. et al.	2006	Risk of Severe Acute Respiratory SyndromeAssociated Coronavirus Transmission Aboard Commercial Aircraft	Journal Of Travel Medicine	13	5	268-272	Cross-sectional studies	2003-02-01	2003-03-31	United States	62	17	NA	NA	NA	NA	17	NA	NA	17	312	0	NA	NA	NA	NA	0	NA	NA	0	312
398	Walker, L. J., Codreanu, T. A. et al.	2021	SARS-CoV-2 infections among Australian passengers on the Diamond Princess cruise ship: A retrospective cohort study	PLOS One	16	9	NA	Cross-sectional studies	2020-03-01	2020-04-06	Several	100	97	NA	NA	NA	NA	97	NA	NA	97	196	56	3	5	14	34	NA	30	26	NA	223

Study ID	Authors	Publication Year	Title	Journal	Volume	Issue	Pages	Study Design	Starting date	Ending date	Country of detection	JBI Critical Score (%)	Respiratory Symptoms										Respiratory Cases Reported									
													Total cases (n)	Age Distribution					Gender Distribution			Total population (N)	Total cases (n)	Age Distribution					Gender Distribution			Total population (N)
														Child/Young	Adult	Middle Age Adult	Senior Adult	Unknown Age	Female	Male	Unknown Gender			Child/Young	Adult	Middle Age Adult	Senior Adult	Unknown Age	Female	Male	Unknown Gender	
399	Wang, L., Ma, H. et al.	2020	Heterogeneity in testing, diagnosis and outcome in SARS-CoV-2 infection across outbreak settings in the Greater Toronto Area, Canada: an observational study	CMAJ Open	8	4	E627-e636	Cross-sectional studies	2020-01-23	2020-05-20	Canada	62	NA	NA	NA	NA	NA	NA	NA	NA	NA	NA	674	NA	NA	NA	NA	674	NA	NA	674	16490
400	Ward, K. A., Armstrong, P. et al.	2010	Outbreaks of Pandemic (H1N1) 2009 and Seasonal Influenza A (H3N2) on Cruise Ship	Emerging Infectious Diseases	16	11	1731-1737	Cross-sectional studies	NA	2009-05-25	Australia	88	NA	NA	NA	NA	NA	NA	NA	NA	NA	100	178	51	37	NA	12	78	96	82	NA	1970
401	Warne, B., Weld, L. H. et al.	2014	Travel-related infection in european travelers, EuroTravNet 2011	Journal Of Travel Medicine	21	4	248-254	Cross-sectional studies	2011-01-01	2011-12-31	Several	88	51	NA	NA	NA	NA	51	NA	NA	51	5965	303	NA	NA	NA	NA	303	NA	NA	303	5965
402	Wedege, E., Bolstad, K. et al.	2012	Molecular characterization of clinical and environmental isolates of Legionella pneumophila in Norway, 2001-2008	Scandinavian Journal Of Infectious Diseases	45	1	59-64	Cross-sectional studies	2001-01-01	2008-12-31	Norway	62	NA	NA	NA	NA	NA	NA	NA	NA	NA	NA	11	NA	NA	NA	NA	11	6	5	NA	45
403	Whelan, J., Rimmelzwaan, G. F. et al.	2016	Influenza in long-term Dutch travelers in the tropics: symptoms and infections	BMC Infectious Diseases	16	1	158	Cross-sectional studies	2008-12-01	2011-09-30	Netherlands	100	NA	NA	NA	NA	NA	NA	NA	NA	NA	602	90	NA	39	NA	NA	51	63	27	NA	602
404	Wieten, R. W., Leenstra, T. et al.	2012	Health Risks of Travelers With Medical ConditionsuA Retrospective Analysis	Journal Of Travel Medicine	19	2	104-110	Cross-sectional studies	2010-01-01	2010-10-31	Netherlands	88	NA	NA	NA	NA	NA	NA	NA	NA	NA	NA	9	NA	NA	NA	4	5	NA	NA	9	420
405	Wilder-Smith, A., Earnest, A. et al.	2003	High Incidence of Pertussis among Hajj Pilgrims	Clinical Infectious Diseases	37	9	1270-1272	Cross-sectional studies	NA	NA	Singapour	62	204	NA	NA	NA	NA	204	NA	NA	204	358	5	NA	NA	NA	NA	5	NA	NA	5	358
406	Wilder-Smith, A., Foo, W. et al.	2005	High risk of Mycobacterium tuberculosis infection during the Hajj pilgrimag	Tropical Medicine & International Health	10	4	336-339	Cross-sectional studies	2002-02-20	2002-02-25	Singapour	62	202	NA	NA	NA	NA	202	NA	NA	202	365	15	NA	NA	NA	NA	15	NA	NA	15	357
407	Wilder-Smith, A., M S Barkham,T. et al.	2002	Acquisition of W135 meningococcal carriage in Hajj pilgrims and transmission to household contacts: prospective study	BMJ	325	7360	365-366	Cross-sectional studies	2001-03-03	2001-03-08	Singapour	56	NA	NA	NA	NA	NA	NA	NA	NA	NA	NA	29	NA	NA	NA	NA	29	NA	NA	29	171
408	Wilder-Smith, A., Paton, N. I. et al.	2003	Short communication: Low risk of transmission of severe acute respiratory syndrome on airplanes: the Singapore experience: Low risk of SARS transmission on airplanes	Tropical Medicine & International Health	8	11	1035-1037	Case series	2003-02-25	2003-05-31	Singapour	65	4	NA	NA	NA	NA	4	NA	NA	4	9	9	NA	NA	NA	NA	9	NA	NA	9	9
409	Wildersmith, A., Goh, K. T. et al.	2003	Hajj-Associated Outbreak Strain of Neisseria meningitidis Serogroup W135: Estimates of the Attack Rate in a Defined Population and the Risk of Invasive Disease Developing in Carriers	Clinical Infectious Diseases	36	6	679-683	Case series	2000-01-01	2001-12-31	Singapour	55	NA	NA	NA	NA	NA	NA	NA	NA	NA	NA	8	3	2	2	1	NA	6	2	NA	12
410	William, T., Thevarajah, B. et al.	2015	Avian Influenza (H7N9) Virus Infection in Chinese Tourist in Malaysia, 2014	Emerging Infectious Diseases	21	1	142-145	Case report	2014-01-30	2014-03-16	Malaysia	88	1	NA	NA	NA	1	NA	1	NA	NA	1	1	NA	NA	NA	1	NA	1	NA	NA	1
411	Williams, G. H., Llewelyn, A. et al.	2021	SARS-CoV-2 testing and sequencing for international arrivals reveals significant cross border transmission of high risk variants into the United Kingdom	Eclinicalmedicine	38	NA	NA	Cross-sectional studies	2021-03-11	2021-04-14	UK	75	NA	NA	NA	NA	NA	NA	NA	NA	NA	NA	3855	NA	NA	NA	NA	3855	NA	NA	3855	203065
412	Wilson, M. E., Chen, L. H. et al.	2014	Illness in travelers returned from Brazil: The geosentinel experience and implications for the 2014 FIFA world cup and the 2016 summer olympics	Clinical Infectious Diseases	58	10	1347-1356	Cross-sectional studies	1997-09-01	2013-05-31	Several	88	NA	NA	NA	NA	NA	NA	NA	NA	NA	NA	41	NA	NA	NA	NA	41	NA	NA	41	1586
413	Wilson, M. E., Weld, L. H. et al.	2007	Fever in Returned Travelers: Results from the GeoSentinel Surveillance Network	Clinical Infectious Diseases	44	12	1560-1568	Cross-sectional studies	1997-03-01	2006-03-31	Several	100	NA	NA	NA	NA	NA	NA	NA	NA	NA	NA	978	NA	NA	NA	NA	978	NA	NA	978	6957
414	Wong, A. H., Cheng, P. K. C. et al.	2012	Virulence Potential of Fusogenic Orthoreoviruses	Emerging Infectious Diseases	18	6	944-948	Case series	2007-01-01	2010-12-31	China	60	3	NA	2	1	NA	NA	2	1	NA	3	3	NA	2	1	NA	NA	2	1	NA	3
415	Wood, S., Telu, K. et al.	2018	Influenza-like illness in travelers to the developing world	American Journal Of Tropical Medicine & Hygiene	99	5	1269-1274	Cross-sectional studies	2010-01-01	2016-03-31	Several	94	NA	NA	NA	NA	NA	NA	NA	NA	NA	2932	NA	NA	NA	NA	NA	NA	NA	NA	NA	NA
416	Wu, C. H., Chou, Y. C. et al.	2021	Epidemiological features of domestic and imported cases with COVID-19 between January 2020 and March 2021 in Taiwan	Medicine	100	39	NA	Cross-sectional studies	2020-01-01	2021-03-31	China	94	NA	NA	NA	NA	NA	NA	NA	NA	NA	NA	953	45	635	189	84	NA	464	489	NA	1030
417	Wu, Y., Liu, M. Y. et al.	2020	Epidemiology of imported infectious diseases, China, 2014-18	Journal Of Travel Medicine	27	8	NA	Cross-sectional studies	2014-01-01	2018-12-31	China	81	NA	NA	NA	NA	NA	NA	NA	NA	NA	NA	31460	NA	NA	NA	NA	31460	NA	NA	31460	58851
418	Xiao, S., Liu, Y. et al.	2021	Epidemiological characteristics of COVID-19 clusters in Hainan, China	Medicine	100	42	NA	Cross-sectional studies	2020-01-22	2020-02-19	China	100	NA	NA	NA	NA	NA	NA	NA	NA	NA	NA	7	NA	NA	NA	NA	7	NA	NA	7	99
419	Yaita, K., Sakai, Y. et al.	2015	Post-travel consultations in a Regional Hub city hospital, Japan	Internal Medicine Journal	55	7	739-743	Cross-sectional studies	2008-04-01	2014-10-31	Japan	88	NA	NA	NA	NA	NA	NA	NA	NA	NA	NA	9	NA	NA	NA	NA	9	NA	NA	9	55

Study ID	Authors	Publication Year	Title	Journal	Volume	Issue	Pages	Study Design	Starting date	Ending date	Country of detection	JBI Critical Score (%)	Respiratory Symptoms										Respiratory Cases Reported									
													Total cases (n)	Age Distribution					Gender Distribution			Total population (N)	Total cases (n)	Age Distribution					Gender Distribution			Total population (N)
														Child/Young	Adult	Middle Age Adult	Senior Adult	Unknown Age	Female	Male	Unknown Gender			Child/Young	Adult	Middle Age Adult	Senior Adult	Unknown Age	Female	Male	Unknown Gender	
420	Yavarian, J., Shafiei Jandaghi, N. Z. et al.	2017	Influenza virus but not MERS coronavirus circulation in Iran, 20132016: Comparison between pilgrims and general population	Travel Medicine & Infectious Disease	21	NA	51-55	Cross-sectional studies	2013-01-01	2016-12-31	Iran (Islamic Republic of)	69	NA	NA	NA	NA	NA	NA	NA	NA	NA	NA	499	NA	NA	NA	NA	499	NA	NA	499	3840
421	Yoshimura, Y., Sasaki, H. et al.	2020	Clinical characteristics of the coronavirus disease 2019 (COVID-19) outbreak on a cruise ship	Journal Of Infection & Chemotherapy	26	11	1177-1180	Cross-sectional studies	2020-02-05	2020-02-19	Japan	88	17	NA	NA	NA	NA	17	9	8	NA	17	17	NA	NA	NA	NA	17	9	8	NA	17
422	Young, N., Pebody, R. et al.	2013	International flight-related transmission of pandemic influenza A(H1N1)pdm09: an historical cohort study of the first identified cases in the United Kingdom	Influenza & Other Respiratory Viruses	8	1	66-73	Cross-sectional studies	2009-06-09	2009-08-05	UK	50	NA	NA	NA	NA	NA	NA	NA	NA	NA	232	10	NA	NA	NA	NA	10	NA	NA	10	232
423	Yue, Y., Chen, Y. et al.	2021	A survey of a COVID-19 cluster of charter flight importation	Public Health	199	NA	107-109	Cross-sectional studies	2020-10-01	2020-10-31	China	56	13	NA	NA	NA	NA	13	NA	NA	13	159	36	NA	NA	NA	NA	36	NA	NA	36	159
424	Zanotti, C., Munari, S. et al.	2018	Burkholderia gladioli sinonasal infection	European Annals Of Otorhinolaryngology, Head & Neck Diseases	136	1	55-56	Case report	2015-11-01	2017-05-01	Italy	81	NA	NA	NA	NA	NA	NA	NA	NA	NA	NA	1	NA	NA	1	NA	NA	1	NA	NA	1
425	Zhang, J., Qin, F. et al.	2021	Transmission of SARS-CoV-2 during air travel: a descriptive and modelling study	Annals Of Medicine	53	1	1569-1575	Cross-sectional studies	2021-03-01	2021-03-31	China	69	NA	NA	NA	NA	NA	NA	NA	NA	NA	NA	161	NA	NA	NA	NA	161	99	62	NA	4492
426	Zhang, Z. B., Li, L. et al.	2020	Countries of origin of imported COVID-19 cases into China and measures to prevent onward transmission	Journal Of Travel Medicine	27	8	NA	Cross-sectional studies	2020-03-11	2020-07-06	China	88	183	NA	NA	NA	NA	183	NA	NA	183	268	268	NA	NA	NA	NA	268	110	158	NA	268
427	Zhao, N., Liu, Y. et al.	2020	Tracking the origin of early COVID-19 cases in Canada	International Journal Of Infectious Diseases	96	NA	506-508	Cross-sectional studies	2020-01-27	2020-03-11	Canada	62	NA	NA	NA	NA	NA	NA	NA	NA	NA	NA	99	NA	NA	NA	NA	99	NA	NA	99	118
428	Ziyaeyan, M., Alborzi, A. et al.	2012	Pandemic 2009 influenza A (H1N1) infection among 2009 Hajj Pilgrims from Southern Iran: a real-time RT-PCR-based study: A(H1N1)pdm09 infection among 2009 Hajj Pilgrims from Iran	Influenza & Other Respiratory Viruses	6	6	e80-e84	Cross-sectional studies	2009-12-08	2009-12-11	Iran (Islamic Republic of)	75	NA	NA	NA	NA	NA	NA	NA	NA	NA	305	13	NA	1	11	1	NA	9	4	NA	305
429	Zvereva, N. N., Saifullin, M. A. et al.	2019	Epidemiological and etiological features of travel-related febrile illnesses in hospitalized Russian children and adults: A single-centre, retrospective analysis in Moscow	Travel Medicine & Infectious Disease	34	NA	NA	Cross-sectional studies	2009-01-01	2017-12-31	Russia	75	NA	NA	NA	NA	NA	NA	NA	NA	NA	NA	788	NA	NA	NA	NA	788	NA	NA	788	1719

Appendix (section 3). Absolute number of specific respiratory symptom or symptoms associated with a respiratory symptom by UN region or specific acquisition area.

Symptoms	Total cases	United Nations region name					Specific cases			
		Africa	Americas	Asia	Europe	Oceania	Airplane	Cruise or Merchant Vessel	Refugee and Asylum-Seeker	Mass gatherings events[a]
**Respiratory symptoms**	86,841	250	1,166	687	59	1	93	465	341	11,033
Cough	11,206	34	260	129	32	N/A	44	962	3	8,556
Dyspnea	1,616	9	152	15	2	N/A	4	59	N/A	721
Expectoration	1,355	N/A	N/A	N/A	N/A	N/A	N/A	590	N/A	765
Loss of sense of smell or taste	27	N/A	26	N/A	N/A	1	N/A	N/A	N/A	N/A
Rinithis/Runny nose/Congestion	4,953	N/A	N/A	5	10	1	11	185	N/A	3,591
Sinus pain	58	N/A	N/A	N/A	N/A	N/A	N/A	N/A	N/A	58
Sore throat	6,511	2	45	13	14	N/A	23	797	N/A	4,950
Voice failure/Hoarseness	790	N/A	N/A	N/A	1	N/A	N/A	N/A	N/A	789
Weezing	180	N/A	N/A	N/A	N/A	N/A	N/A	N/A	N/A	180
**Additional symptoms associated with at least one respiratory symptom**										
**General**										
Asthenia	163	2	21	5	N/A	N/A	N/A	79	N/A	16
Fever	4,309	32	367	151	28	N/A	31	599	1	2,518
Headache	1,519	2	264	7	6	N/A	11	684	N/A	433
Thoracic pain	159	N/A	151	1	N/A	N/A	N/A	N/A	1	N/A
**Musculoskeletal**										
Arthralgia	177	N/A	23	4	7	N/A	N/A	12	N/A	110
Myalgia	1,859	2	232	5	2	N/A	3	536	N/A	786
**Gastrointestinal symptoms**										
Abdominal pain	5	N/A	5	N/A	N/A	N/A	N/A	N/A	N/A	N/A
Chills	13	1	12	N/A	N/A	N/A	N/A	N/A	N/A	N/A
Diarrhea	102	1	96	N/A	3	N/A	1	N/A	N/A	1
Nausea	1	1	N/A	N/A	N/A	N/A	N/A	N/A	N/A	N/A
Vomitting	5	N/A	3	N/A	N/A	N/A	N/A	N/A	N/A	2
**Syndromes**										
Common cold-like syndrome (ccls)	108,841	N/A	N/A	N/A	N/A	N/A	N/A	N/A	N/A	108,841
Infectious mononucleosis-like syndrome	124	N/A	N/A	N/A	N/A	N/A	N/A	N/A	N/A	N/A
Influenza-like illness	50,317	75	292	185	35	N/A	67	413	6	45,222
Sars-like illness	4	N/A	N/A	N/A	N/A	N/A	4	N/A	N/A	N/A
**Miscellaneous**										
Other	25	4	21	N/A	N/A	N/A	N/A	N/A	N/A	N/A

Includes cases of:[a]AsiaWorld-Expo, Bābā Farīd, Easter Festival (Carinthia), Eco-Challenge, EXIT Festival, Grand Magal of Touba, Hajj, Rock Werchter, Sojourn, Sziget Festival, Tablighi Jamaat,Umrah, Universiade, Winter Olympic & Paralympic Games, World Youth Day

Appendix (section 4). Absolute number of medically diagnosed respiratory cases by UN region or specific acquisition area.

Medical Diagnostic	Total cases	United Nations region name					Specific cases			
		Africa	Americas	Asia	Europe	Oceania	Airplane	Cruise or Merchant Vessel	Refugee and Asylum-Seeker	Mass gatherings events[a]
**Respiratory tract infection (RTI)**	126,710	1,599	1,786	7,778	7,432	42	1	352	10,798	69,642
**Upper respiratory tract infection (URTI)**	61,553	418	568	4,491	83	37	N/A	292	59	40,826
**Inflammation**	42,948	1	80	1,485	N/A	N/A	N/A	228	29	39,860
Epiglottitis	1	N/A	N/A	N/A	N/A	N/A	N/A	N/A	N/A	N/A
Laryngitis	25	N/A	N/A	N/A	N/A	N/A	N/A	N/A	N/A	N/A
Nasopharyngitis	5	N/A	N/A	N/A	N/A	N/A	N/A	N/A	N/A	N/A
Otitis	615	N/A	N/A	420	N/A	N/A	N/A	N/A	20	14
Otitis externa	5	N/A	N/A	N/A	N/A	N/A	N/A	N/A	N/A	5
Otitis media	592	N/A	N/A	420	N/A	N/A	N/A	N/A	2	9
Pharyngitis	31,396	N/A	N/A	554	N/A	N/A	N/A	N/A	3	30,400
Rhintis	147	N/A	N/A	122	N/A	N/A	N/A	N/A	3	N/A
Sinusitis	10,056	1	80	389	N/A	N/A	N/A	N/A	3	9,273
Tonsilitis	700	N/A	N/A	N/A	N/A	N/A	N/A	228	N/A	173
Tonsillitis pharyngitis	3	N/A	N/A	N/A	N/A	N/A	N/A	N/A	N/A	N/A
**Vaccine-Preventable Diseases**	718	35	20	118	81	37	N/A	N/A	16	94
Diphteria	53	4	N/A	8	N/A	34	N/A	N/A	N/A	N/A
Measles	211	16	N/A	45	30	N/A	N/A	N/A	9	13
Mumps	169	3	2	19	15	N/A	N/A	N/A	6	76
Pertussis	227	12	18	43	29	3	N/A	N/A	1	5
Rubella	58	N/A	N/A	3	7	N/A	N/A	N/A	N/A	N/A
**Lower respiratory tract infections (LRTI)**	37,104	1,131	917	3,116	7,345	5	1	60	10,597	2,236
Bronchitis	2,975	1	165	1,573	N/A	1	N/A	4	2	457
Pleurisy	14	N/A	N/A	N/A	N/A	N/A	N/A	N/A	N/A	N/A
Pneumonia	16,063	65	436	882	6,324	4	1	56	53	1,760
Atypical pneumonia	12,243	43	346	319	6,292	3	1	56	43	51
Blastomycosis	52	N/A	50	2	N/A	N/A	N/A	N/A	N/A	N/A
Brucellosis	45	N/A	N/A	1	N/A	N/A	N/A	N/A	N/A	N/A
Coccidioidomycosis	34	N/A	33	N/A	N/A	N/A	N/A	N/A	1	N/A
Q Fever	41	2	1	N/A	N/A	N/A	N/A	N/A	N/A	1
Histoplasmosis	261	17	180	1	N/A	N/A	N/A	N/A	31	N/A
Legionellosis	11,282	1	31	121	6,292	N/A	1	56	N/A	24
Leptospirosis	384	17	37	111	N/A	2	N/A	N/A	N/A	26

Medical Diagnostic	Total cases	United Nations region name					Specific cases			
		Africa	Americas	Asia	Europe	Oceania	Airplane	Cruise or Merchant Vessel	Refugee and Asylum-Seeker	Mass gatherings events[a]
Melioidosis	133	6	13	83	N/A	1	N/A	N/A	5	N/A
Paracoccidioidomycosis	8	N/A	1	N/A	N/A	N/A	N/A	N/A	6	N/A
Pneumocystis pneumonia (PCP)	2	N/A	N/A	N/A	N/A	N/A	N/A	N/A	N/A	N/A
Cystic pulmonary hydatidosis	1	N/A	N/A	N/A	N/A	N/A	N/A	N/A	N/A	N/A
Eosinophilic Pneumonia	8	N/A	N/A	N/A	N/A	N/A	N/A	N/A	2	N/A
Interstitial pneumonia	4	N/A	N/A	N/A	N/A	N/A	N/A	N/A	N/A	N/A
Tuberculosis	15,136	1,034	312	634	1,021	N/A	N/A	N/A	10,542	19
**Upper and lower respiratory tract infection**	26,632	N/A	N/A	23	N/A	N/A	N/A	N/A	N/A	26,579
Pharyngotracheitis	11	N/A	N/A	N/A	N/A	N/A	N/A	N/A	N/A	N/A
Sinobronchitis	26,579	N/A	N/A	N/A	N/A	N/A	N/A	N/A	N/A	26,579
Tracheobronchitis	42	N/A	N/A	23	N/A	N/A	N/A	N/A	N/A	N/A
**Miscellaneous**	1,227	50	301	148	4	N/A	N/A	N/A	142	1
Meningitis/Encephalitis	295	5	N/A	N/A	N/A	N/A	N/A	N/A	N/A	N/A
Varicella	377	N/A	N/A	18	4	N/A	N/A	N/A	142	1
Mononucleosis	555	45	301	130	N/A	N/A	N/A	N/A	N/A	N/A

Includes cases of:[a]AsiaWorld-Expo, Bābā Farīd, Easter Festival (Carinthia), Eco-Challenge, EXIT Festival, Grand Magal of Touba, Hajj, Rock Werchter, Sojourn, Sziget Festival, Tablighi Jamaat,Umrah, Universiade, Winter Olympic & Paralympic Games, World Youth Day

Appendix (section 5). Absolute number of molecularly diagnosed respiratory cases by UN region or specific acquisition area.

Molecular diagnostic	Total cases (n)	Area of acquisition (continent)					Specific circumstances of acquisition			
		Africa	Americas	Asia	Europe	Oceania	Airplane	Cruise or Merchant Vessel	Refugee and Asylum-Seeker	Mass gatherings events[a]
**VIRUS**	61,668	458	1,706	7,115	2,766	493	448	1,856	N/A	4,542
**Class I: Double stranded DNA (dsDNA) viruses**	565	6	29	24	N/A	N/A	6	N/A	N/A	91
**Adenoviridae**	225	1	2	11	N/A	N/A	6	N/A	N/A	78
**Herpesviridae**	338	5	27	11	N/A	N/A	N/A	N/A	N/A	13
HHV-3: Varicella-zoster virus (VZV)	7	N/A	N/A	N/A	N/A	N/A	N/A	N/A	N/A	N/A
HHV-4: Epstein–Barr virus (EBV)	201	1	18	8	N/A	N/A	N/A	N/A	N/A	N/A
HHV-5: Cytomegalovirus (CMV)	74	4	9	2	N/A	N/A	N/A	N/A	N/A	N/A
HHV-6: Human Herpesvirus 6	1	N/A	N/A	1	N/A	N/A	N/A	N/A	N/A	N/A
**Mimiviridae**	2	N/A	N/A	2	N/A	N/A	N/A	N/A	N/A	N/A
Acanthamoeba polyphaga mimivirus (APMV)	2	N/A	N/A	2	N/A	N/A	N/A	N/A	N/A	N/A
**Class II: Single stranded DNA (ssDNA) viruses**	3	N/A	N/A	1	N/A	N/A	N/A	N/A	N/A	2
**Parvoviridae**	3	N/A	N/A	1	N/A	N/A	N/A	N/A	N/A	2
Human bocavirus (HBoV)	3	N/A	N/A	1	N/A	N/A	N/A	N/A	N/A	2
**Class III: Double stranded RNA (dsRNA) viruses**	3	N/A	N/A	3	N/A	N/A	N/A	N/A	N/A	N/A
**Reoviridae**	3	N/A	N/A	3	N/A	N/A	N/A	N/A	N/A	N/A
Fusogenic orthoreovirus	3	N/A	N/A	3	N/A	N/A	N/A	N/A	N/A	N/A
**Class IV: Single stranded RNA (ssRNA) viruses**	37,886	257	853	2,535	2,086	20	336	1,515	N/A	2,083
**Caliciviridae**	23	N/A	N/A	N/A	N/A	N/A	N/A	N/A	N/A	N/A
Norovirus	23	N/A	N/A	N/A	N/A	N/A	N/A	N/A	N/A	N/A
**Coronaviridae**	35,117	248	809	2,386	2,052	8	332	1,515	N/A	441
HCoV-229E	176	1	N/A	2	N/A	N/A	N/A	N/A	N/A	171
HCoV-HKU1	23	N/A	N/A	N/A	N/A	N/A	N/A	N/A	N/A	N/A
HCoV-NL63	37	N/A	N/A	N/A	N/A	N/A	N/A	N/A	N/A	9
HCoV-OC43	79	N/A	N/A	1	N/A	N/A	5	N/A	N/A	9
MERS-CoV	53	1	N/A	52	N/A	N/A	N/A	N/A	N/A	N/A
SARS-CoV-1	1,263	N/A	1	22	N/A	N/A	25	N/A	N/A	N/A
SARS-CoV-2	33,258	246	800	2,309	2,052	8	302	1,515	N/A	135
**Parechovirus**	2,230	7	43	141	34	12	4	N/A	N/A	1,267

Molecular diagnostic	Total cases (n)	Area of acquisition (continent)					Specific circumstances of acquisition			
		Africa	Americas	Asia	Europe	Oceania	Airplane	Cruise or Merchant Vessel	Refugee and Asylum-Seeker	Mass gatherings events[a]
Rhinovirus	2,229	7	43	141	34	12	4	N/A	N/A	1,266
HRV-A	12	N/A	N/A	N/A	N/A	N/A	N/A	N/A	N/A	N/A
HRV-B	4	N/A	N/A	N/A	N/A	N/A	N/A	N/A	N/A	N/A
**Picornaviridae**	165	2	1	8	N/A	N/A	N/A	N/A	N/A	24
Enterovirus	107	2	1	2	N/A	N/A	N/A	N/A	N/A	24
**Class V: Single stranded RNA (-ssRNA) viruses**	23,211	195	824	4,552	680	473	106	341	N/A	2,366
**Hantaviridae**	25	N/A	12	N/A	N/A	N/A	N/A	N/A	N/A	N/A
**Orthomyxoviridae**	22,683	191	806	4,541	680	473	77	341	N/A	2,218
Influenza	22,683	191	806	4,541	680	473	77	341	N/A	2,218
Influenza A virus (IAV)	6,384	25	148	255	74	42	65	233	N/A	1,271
H1N1 (A/H1N1)	5,091	10	117	83	74	42	54	78	N/A	615
H3N2 (A/H3N2)	644	13	19	41	N/A	N/A	N/A	100	N/A	425
H5N1 (A/H5N1)	2	N/A	N/A	2	N/A	N/A	N/A	N/A	N/A	N/A
H7N9 (A/H7N9)	1	N/A	N/A	1	N/A	N/A	N/A	N/A	N/A	N/A
Influenza B virus (IBV)	760	2	15	70	N/A	N/A	12	12	N/A	544
B/Brisbane	18	1	5	5	N/A	N/A	N/A	7	N/A	N/A
B/Florida	16	1	5	6	N/A	N/A	N/A	N/A	N/A	4
B/Malaysia	13	N/A	N/A	N/A	N/A	N/A	N/A	N/A	N/A	13
**Paramyxoviridae**	217	2	4	6	N/A	N/A	24	N/A	N/A	49
Human parainfluenza viruses (HPIVs)	217	2	4	6	N/A	N/A	24	N/A	N/A	49
HPIV-1	22	N/A	N/A	N/A	N/A	N/A	N/A	N/A	N/A	20
HPIV-2	8	N/A	N/A	1	N/A	N/A	N/A	N/A	N/A	7
HPIV-3	34	N/A	1	4	N/A	N/A	N/A	N/A	N/A	17
HPIV-4	4	2	N/A	1	N/A	N/A	N/A	N/A	N/A	1
**Pneumoviridae**	3	N/A	N/A	N/A	N/A	N/A	N/A	N/A	N/A	3
Human metapneumovirus (HMPV)	75	N/A	1	1	N/A	N/A	4	N/A	N/A	10
Respiratory syncytial virus (RSV)	211	2	1	4	N/A	N/A	1	N/A	N/A	89
RSV-A	7	2	1	1	N/A	N/A	N/A	N/A	N/A	2
RSV-B	3	N/A	N/A	N/A	N/A	N/A	N/A	N/A	N/A	3

Molecular diagnostic	Total cases (n)	Area of acquisition (continent)					Specific circumstances of acquisition			
		Africa	Americas	Asia	Europe	Oceania	Airplane	Cruise or Merchant Vessel	Refugee and Asylum-Seeker	Mass gatherings events[a]
**BACTERIA**	3,863	23	N/A	9	1	N/A	2	1	134	2,717
**Gram-positive bacteria**	1,644	4	N/A	4	1	N/A	N/A	1	134	1,274
**Rod (bacilli)**	49	2	N/A	N/A	N/A	N/A	N/A	N/A	47	N/A
**Corynebacteriaceae**	2	1	N/A	N/A	N/A	N/A	N/A	N/A	1	N/A
Corynebacterium diphtheriae	2	1	N/A	N/A	N/A	N/A	N/A	N/A	1	N/A
**Mycobacteriaceae**	47	1	N/A	N/A	N/A	N/A	N/A	N/A	46	N/A
Mycobacterium africanum	46	N/A	N/A	N/A	N/A	N/A	N/A	N/A	46	N/A
Mycobacterium kansasii	1	1	N/A	N/A	N/A	N/A	N/A	N/A	N/A	N/A
**spherical (cocci)**	1,595	2	N/A	4	1	N/A	N/A	1	87	1,274
**Enterococcaceae**	4	N/A	N/A	N/A	N/A	N/A	N/A	N/A	N/A	4
Enterococcus	4	N/A	N/A	N/A	N/A	N/A	N/A	N/A	N/A	4
**Staphylococcaceae**	840	1	N/A	2	N/A	N/A	N/A	1	87	656
Coagulase-negative staphylococci (CoNS)	3	N/A	N/A	N/A	N/A	N/A	N/A	N/A	N/A	N/A
Staphylococcus aureus	745	1	N/A	2	N/A	N/A	N/A	1	N/A	653
Methicillin-resistant Staphylococcus aureus	92	N/A	N/A	N/A	N/A	N/A	N/A	N/A	87	3
**Streptococcaceae**	751	1	N/A	2	1	N/A	N/A	N/A	N/A	614
Streptococcus dysgalactiae	1	N/A	N/A	1	N/A	N/A	N/A	N/A	N/A	N/A
Streptococcus Group G	3	N/A	N/A	N/A	N/A	N/A	N/A	N/A	N/A	2
Streptococcus pyogenes	58	N/A	N/A	1	N/A	N/A	N/A	N/A	N/A	34
Streptococcus pneumoniae	689	1	N/A	N/A	1	N/A	N/A	N/A	N/A	578
**Gram-negative bacterium**	2,219	19	N/A	5	N/A	N/A	2	N/A	N/A	1,443
**Pleomorphic**	48	3	N/A	N/A	N/A	N/A	1	N/A	N/A	2
**Mycoplasmataceae**	48	3	N/A	N/A	N/A	N/A	1	N/A	N/A	2
Mycoplasme pneumoniae	48	3	N/A	N/A	N/A	N/A	1	N/A	N/A	2
**rod (bacilli)**	2,029	1	N/A	5	N/A	N/A	N/A	N/A	N/A	1,327
**Burkholderiaceae**	1	1	N/A	N/A	N/A	N/A	N/A	N/A	N/A	N/A
Burkholderia gladioli	1	1	N/A	N/A	N/A	N/A	N/A	N/A	N/A	N/A
**Chlamydiaceae**	64	N/A	N/A	N/A	N/A	N/A	N/A	N/A	N/A	58
Chlamydophila pneumoniae	64	N/A	N/A	N/A	N/A	N/A	N/A	N/A	N/A	58

Molecular diagnostic	Total cases (n)	Area of acquisition (continent)					Specific circumstances of acquisition			
		Africa	Americas	Asia	Europe	Oceania	Airplane	Cruise or Merchant Vessel	Refugee and Asylum-Seeker	Mass gatherings events[a]
**Enterobacteriaceae**	595	N/A	N/A	3	N/A	N/A	N/A	N/A	N/A	550
Escherichia coli	3	N/A	N/A	N/A	N/A	N/A	N/A	N/A	N/A	3
Klebsiella pneumoniae	592	N/A	N/A	3	N/A	N/A	N/A	N/A	N/A	547
**Moraxellaceae**	237	N/A	N/A	N/A	N/A	N/A	N/A	N/A	N/A	237
Acinetobacter baumannii	12	N/A	N/A	N/A	N/A	N/A	N/A	N/A	N/A	12
Moraxella catarrhalis	225	N/A	N/A	N/A	N/A	N/A	N/A	N/A	N/A	225
**Pasteurellaceae**	1,101	N/A	N/A	1	N/A	N/A	N/A	N/A	N/A	452
Haemophilus influenzae	1,081	N/A	N/A	1	N/A	N/A	N/A	N/A	N/A	432
Haemophilus parainfluenzae	20	N/A	N/A	N/A	N/A	N/A	N/A	N/A	N/A	20
**Pseudomonadaceae**	30	N/A	N/A	1	N/A	N/A	N/A	N/A	N/A	29
Pseudomonas aeruginosa	30	N/A	N/A	1	N/A	N/A	N/A	N/A	N/A	29
**Xanthomonadaceae**	1	N/A	N/A	N/A	N/A	N/A	N/A	N/A	N/A	1
Stenotrophomonas maltophilia	1	N/A	N/A	N/A	N/A	N/A	N/A	N/A	N/A	1
**Spherical (cocci)**	142	15	N/A	N/A	N/A	N/A	1	N/A	N/A	114
**Neisseriaceae**	142	15	N/A	N/A	N/A	N/A	1	N/A	N/A	114
Neisseria meningitidis	142	15	N/A	N/A	N/A	N/A	1	N/A	N/A	114
Neisseria meningitidis W-135	98	1	N/A	N/A	N/A	N/A	N/A	N/A	N/A	96
**FUNGI**	29	N/A	1	N/A	N/A	1	N/A	N/A	N/A	27
**Ascomycota**	28	N/A	N/A	N/A	N/A	1	N/A	N/A	N/A	27
**Pleosporaceae**	1	N/A	N/A	N/A	N/A	1	N/A	N/A	N/A	N/A
Cochliobolus hawaiiensis	1	N/A	N/A	N/A	N/A	1	N/A	N/A	N/A	N/A
**Saccharomycetaceae**	27	N/A	N/A	N/A	N/A	N/A	N/A	N/A	N/A	27
Candida albicans	27	N/A	N/A	N/A	N/A	N/A	N/A	N/A	N/A	27
**Basidiomycota**	1	N/A	1	N/A	N/A	N/A	N/A	N/A	N/A	N/A
**Cryptococcaceae**	1	N/A	1	N/A	N/A	N/A	N/A	N/A	N/A	N/A
Cryptococcus deuterogattii	1	N/A	1	N/A	N/A	N/A	N/A	N/A	N/A	N/A

Includes cases of:[a]AsiaWorld-Expo, Bābā Farīd, Easter Festival (Carinthia), Eco-Challenge, EXIT Festival, Grand Magal of Touba, Hajj, Rock Werchter, Sojourn, Sziget Festival, Tablighi Jamaat,Umrah, Universiade, Winter Olympic & Paralympic Games, World Youth Day

Appendix (section 6). Absolute frequency of respiratory infections cases in travellers for the four respiratory pandemics of the 21st century.

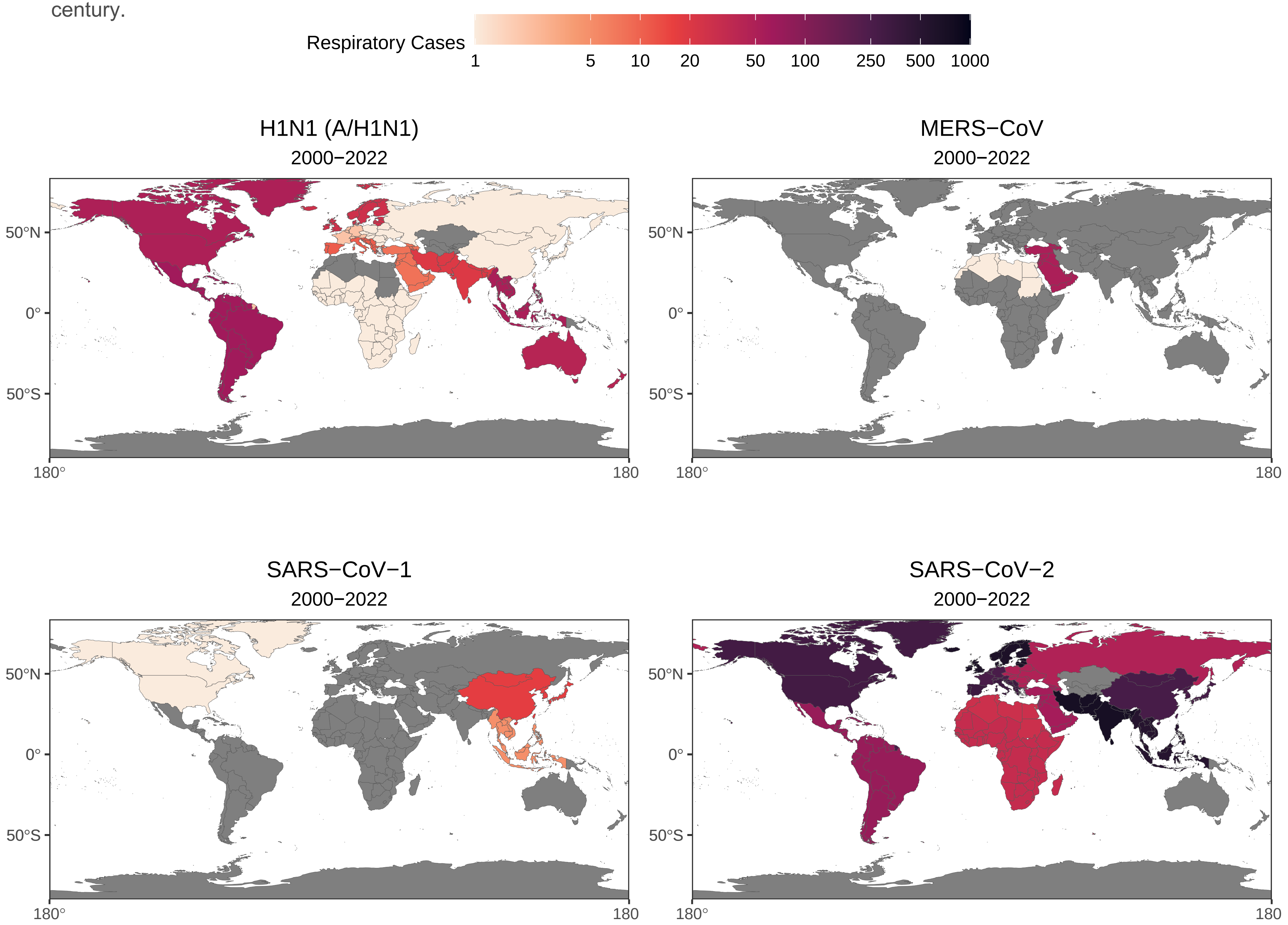

Supplement: Appendix_taad081 [file appendix_taad081.pdf]
